# A Comparison of Genome-Wide DNA Methylation Patterns between Different Vascular Tissues from Patients with Coronary Heart Disease

**DOI:** 10.1371/journal.pone.0122601

**Published:** 2015-04-09

**Authors:** Maria S. Nazarenko, Anton V. Markov, Igor N. Lebedev, Maxim B. Freidin, Aleksei A. Sleptcov, Iuliya A. Koroleva, Aleksei V. Frolov, Vadim A. Popov, Olga L. Barbarash, Valery P. Puzyrev

**Affiliations:** 1 Laboratory of Population Genetics, Research Institute of Medical Genetics, Tomsk, Russian Federation; 2 Laboratory of Cytogenetics, Research Institute of Medical Genetics, Tomsk, Russian Federation; 3 Laboratory of Human Ontogenetics, Tomsk State University, Tomsk, Russian Federation; 4 Laboratory of Neurovascular Pathology, Research Institute for Complex Problems of Cardiovascular Diseases, Kemerovo, Russian Federation; 5 Department of Multifocal Atherosclerosis, Research Institute for Complex Problems of Cardiovascular Diseases, Kemerovo, Russian Federation; University of Michigan, UNITED STATES

## Abstract

Epigenetic mechanisms of gene regulation in context of cardiovascular diseases are of considerable interest. So far, our current knowledge of the DNA methylation profiles for atherosclerosis affected and healthy human vascular tissues is still limited. Using the Illumina Infinium Human Methylation27 BeadChip, we performed a genome-wide analysis of DNA methylation in right coronary artery in the area of advanced atherosclerotic plaques, atherosclerotic-resistant internal mammary arteries, and great saphenous veins obtained from same patients with coronary heart disease. The resulting DNA methylation patterns were markedly different between all the vascular tissues. The genes hypomethylated in athero-prone arteries to compare with atherosclerotic-resistant arteries were predominately involved in regulation of inflammation and immune processes, as well as development. The great saphenous veins exhibited an increase of the DNA methylation age in comparison to the internal mammary arteries. Gene ontology analysis for genes harboring hypermethylated CpG-sites in veins revealed the enrichment for biological processes associated with the development. Four CpG-sites located within the *MIR10B* gene sequence and about 1 kb upstream of the *HOXD4* gene were also confirmed as hypomethylated in the independent dataset of the right coronary arteries in the area of advanced atherosclerotic plaques in comparison with the other vascular tissues. The DNA methylation differences observed in vascular tissues of patients with coronary heart disease can provide new insights into the mechanisms underlying the development of pathology and explanation for the difference in graft patency after coronary artery bypass grafting surgery.

## Introduction

Atherosclerosis underlies the development of a majority of cardiovascular diseases, which are among the leading causes of mortality worldwide. Different vascular beds vary in their susceptibility to the development of atherosclerosis. Coronary artery has the highest prevalence of atherosclerotic plaques in comparison with other arteries. It has been established that internal mammary arteries as well as great saphenous veins are resistant to the development of atherosclerosis [1]. However, the outcome of venous bypass grafts is poor because veins are more prone to occlusive disease than artery grafts. Understanding of the mechanisms of vascular differences in disease development may yield insight into factors that affect atherosusceptibility as well as disease progression.

Although atherosclerosis is caused by the interaction of multiple genetic and environmental factors, these explain only a portion of the total disease risk. Epigenetic mechanisms that underly this pathology have become a promising area of research [2, 3]. Compared to genetic factors, epigenetic variation is much more suitable to explain the progressive and age-related nature of atherosclerosis characterized by sex and tissue specificity. Aberrant epigenetic patterns can be acquired during developmental stages under environmental influence.

The most widely studied and best-characterized epigenetic marker in human genome is DNA methylation. DNA methylation in tissues usually occurs within the context of CpG-dinucleotide sequences (CpG-sites). In somatic mammalian cells, the majority of CpG-sites are methylated. However, CpG-sites located in regions of increased CG-density, known as CpG-islands, generally have low levels of methylation. DNA methylation at gene promoters is important for transcriptional regulation, with dense promoter hypermethylation around the transcription start sites being associated with repressed expression of genes. Outside of CpG-islands, intragenic DNA methylation has been linked to transcriptional and splicing activities [4].

In vascular tissues of patients with atherosclerosis, DNA methylation alterations of 15-lipoxygenase (*ALOX1*5), estrogen receptors (*ESR1* and *ESR2*), monocarboxylate transporter 3 (*MCT3*), and tissue factor pathway inhibitor 2 (*TFPI2*) genes have been reported using a candidate gene approach [5–9]. Using high throughput microarray technology, genome-wide DNA methylation patterns can be evaluated simultaneously, and novel tissue-specific molecular targets can be identified. However, our knowledge about the impact of genome-wide alterations of DNA methylation on the atherosclerosis phenotype in humans is still limited [10–12].

DNA methylation pattern in many tissues is highly correlated with the chronological age. Recently, Horvath [13] developed a multi-tissue predictor of DNA methylation age. He proposed that DNA methylation age measures the cumulative work done by the epigenetic maintenance system and epigenetic clock can be used to identify tissues affected by disease [13].

In the current study we performed a comparative analysis of DNA methylation patterns in right coronary arteries in the area of advanced atherosclerotic plaques (CAP), internal mammary arteries (IMA), and great saphenous veins (GSV) derived from the same individuals with coronary artery atherosclerosis, using Illumina HumanMethylation27 BeadChip microarrays. We hypothesize that DNA methylation differences are the key to distinguish CAP from the IMA with respect to their liability to the development of atherosclerosis. A comparison of DNA methylation patterns between GSV and IMA can provide an explanation for the difference in graft patency after coronary artery bypass grafting surgery.

## Materials and Methods

### Sample collection and processing

A total of 21 Russian men (age 57.76 ± 6.9 years, mean ± S.E.) who underwent surgery for severe coronary artery stenosis were recruited (Table 1). Sixteen patients (76.2%) had a history of heart failure prior to surgery. Hypertension was diagnosed in all patients. Fourteen men (66.7%) had hyperlipidaemia. Diabetes mellitus was found in 4 patients (19.0%). The study protocol was approved by the Ethical Committee of the Research Institute for Medical Genetics and written informed consents were obtained from all the participants.

**Table 1:** Baseline characteristics of the study subjects.

Clinical parameters	All patients, n = 21
**Age (years)**	57.76±6.9
**BMI (kg m** ^−2^ **)**	29.68±3.9
**Heart attack**	16 (76.2)
**Hypertension**	21 (100)
**Smoking**	12 (57.1)
**Diabetes mellitus**	4 (19.0)
**Glucose (mmol/L)**	6.29±1.4
**Cholesterol (mmol/L)**	5.62±1.1
**Triglycerides (mmol/L)**	2.14 (0.81–9.03)
**Apolipoprotein B/apolipoprotein A1 ratio**	4.2 (2.53–7.70)

Hypertension was defined as a systolic blood pressure ≥140 or diastolic ≥90mm Hg or the current use of antihypertensive drugs. Patients were categorized as smokers if they were current smokers or had stopped smoking for less than 1 year. Obesity was appreciated by the Body Mass Index (BMI). Quantitative values are presented as mean±S.E. or median (min-max) and qualitative values as n (percentage).

Matched biopsy specimens were obtained from the right coronary artery in the area of advanced atherosclerotic plaques (CAP), internal mammary arteries (IMA) and great saphenous veins (GSV) of the patients undergoing coronary artery bypass graft surgery. The atherosclerotic lesions of right coronary artery were classified as advanced according to surgeon recommendations. Immediately after the operation, samples were examined by a pathologist, cleaned from calcifications, fatty deposits and thrombotic material, and washed with sterile physiological saline solution. All samples were snap-frozen in liquid nitrogen and stored at -80°C until further use.

In our study several vascular tissue samples were stained with hematoxylin and eosin or immunostained with antibodies against smooth muscle-specific α-actin and CD68. Smooth muscle cells were predominated in all analyzed vascular tissues. CAP, IMA and GSV showed a variable degree of infiltration with macrophages. Specimens taken from the CAP contained a large accumulation of macrophages to compare with IMA and GSV. For DNA methylation microarray profiling, we selected match-paired specimens (CAP, IMA, GSV) from six patients. For confirmation by pyrosequencing and replication, match-paired tissue samples from all 21 individuals were used.

### DNA methylation profiling

The frozen tissue samples were homogenized using the Minilys homogenizer (Bertin Technologies) followed by isolation of DNA by standard proteinase K digestion and phenol/chloroform extraction method. Eighteen samples of genomic DNA were bisulfite converted using EZ DNA Methylation Kit (Zymo Research). DNA methylation level was measured using the Illumina Infinium Human Methylation27 BeadChip according to standard protocol [14]. Microarray’s probes interrogate the methylation state of 27578 individual CpG-sites located predominately in CpG-islands within proximal promoter regions, between 1.5 kb upstream and 1 kb downstream of the transcription start sites of 14475 consensus coding sequence genes throughout the genome. Furthermore 110 miRNA promoters are targeted with 254 CpG loci probes [14]. Bisulfite-converted genomic DNA was denatured, whole-genome amplified, fragmented and subsequently hybridized to a microarray. Fluorescently stained chip was scanned by the Illumina BeadArray Reader. Quality control was conducted in GenomeStudio software using the methylation module according to the manufacturer’s recommendations (Illumina). The data of DNA methylation in our study have been deposited in the Gene Expression Omnibus (GEO) database and are available through GSE62867 accession number (http://www.ncbi.nlm.nih.gov/geo/query/acc.cgi?acc=GSE62867).

### DNA pyrosequencing analysis

DNA methylation status of CpG-sites of *HOXD4* gene was assessed by pyrosequencing using PyroMark Q24 (Qiagen) according to the manufacturer’s instructions. Primers for pyrosequencing were designed to encompass the CpG-sites assayed on the Illumina Infinium array.

The region studied for the differential DNA methylation encompasses four CpG-dinucleotides located within the *MIR10B* gene sequence and about 1 kb upstream of the *HOXD4* gene (chr 2q31.1; CpG-site 1 (cg01152019): 177,015,044, CpG-site 2 (cg14399060): 177,015,070, CpG-site 3: 177,015,088, and CpG-site 4: 177,015,104; UCSC Genome Browser Ch37/hg19).

For the bisulfite PCR, 40–50 ng of bisulfite-converted DNA was amplified using 2 U Hot Start Taq DNA polymerase (SibEnzyme) and 0.2 μM forward (5’-GGTTATTTGAATTGTTTTAGAAAG-3’) and reverse (5’-[Biotin]- CACTTTAATCTCTAACTATTCC-3’) primers in 50 μl reaction volume including 0.2 mM dNTPs and 2 mM MgCl_2_. The PCR conditions were: 95°C for 5 min followed by 42 cycles of 95°C for 30 s, 56°C for 45 s, 72°C for 30 s, and final elongation step at 72°C for 5 min. Biotin-labeled single-stranded amplicons were retrieved and subjected to pyrosequencing with use of 0.3 μM sequencing primer 5’-TTTTGGGTGGGATTTAGAGGTTGT-3’ according to the manufacturer’s protocol. The percent of methylation for each of the CpGs within the target sequence was calculated using PyroQ CpG Software (Qiagen). Non-CpG cytosine residues were used as built-in controls to verify bisulfite conversion. Each marker was tested in two replicates and their average was used in the statistical analysis.

### Statistical analysis

The results presented in our study were obtained using the log2 ratio between unmethylated and methylated probes (M-value) and the beta-statistics [methylated/(unmethylated + methylated)]. Illumina’s GenomeStudio software was used to analyze BeadArray data to assign site-specific DNA methylation beta-values to each CpG-site. The beta-value represents a quantitative measure of the DNA methylation level of specific CpG-sites and ranges from 0 (completely unmethylated) to 1 (completely methylated).

The raw data were imported into R environment for statistical computing v3.0.1 (http://www.R-project.org) and analyzed using Bioconductor *lumi* package [15]. The data were further inspected for quality metrics, adjusted for color channel imbalance and quantile-normalized. All probes with detection p-values ≥0.01 were removed.

DNA methylation age of tissues was calculated using R-code developed by Horvath (2013). Multi-dimensional scaling was used to display the distance between individual samples based on their methylation status. All CpG-sites were used for scaling.

Differentially methylated CpG-sites (expressed as M-values) between groups were identified using *limma* package [16]. Benjamini-Hochberg false-discovery rate (FDR) method was applied to control for multiple tests. FDR-adjusted p<0.05 and delta beta ≥0.2 or delta beta ≤-0.2 were used as cut-off values as indicated in the volcano plot. Heatmap of all samples was drawn with samples in rows and CpG-sites in column. For clearer presentation of differentially methylated CpG-sites and genes, we chose a cutoff value of 0.4 for the heatmaps instead of the 0.2 set for the analysis.

Functional enrichment analysis of genes containing differentially methylated CpG-sites was performed using the web-based GEne SeT AnaLysis Toolkit [17]. The reference gene set comprised the genes represented on the Illumina arrays. Gene Ontology (GO) categories were found by a hypergeometric statistical test. p-values were corrected for multiple testing using FDR.

Statistically significant differences in DNA methylation level between groups were identified by ANOVA with post-hoc test. An enrichment of hyper- and hypomethylated CpG-sites versus all CpG-sites was calculated using Fisher’s exact test. Correlations between variables were estimated using Pearson’s correlation coefficient. The significance level of p<0.05 was applied. These analyses were carried out using GraphPad software (InStat).

## Results

**Fig 1:**
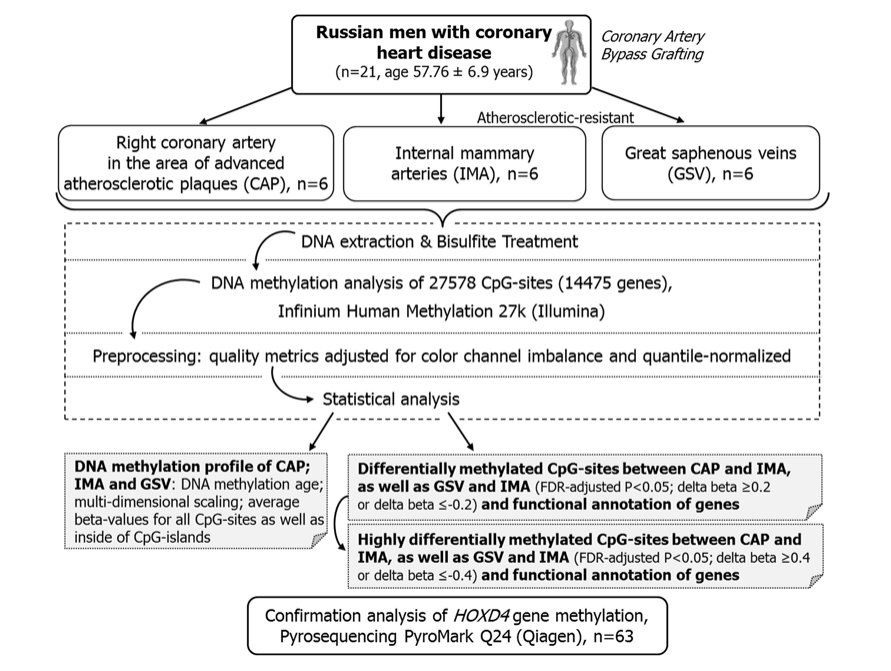
Flow chart describing experiment and analysis.

The pipeline of the experiment is given in Fig 1.

### DNA methylation profile of vascular tissues

The analysis of DNA methylation profiles in four matched sets of tissues from 6 individuals was performed using Infinium HumanMethylation27 BeadChip assay (Illumina), which contains 27578 CpG targets covering 14495 genes. After quality control, methylation of 27378 CpG-sites (14391 genes) was analyzed.

**Fig 2:**
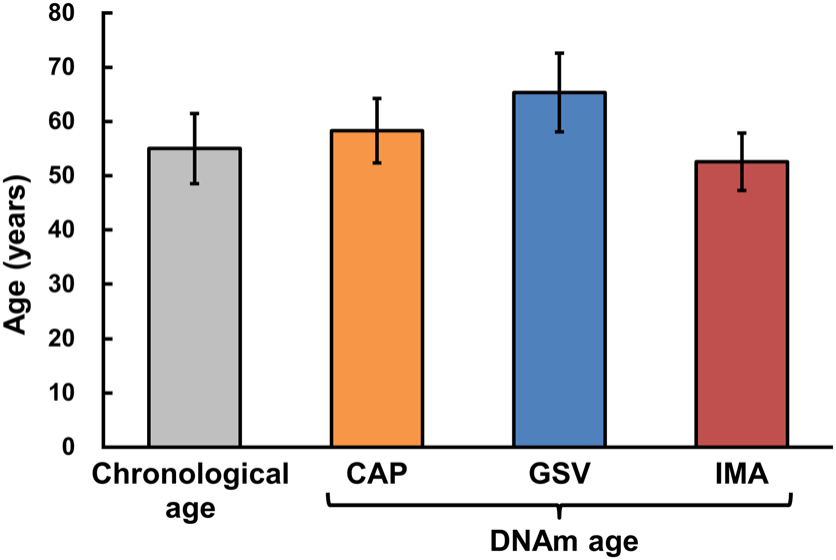
The mean chronological age and DNA methylation age (DNAm) of vascular tissues: right coronary artery in the area of advanced atherosclerotic plaques (CAP), internal mammary arteries (IMA), and great saphenous veins (GSV).

DNA methylation age per tissue was moderately correlated with corresponding chronological age (r = 0.67, 95%CI 0.36–0.84, p = 0.0004). However, GSV showed evidence of significantly higher mean DNA methylation age than IMA (65.3±7.3 vs. 52.7±5.2, respectively; p<0.05; Fig 2).

**Fig 3:**
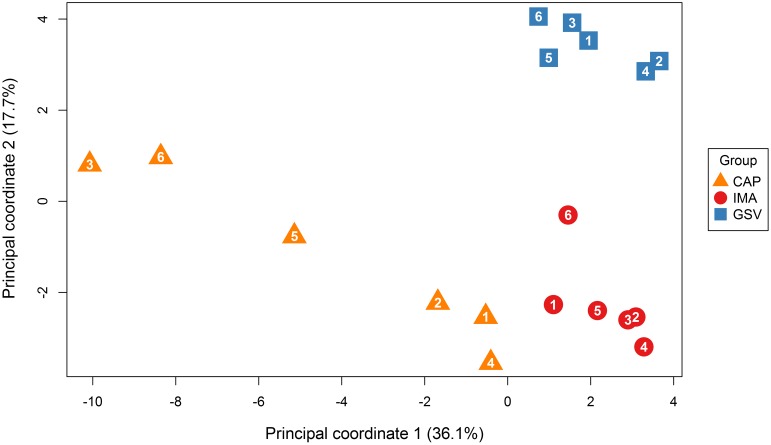
Multi-dimensional scaling for genome-wide methylation of right coronary artery in the area of advanced atherosclerotic plaques (CAP), internal mammary arteries (IMA), and great saphenous veins (GSV). Arabic numbers are sample IDs.

Multi-dimensional scaling plot revealed that the DNA methylation patterns differed more between tissue types than between individuals (Fig 3). In terms of DNA methylation, IMA and GSV were closer to each other than to CAP. The IMA and GSV tissues showed minimal differences between individual methylation profiles, whereas the CAP was characterized by high interindividual variability of methylation profiles.

The average beta-values for all CpG-sites as well as inside of CpG-islands were higher in the CAP tissues than in the IMA and GSV (Table 2). The hypomethylated CpG-sites (beta-value≤0.2) were registered less frequently in CAP (0.65) as compared to IMA (0.67; p<0.05).

**Table 2:** Comparison of β-values (average ± s.d.) in paired vascular tissues from six patients with atherosclerosis.

CG-sites	CG-sites count (%)	CAP, n = 6	IMA, n = 6	Methylation differences CAP vs. IMA (p-value)	GSV, n = 6	Methylation differences CAP vs. GSV (p-value)
**All**	27378 (100)	0.252±0.03	0.247±0.02	0.005 (<0.001)	0.250±0.02	0.002 (<0.001)
**Inside GpG-islands**	19993 (73)	0.129±0.02	0.124±0.01	0.005 (<0.001)	0.126±0.01	0.003 (<0.001)
**Outside CpG-islands**	7385 (27)	0.584±0.05	0.583±0.03	0.001 (>0.05)	0.584±0.03	0 (>0.05)

CAP indicates right coronary arteries in the area of advanced atherosclerotic plaques; IMA, internal mammary arteries; GSV, great saphenous veins.

### Differentially methylated CpG-sites for CAP and IMA tissues

**Fig 4:**
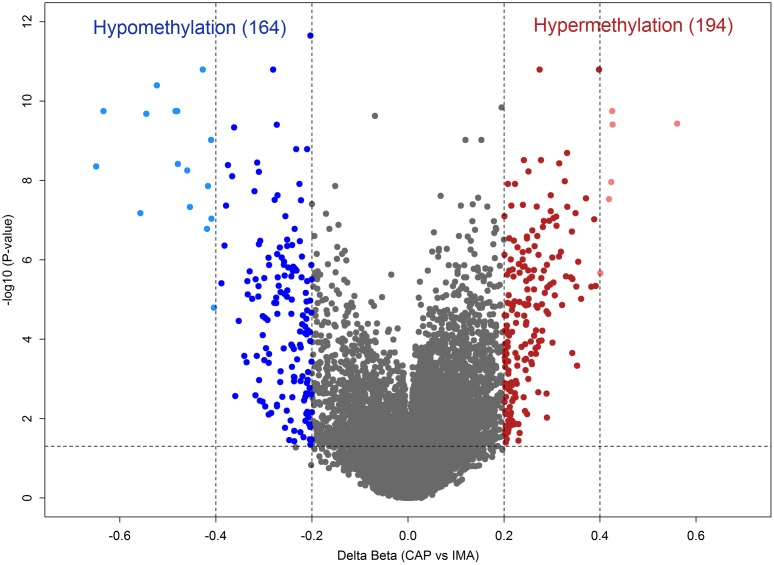
Volcano plot of - log10 (P-value) against delta beta value, representing the methylation difference between right coronary arteries in the area of advanced atherosclerotic plaques (CAP) and internal mammary arteries (IMA). A total of 194 CpG-sites hypermethylated in CAP with a delta beta ≥ 0.2 and FDR-adjusted p<0.05 are shown in red. A total of 164 CpG-sites hypomethylated in CAP with a delta beta ≤ -0.2 and FDR-adjusted p<0.05 are shown in blue. CpG-sites that exhibited a methylation level difference less 20% are shown in grey. Light red and light blue colors indicate highly differentially methylated CpG-sites (delta beta ≥ 0.40 or delta beta ≤ -0.40 with FDR-adjusted p<0.05). Dashed lines indicate cut-offs for significance.

Differential methylation analysis between CAP and IMA tissues revealed 358 CpG-sites exhibited a methylation level difference as little as 0.2 (S1 Table). Among these differentially methylated CpG-sites, 164 (45.8%) were hypomethylated in CAP as compared to IMA as indicated in the volcano plot (Fig 4). GO analysis revealed that these CpG-sites mainly located within the genes involved in regulation of inflammation and immune processes, as well as development (S2 Table). By contrast, no statistically significant GOs were found for the genes harboring 194 (54.2%) hypermethylated sites.

**Fig 5:**
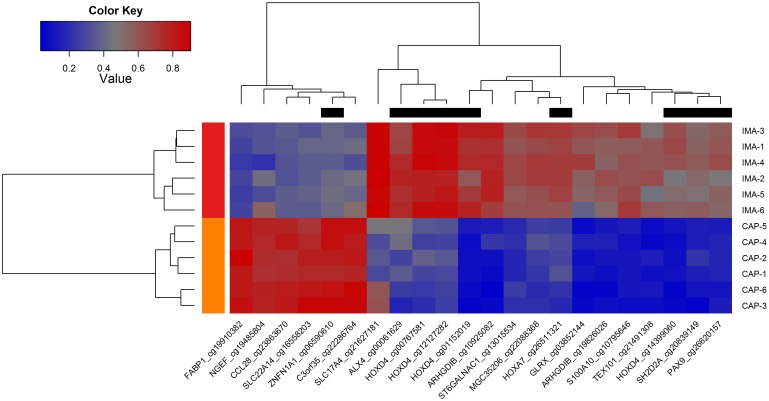
Heatmap analysis of 22 highly differentially methylated CpG-sites (delta beta ≥ 0.40 or delta beta ≤ -0.40 with FDR-adjusted p<0.05) between right coronary arteries in the area of advanced atherosclerotic plaques (CAP) and internal mammary arteries (IMA). Regions shaded blue in the heat map represent hypomethylated regions, regions shaded red represent hypermethylated regions. The top black rectangles shows columns representing CpG-sites located inside CpG-island. Gene symbols and CpG-site IDs are shown on the bottom. Sample IDs are on the right.

Within the list of 358 CpG-sites, there were 22 loci covering 18 genes with the greater magnitude of differential methylation (delta beta ≥0.40 or ≤-0.40) (Figs 4 and 5). The hypomethylated genes in CAP include *ARHGDIB*, *HOXD4*, *S100A10*, *GLRX*, *TEX101*, *SLC17A4*, *ST6GALNAC1*, *PAX9*, *MGC35206*, *HOXA7*, *SH2D2A*, and *ALX4*; the hypermethylated genes include *NGEF*, *SLC22A14*, *C3orf35*, *ZNFN1A1*, *CCL28*,and *FABP1*.

### Differentially methylated CpG-sites for GSV and IMA tissues

**Fig 6:**
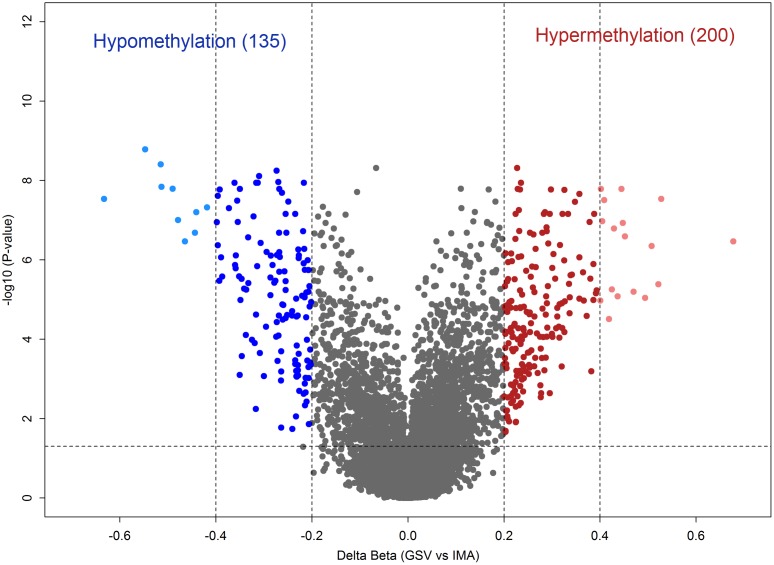
Volcano plot of - log10 (P-value) against delta beta value, representing the methylation difference between great saphenous veins (GSV) and internal mammary arteries (IMA). A total of 200 CpG-sites hypermethylated in GSV with a delta beta ≥ 0.2 and FDR-adjusted p<0.05 are shown in red. A total of 135 CpG-sites hypomethylated in GSV with a delta beta ≤ -0.2 and FDR-adjusted p<0.05 are shown in blue. CpG-sites that exhibited a methylation level difference less 20% are shown in grey. Light red and light blue colors indicate highly differentially methylated CpG-sites (delta beta ≥ 0.40 or delta beta ≤ -0.40 with FDR-adjusted p<0.05). Dashed lines indicate cut-offs for significance.

A statistically significant difference in DNA methylation levels between IMA and GSV tissues was identified for 335 CpG-sites (Fig 6; S3 Table). Among all differentially methylated CpG-sites, 200 (59.7%) were hypermethylated in GSV compared to IMA. Gene ontology analysis for genes harboring these CpG-sites revealed the enrichment for biological processes associated with the development (S4 Table).

**Fig 7:**
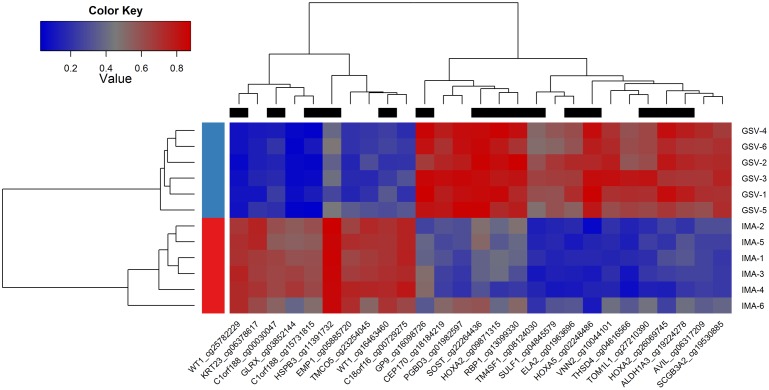
Heatmap analysis of 27 highly differentially methylated CpG-sites (delta beta ≥ 0.40 or delta beta ≤ -0.40 with FDR-adjusted p<0.05) between great saphenous veins (GSV) and internal mammary arteries (IMA). Regions shaded blue in the heat map represent hypomethylated regions, regions shaded red represent hypermethylated regions. The top black rectangles shows columns representing CpG-sites located inside CpG-island. Gene symbols and CpG-site IDs are shown on the bottom. Sample IDs are on the right.

Using heatmap cluster analysis, 16 hypermethylated (*HOXA5*, *HOXA2*, *PGBD3*, *VNN2*, *THSD4*, *ELA2*, *CEP170*, *SCGB3A2*, *TOM1L1*, *SULF1*, *ALDH1A3*, *GP9*, *AVIL*, *SOST*, *TM4SF1*, and *RBP1*) and 8 hypomethylated (*C1orf188*, *TMCO5*, *WT1*, *HSPB3*, *EMP1*, *C18orf16*, *GLRX*, and *KRT23*) genes were identified as significantly highly differentially methylated genes in the GSV (Figs 6 and 7).

### Pyrosequencing analysis at the promoter of *HOXD4* gene in vascular tissues

The maximal beta-value difference was detected between the CAP and IMA tissues for the CpG-site cg01152019 in promoter of the *HOXD4* gene. Represented on the array with reliable detection signals, a total of five CpG-sites were located about 1 kb upstream of the *HOXD4* gene.

To verify the methylation level of the *HOXD4* gene promoter, DNA from match-paired vascular tissues of 15 additional patients, along with the samples used for the methylation profiling in the array, was subjected to bisulfite treatment followed by pyrosequencing.

The methylation values for 18 tissue samples obtained using Infinium array and pyrosequencing were found to be significantly correlated (for the CpG-site cg01152019 Pearson’s R = 0.88, p<0.05).

Methylation levels in CAP tissues were significantly different from the average methylation of other tissues (Table 3). Four CpG-sites in the *HOXD4* promoter region were also confirmed as hypomethylated in the independent dataset of CAP in comparison with IMA and GSV.

**Table 3:** Methylation level (mean ±SD) at the promoter of *HOXD4* gene in paired vascular tissues from twenty one patients with atherosclerosis.

CG-site (Illumina IDs)	CAP, n = 21	IMA, n = 21	Methylation differences CAP vs. IMA (p-value)	GSV, n = 21	Methylation differences CAP vs. GSV (p-value)
**CG1 (cg01152019)**	0.129±0.04	0.610±0.08	-0.481 (<0.001)	0.620±0.04	-0.491 (<0.001)
**CG2 (cg14399060)**	0.130±0.02	0.485±0.07	-0.355 (<0.001)	0.408±0.06	-0.278 (<0.001)
**CG3**	0.258±0.06	0.686±0.09	-0.428 (<0.001)	0.726±0.05	-0.468 (<0.001)
**CG4**	0.226±0.05	0.620±0.13	-0.394 (<0.001)	0.526±0.06	-0.3 (<0.001)
**All CG**	0.186±0.04	0.600±0.07	-0.414 (<0.001)	0.571±0.04	-0.385 (<0.001)

CAP indicates right coronary arteries in the area of advanced atherosclerotic plaques; IMA, internal mammary arteries; GSV, great saphenous veins.

## Discussion

Aberrant DNA methylation has been recognized as an important factor underlying the development and progression of in various common diseases. A majority of studies conducted so far have focused on cancer and only a few have investigated the role of epigenetic mechanisms, such as DNA methylation, in the development of cardiovascular disease [3]. To the best of our knowledge, comparative DNA methylation profiles of affected and healthy vascular tissues had not been previously investigated in detail.

We analyzed quantitative single-base resolution DNA methylation patterns on a genome-wide level in vascular tissues obtained from patients with atherosclerosis. We showed that DNA methylation age of vascular tissues was moderately related to chronological age. The great saphenous veins had increased DNA methylation age in comparison to the internal mammary arteries. It has been established elsewhere, that GSV is a more differentiated tissue with lower rates of cell proliferation and apoptosis as compared to IMA [18]. It is possible that high DNA methylation age of GSV may contribute to abnormal adaptation after coronary artery bypass grafting surgery.

We observed a high heterogeneity of DNA methylation profiles of the CAP tissues. A plaque constitutes many cell types and other components and, therefore, our finding was not surprising. The average methylation level for all the CpG-sites as well as inside the CpG-islands was significantly higher in the CAP tissues, mainly due to lower frequency of the hypomethylated CpG-sites, as compared with the IMA. Our results contradict to those published by Castillo-Diaz et al. [10], who reported an extensive demethylation of normally hypermethylated CpG-islands in human atherosclerotic arteries. This may partially be due to the different microarray platforms used in the 2 studies. Castillo-Diaz et al. [10] used the single spotted array containing 14923 CpG-island clones of the UHN collections (Microarray Centre, Toronto, Canada), while our data were generated using Infinium Human Methylation27 BeadChip.

There is a body of evidences of a fundamental role of inflammation in the development and progression of atherosclerotic plaque [19]. To date, several microarray studies have reported on profiles of gene expression in human arteries, including advanced lesions and plaque-free areas [20]. The analysis of biological pathways in expression studies indicated overrepresentation of up-regulated genes involved in control of inflammation. Our data confirmed that genes hypomethylated in atherosclerotic-prone CAP tissues compared to atherosclerotic-resistant IMA tissues are involved in inflammation and immune response.

Intriguingly, a high percentage of differentially methylated genes between vascular tissues related to regulation of developmental process were revealed. One possible interpretation of our findings is that these genes are involved in vascular tissue remodeling. Reactivation of the developmental pathways is also needed during organ injury repair. Possibly, these genes are upstream effectors that modulate other pathways that are involved in atherosclerosis. It has been identified that the most highly variably DNA-methylated regions in several tissues were significantly enriched by the development-related genes [21]. According to the authors, stochastic epigenetic variation can be responsible for the development, evolutionary adaptation and disease susceptibility. Heterogeneity in the origins of vascular tissues could also contribute to the differences seen in developmental genes.

We also identified site-specific DNA methylation changes in comparison between the CAP and IMA as well as GSV and IMA tissues. In our study the majority of differentially methylated CpG-sites were located in developmental genes, including *HOX* cluster (*HOXA2*, *HOXA5*, *HOXA7*, *HOXD4*). Differential methylation region comparisons between athero-susceptible and protected sites in swine arterial endothelial methylome also revealed a sharp distinction in the *HOX* locus [22]. Aberrant homeobox CpG-island methylation is a frequent event in cancer [23], thus suggesting overlapping epigenetic dysregulation in cancer and atherosclerosis.

The *HOXD4* gene was found to be associated with differentially methylated CpG-island both in the study of Castillo-Diaz et al. [10] and in our study, However, Castillo-Diaz et al. [10] found that analyzed CpG-island was unmethylated in control, but hypermethylated in atherosclerotic artery. Conversely, we observed and confirmed by pyrosequencing a consistent hypomethylation of four CpG-sites located in the *HOXD4* promoter region in CAP tissues as compared to IMA and GSV. This result may be partly attributable to differences in the microarray platforms and the study subjects.

Although the functional significance of *HOXD4* in atherosclerosis remains unclear, a previous study identified differential expression of this gene in the samples of human aorta with varying degrees of atherosclerosis [24]. On the other hand, the region studied for the DNA methylation encompasses four CpG-dinucleotides located within *MIR10B* gene sequence. MiR-10b is a microRNA associated with metastasis and/or invasiveness of various cancer types [25]. It was shown that overexpression of miR-10b induces human microvascular endothelial cell migration and angiogenesis via down-regulation of homeobox D10 (HOXD10) [26]. This microRNA was also upregulated in advanced carotid plaques as compared with internal mammary artery [27]. Further studies will be required to elucidate the functional significance of methylation changes within *MIR10B* gene sequence in atherosclerosis.

*Hoxa2* and *Hoxa5* have been cloned from an adult rat vascular smooth muscle cDNA library, although the functions of these proteins were not determined in this study [28]. Hoxa5 is normally expressed in quiescent vessels, blocked angiogenesis and increased vascular stability [29]. Recently, it was shown that *HoxA5* is regulated by flow in a DNA methylation-dependent manner and that Hoxa5 regulates endothelial inflammation [30]. Induction of disturbed blood flow by partial carotid ligation surgery in a murine model resulted in hypermethylation within the promoter of *HoxA5* and downregulation of gene expression in arterial endothelium [30]. Our study shows that *HOXA5* is hypermethylated in great saphenous veins as compared with the internal mammary arteries that may contribute to aberrant remodeling of veins after coronary artery bypass grafting surgery.

Some of the highly differentially methylated genes are also very interesting. First, in our study *S100A10* gene was hypomethylated in right coronary artery in the area of advanced atherosclerotic plaques compared to atherosclerotic-resistant internal mammary arteries. S100A10 is highly expressed in endothelial cells, macrophages and foam cells of complicated carotid plaque segments [31]. This Ca^2+^-binding protein involved in the plasmin/plasminogen system regulating proteolytic activity and degradation of extracellular matrix, angiogenesis and macrophage invasion. Second, we observed changes in the methylation level of *GLRX* gene in both comparisons CAP vs. IMA and GSV vs. IMA. Expression of *GLRX* gene is enhanced in endothelial cells, smooth muscle cells and macrophages of human nonatherosclerotic and atherosclerotic coronary arteries [32]. Glutaredoxin-1 is a cytosolic enzyme that regulates diverse cellular functions. As speculated by Okuda et al. [32] glutaredoxin might be involved in the pathogenesis of atherosclerotic coronary heart disease via its antioxidant effect and/or its role as a signaling molecule.

Previous studies of vascular tissue samples revealed alterations in methylation of *ALOX15*, *ESR1*, *ESR2*, *MCT3* and *TFPI2* genes in patients with atherosclerosis [5–9]. These genes were covered by Infinium Human Methylation27 BeadChip, but they were not significantly differentially methylated in our study.

We have not tested functional implications of changes in DNA methylation, although this is a subject of pivotal importance for the future studies. It should be noted, however, that Archacki et al. [33] identified 29 genes differentially expressed between left anterior descending coronary arteries and internal mammary arteries. Two genes (*F13A1* and *CD163*) were found in common with our list of differentially methylation genes.

A limitation of our study is an inability to attribute the methylation patterns to specific cell types. The methylation patterns identified, consequently, may represent the gene set of methylation effect contributed from the different cells. Therefore differences in DNA methylation can result from changes in the proportions of cell types in atherosclerosis-affected and healthy vascular tissue samples. To assess cell type-specific DNA methylation of complex tissues, laser capture microdissection must be used. DNA yields from such samples, however, are too small to use with the current methods of microarray-based genome-wide DNA methylation analysis. In our approach, since the tissues were taken directly from patients with advance disease, the data can be biased due to possible effects of unrecognized factors and drugs used for the treatment of the patients. Unbiased results for vascular tissues can be achieved by sampling prior to the development of the disease, but this is not feasible in humans. The major strength of our study is the use of quantitative methods for the analysis of DNA methylation at single-base resolution. We also studied matched vascular tissues in order to avoid the effect of interindividual variation of DNA methylation patterns. Chen et al. [34] described a subset of CpG-sites that overlap known SNPs. He proposed that studies that are focused on intraindividual differences rather than interindividual are not expected to be confounded by such underlying SNPs. We also check our two lists of differentially methylated CpG-sites (S1 Table and S3 Table) using the list of polymorphic CpG-sites [34]. There were one CpG-site that overlapped SNPs in the S1 Table (cg10222534) and two CpG-sites in the S3 Table (cg19297688; cg22518733). These CpG-sites were not among the top of our lists.

## Conclusions

We performed a genome-wide DNA methylation profiling study on the right coronary arteries compared with internal mammary arteries and great saphenous veins from the same patients with coronary heart disease. Modest, but consistent and significant, DNA methylation changes in vascular tissues indicate the importance of epigenetic mechanisms in atherosclerosis. We revealed that a high percentage of differentially methylated genes between vascular tissues related to the regulation of the developmental process. This study identified hypomethylation of four CpG-sites located within the *MIR10B* gene sequence and about 1 kb upstream of the *HOXD4* gene in right coronary artery in the area of advanced atherosclerotic plaques in comparison with the other vascular tissues. Further studies need to be carried out to investigate the genes and biological pathways that are involved in the development of atherosclerosis and may serve as targets for prevention and treating vascular diseases.

## Supporting Information

S1 Table Differentially methylated CpG-sites between CAP and IMA groups. FDR-adjusted p<0.05 and delta beta ≥0.2 or delta beta ≤-0.2.
(DOCX)

S2 Table WebGestalt-generated significant ontologies for hypomethylated CpG-sites in CAP. The results for each enriched GO category (biological process) are listed in the table. C—number of reference genes in the category; O—number of genes in the gene set and also in the category, E—expected number in the category, R—Ratio of enrichment, rawP—p value from hypergeometric test, and adjP—p value adjusted by the multiple test adjustment.
(DOCX)

S3 Table Differentially methylated CpG-sites between GSV and IMA groups. FDR-adjusted p<0.05 and delta beta ≥0.2 or delta beta ≤-0.2.
(DOCX)

S4 Table WebGestalt-generated significant ontologies for hypermethylated CpG-sites in GSV. The results for each enriched GO category (biological process) are listed in the table. C—number of reference genes in the category; O—number of genes in the gene set and also in the category, E—expected number in the category, R—Ratio of enrichment, rawP—p value from hypergeometric test, and adjP—p value adjusted by the multiple test adjustment.
(DOCX)
