# Supplementary material for: A Comparison of Genome-Wide DNA Methylation Patterns between Different Vascular Tissues from Patients with Coronary Heart Disease
Source: PLoS One. 2015 Apr 9;10(4):e0122601. doi: 10.1371/journal.pone.0122601 (PMC4391864; doi:10.1371/journal.pone.0122601)
Supplement: S1 Table — FDR-adjusted p<0.05 and delta beta ≥0.2 or delta beta ≤-0.2. (DOCX) [file pone.0122601.s001.docx]

**Differentially methylated CpG-sites between CAP and IMA groups. FDR-adjusted p<0.05 and delta beta ≥0.2 or delta beta ≤-0.2**

| **Probe ID** | **adj.P.Val** | **CAP_beta** | **CAP_beta_SD** | **IMA_beta** | **IMA_beta_SD** | **Average delta beta CAP-IMA** | **GENE_ID** | **SYMBOL** | **DISTANCE_TO_TSS** | **CPG_**  **ISLAND** | **CPG_ISLAND_LOCATIONS** |
| --- | --- | --- | --- | --- | --- | --- | --- | --- | --- | --- | --- |
| cg10925082 | 4.45E-09 | 0.11 | 0.07 | 0.76 | 0.04 | -0.65 | 397 | *ARHGDIB* | 141 | FALSE |  |
| cg01152019 | 1.79E-10 | 0.09 | 0.03 | 0.72 | 0.08 | -0.63 | 3233 | *HOXD4* | 1069 | TRUE | 2:176723073-176723586 |
| cg00767581 | 6.65E-08 | 0.28 | 0.07 | 0.84 | 0.04 | -0.56 | 3233 | *HOXD4* | 988 | TRUE | 2:176723073-176723586 |
| cg12127282 | 2.09E-10 | 0.30 | 0.04 | 0.84 | 0.03 | -0.54 | 3233 | *HOXD4* | 1428 | TRUE | 2:176722497-176722964 |
| cg10795646 | 3.99E-11 | 0.11 | 0.02 | 0.64 | 0.05 | -0.52 | 6281 | *S100A10* | 850 | FALSE |  |
| cg14399060 | 1.79E-10 | 0.08 | 0.02 | 0.57 | 0.07 | -0.48 | 3233 | *HOXD4* | 1043 | TRUE | 2:176723073-176723586 |
| cg19826026 | 1.79E-10 | 0.10 | 0.03 | 0.58 | 0.04 | -0.48 | 397 | *ARHGDIB* | 169 | FALSE |  |
| cg03852144 | 3.84E-09 | 0.09 | 0.04 | 0.57 | 0.11 | -0.48 | 2745 | *GLRX* | 1258 | FALSE |  |
| cg21491308 | 5.62E-09 | 0.10 | 0.03 | 0.56 | 0.07 | -0.46 | 83639 | *TEX101* | 63 | FALSE |  |
| cg21627181 | 4.67E-08 | 0.44 | 0.14 | 0.89 | 0.01 | -0.45 | 10050 | *SLC17A4* | 776 | FALSE |  |
| cg13015534 | 1.61E-11 | 0.20 | 0.03 | 0.62 | 0.03 | -0.43 | 55808 | *ST6GALNAC1* | 60 | FALSE |  |
| cg26620157 | 1.67E-07 | 0.14 | 0.02 | 0.56 | 0.05 | -0.42 | 5083 | *PAX9* | 608 | TRUE | 14:36199815-36200327 |
| cg22088368 | 1.39E-08 | 0.24 | 0.05 | 0.66 | 0.04 | -0.42 | 339669 | *MGC35206* | 1049 | FALSE |  |
| cg26511321 | 9.50E-10 | 0.26 | 0.05 | 0.67 | 0.03 | -0.41 | 3204 | *HOXA7* | 494 | TRUE | 7:27162023-27163571 |
| cg20839149 | 9.23E-08 | 0.13 | 0.06 | 0.54 | 0.03 | -0.41 | 9047 | *SH2D2A* | 1 | TRUE | 1:155053160-155053362 |
| cg00061629 | 1.61E-05 | 0.32 | 0.11 | 0.72 | 0.05 | -0.40 | 60529 | *ALX4* | NA | TRUE | 11:44281312-44281846 |
| cg23737768 | 3.92E-06 | 0.42 | 0.12 | 0.81 | 0.04 | -0.39 | 192666 | *KRT24* | 95 | FALSE |  |
| cg00134539 | 4.37E-07 | 0.52 | 0.14 | 0.90 | 0.03 | -0.38 | 53347 | *UBASH3A* | 52 | FALSE |  |
| cg15916061 | 4.31E-08 | 0.41 | 0.09 | 0.79 | 0.03 | -0.38 | 10050 | *SLC17A4* | 747 | FALSE |  |
| cg17173423 | 4.11E-09 | 0.25 | 0.06 | 0.62 | 0.02 | -0.37 | 932 | *MS4A3* | 108 | FALSE |  |
| cg00509670 | 7.80E-09 | 0.17 | 0.02 | 0.54 | 0.05 | -0.37 | 5083 | *PAX9* | 592 | TRUE | 14:36199815-36200327 |
| cg24841244 | 4.63E-10 | 0.40 | 0.05 | 0.76 | 0.04 | -0.36 | 915 | *CD3D* | 7 | FALSE |  |
| cg22051636 | 0.0027312 | 0.31 | 0.11 | 0.67 | 0.19 | -0.36 | 117194 | *MRGPRX2* | 638 | FALSE |  |
| cg20613889 | 3.46E-05 | 0.36 | 0.14 | 0.72 | 0.04 | -0.35 | 2492 | *FSHR* | 135 | FALSE |  |
| cg04915566 | 0.0002653 | 0.38 | 0.18 | 0.72 | 0.05 | -0.34 | 861 | *RUNX1* | 123 | FALSE |  |
| cg05109049 | 0.0003818 | 0.47 | 0.21 | 0.81 | 0.05 | -0.34 | 2124 | *EVI2B* | 242 | FALSE |  |
| cg00005847 | 3.46E-06 | 0.17 | 0.03 | 0.50 | 0.12 | -0.33 | 3232 | *HOXD3* | 268 | FALSE |  |
| cg19717150 | 7.41E-06 | 0.40 | 0.10 | 0.73 | 0.05 | -0.33 | 3172 | *HNF4A* | 437 | TRUE | 20:42418188-42418589 |
| cg18546622 | 1.95E-06 | 0.21 | 0.06 | 0.54 | 0.05 | -0.33 | 60529 | *ALX4* | NA | TRUE | 11:44281312-44281846 |
| cg23079782 | 9.67E-06 | 0.31 | 0.10 | 0.64 | 0.05 | -0.32 | 27334 | *P2RY10* | 92 | FALSE |  |
| cg26489108 | 1.86E-08 | 0.23 | 0.04 | 0.55 | 0.04 | -0.32 | 58524 | *DMRT3* | 1108 | TRUE | 9:965733-965960 |
| cg16240480 | 0.0026035 | 0.34 | 0.19 | 0.65 | 0.06 | -0.32 | 128178 | *EDARADD* | 207 | FALSE |  |
| cg10771262 | 3.10E-06 | 0.31 | 0.06 | 0.63 | 0.08 | -0.32 | 6943 | *TCF21* | 297 | FALSE |  |
| cg05861567 | 0.0002653 | 0.35 | 0.13 | 0.67 | 0.05 | -0.31 | 23209 | *MLC1* | 228 | TRUE | 22:48865755-48865963 |
| cg20070090 | 3.53E-09 | 0.28 | 0.04 | 0.59 | 0.02 | -0.31 | 6279 | *S100A8* | 60 | FALSE |  |
| cg09432376 | 8.43E-06 | 0.28 | 0.09 | 0.59 | 0.08 | -0.31 | 80830 | *APOL6* | 198 | FALSE |  |
| cg23254045 | 4.61E-06 | 0.37 | 0.05 | 0.68 | 0.08 | -0.31 | 145942 | *TMCO5* | 1003 | FALSE |  |
| cg11161417 | 4.06E-07 | 0.39 | 0.05 | 0.70 | 0.06 | -0.31 | 124912 | *SPACA3* | 572 | FALSE |  |
| cg22628926 | 6.09E-09 | 0.13 | 0.03 | 0.44 | 0.02 | -0.31 | 8402 | *SLC25A11* | 1382 | FALSE |  |
| cg13062935 | 0.0010779 | 0.47 | 0.20 | 0.78 | 0.04 | -0.31 | 117194 | *MRGPRX2* | 286 | FALSE |  |
| cg20261167 | 0.003568 | 0.29 | 0.19 | 0.59 | 0.10 | -0.31 | 6696 | *SPP1* | 140 | FALSE |  |
| cg06434428 | 3.31E-07 | 0.59 | 0.12 | 0.90 | 0.02 | -0.31 | 1404 | *HAPLN1* | 68 | FALSE |  |
| cg27159719 | 0.0037534 | 0.37 | 0.20 | 0.67 | 0.05 | -0.30 | 137835 | *TMEM71* | 86 | FALSE |  |
| cg18878432 | 7.95E-05 | 0.27 | 0.11 | 0.57 | 0.02 | -0.30 | 3216 | *HOXB6* | 1075 | TRUE | 17:44038335-44038975 |
| cg12499211 | 2.63E-05 | 0.35 | 0.11 | 0.65 | 0.05 | -0.30 | 9047 | *SH2D2A* | 41 | TRUE | 1:155053160-155053362 |
| cg23507131 | 2.90E-06 | 0.38 | 0.10 | 0.68 | 0.03 | -0.30 | 2532 | *DARC* | 354 | FALSE |  |
| cg23352695 | 0.0003372 | 0.41 | 0.16 | 0.71 | 0.04 | -0.30 | 2123 | *EVI2A* | 94 | FALSE |  |
| cg11391732 | 2.89E-05 | 0.56 | 0.15 | 0.86 | 0.01 | -0.30 | 8988 | *HSPB3* | 474 | TRUE | 5:53787529-53787747 |
| cg07906724 | 0.0049722 | 0.46 | 0.23 | 0.76 | 0.04 | -0.30 | 8973 | *CHRNA6* | 327 | FALSE |  |
| cg22991148 | 0.00017 | 0.05 | 0.02 | 0.35 | 0.08 | -0.30 | 54084 | *C21orf29* | 396 | TRUE | 21:44955387-44955762 |
| cg21063899 | 3.28E-05 | 0.28 | 0.07 | 0.57 | 0.06 | -0.29 | 8796 | *SCEL* | 58 | FALSE |  |
| cg20789824 | 8.81E-07 | 0.40 | 0.09 | 0.70 | 0.01 | -0.29 | 169611 | *OLFML2A* | 292 | FALSE |  |
| cg04098585 | 0.0079183 | 0.41 | 0.22 | 0.70 | 0.03 | -0.29 | 940 | *CD28* | 17 | FALSE |  |
| cg09893305 | 0.0003948 | 0.42 | 0.13 | 0.71 | 0.09 | -0.29 | 1404 | *HAPLN1* | 486 | FALSE |  |
| cg17251713 | 1.36E-06 | 0.22 | 0.06 | 0.51 | 0.06 | -0.29 | 8710 | *SERPINB7* | 394 | FALSE |  |
| cg21611708 | 0.0002373 | 0.37 | 0.15 | 0.66 | 0.03 | -0.29 | 7053 | *TGM3* | 365 | FALSE |  |
| cg07688234 | 0.0072436 | 0.45 | 0.22 | 0.73 | 0.09 | -0.28 | 5199 | *PFC* | 41 | FALSE |  |
| cg16495265 | 1.61E-11 | 0.06 | 0.01 | 0.34 | 0.03 | -0.28 | 3215 | *HOXB5* | 108 | TRUE | 17:44025432-44027432 |
| cg25426743 | 1.22E-05 | 0.62 | 0.15 | 0.90 | 0.01 | -0.28 | 3200 | *HOXA3* | 536 | FALSE |  |
| cg12869058 | 3.10E-08 | 0.63 | 0.07 | 0.90 | 0.02 | -0.28 | 24 | *ABCA4* | 1193 | FALSE |  |
| cg00795812 | 1.23E-05 | 0.14 | 0.04 | 0.41 | 0.04 | -0.27 | 5133 | *PDCD1* | 951 | TRUE | 2:242450515-242450972 |
| cg11812202 | 8.98E-06 | 0.44 | 0.08 | 0.71 | 0.06 | -0.27 | 5406 | *PNLIP* | 19 | FALSE |  |
| cg05429895 | 3.93E-10 | 0.06 | 0.02 | 0.33 | 0.04 | -0.27 | 7099 | *TLR4* | 56 | FALSE |  |
| cg12941931 | 0.0048842 | 0.43 | 0.21 | 0.70 | 0.03 | -0.27 | 10501 | *SEMA6B* | 1296 | TRUE | 19:4510691-4510902 |
| cg08090640 | 0.004493 | 0.32 | 0.17 | 0.59 | 0.11 | -0.27 | 3430 | *IFI35* | 464 | FALSE |  |
| cg10681065 | 7.13E-07 | 0.42 | 0.06 | 0.69 | 0.03 | -0.27 | 7036 | *TFR2* | 13 | FALSE |  |
| cg17170504 | 2.30E-05 | 0.61 | 0.14 | 0.88 | 0.03 | -0.27 | 29070 | *HSPC065* | 742 | FALSE |  |
| cg24292612 | 2.35E-08 | 0.39 | 0.04 | 0.66 | 0.04 | -0.27 | 1672 | *DEFB1* | 57 | FALSE |  |
| cg25965576 | 2.76E-06 | 0.38 | 0.08 | 0.65 | 0.03 | -0.27 | 1472 | *CST4* | 427 | FALSE |  |
| cg11325578 | 4.51E-06 | 0.14 | 0.03 | 0.41 | 0.09 | -0.27 | 4935 | *GPR143* | 630 | TRUE | X:9692673-9694068 |
| cg14437986 | 4.88E-07 | 0.36 | 0.06 | 0.63 | 0.03 | -0.27 | 80739 | *C6orf25* | 126 | TRUE | 6:31798937-31799213 |
| cg22325572 | 0.0012075 | 0.37 | 0.16 | 0.64 | 0.03 | -0.27 | 963 | *CD53* | 408 | FALSE |  |
| cg15551881 | 0.000643 | 0.39 | 0.12 | 0.65 | 0.06 | -0.27 | 7185 | *TRAF1* | 458 | FALSE |  |
| cg17327492 | 6.55E-06 | 0.52 | 0.10 | 0.78 | 0.02 | -0.26 | 2492 | *FSHR* | 529 | FALSE |  |
| cg15133917 | 8.76E-07 | 0.40 | 0.07 | 0.67 | 0.02 | -0.26 | 7273 | *TTN* | 488 | FALSE |  |
| cg15700739 | 0.0028522 | 0.17 | 0.06 | 0.43 | 0.13 | -0.26 | 3222 | *HOXC5* | 868 | TRUE | 12:52712802-52715131 |
| cg26385286 | 0.0001703 | 0.32 | 0.10 | 0.57 | 0.06 | -0.26 | 2651 | *GCNT2* | 774 | FALSE |  |
| cg08477744 | 1.34E-06 | 0.60 | 0.04 | 0.85 | 0.06 | -0.26 | 4237 | *MFAP2* | 49 | FALSE |  |
| cg19358442 | 1.12E-06 | 0.24 | 0.05 | 0.50 | 0.03 | -0.26 | 60529 | *ALX4* | NA | TRUE | 11:44282161-44283221 |
| cg00476577 | 7.23E-06 | 0.09 | 0.06 | 0.35 | 0.06 | -0.26 | 7764 | *ZNF217* | 772 | FALSE |  |
| cg09059945 | 2.50E-06 | 0.15 | 0.05 | 0.41 | 0.06 | -0.26 | 4232 | *MEST* | 28 | TRUE | 7:129912883-129914215 |
| cg16890093 | 0.0170769 | 0.55 | 0.26 | 0.80 | 0.04 | -0.26 | 5696 | *PSMB8* | 372 | FALSE |  |
| cg24898863 | 7.98E-08 | 0.15 | 0.03 | 0.40 | 0.04 | -0.26 | 6279 | *S100A8* | 31 | FALSE |  |
| cg20678353 | 0.0063126 | 0.32 | 0.16 | 0.58 | 0.06 | -0.25 | 400359 | *FLJ35695* | 239 | FALSE |  |
| cg03440267 | 8.49E-06 | 0.20 | 0.07 | 0.45 | 0.04 | -0.25 | 2162 | *F13A1* | 159 | FALSE |  |
| cg10150530 | 4.49E-07 | 0.11 | 0.04 | 0.36 | 0.06 | -0.25 | 4033 | *LRMP* | 227 | FALSE |  |
| cg09872616 | 3.10E-07 | 0.16 | 0.04 | 0.41 | 0.06 | -0.25 | 4232 | *MEST* | 136 | TRUE | 7:129912883-129914215 |
| cg14646244 | 5.91E-06 | 0.32 | 0.09 | 0.57 | 0.03 | -0.25 | 5172 | *SLC26A4* | 793 | FALSE |  |
| cg27446185 | 1.57E-06 | 0.43 | 0.06 | 0.68 | 0.05 | -0.25 | 79679 | *VTCN1* | 418 | FALSE |  |
| cg10636246 | 0.0350093 | 0.42 | 0.26 | 0.67 | 0.10 | -0.25 | 9447 | *AIM2* | 326 | FALSE |  |
| cg18403361 | 0.0111955 | 0.19 | 0.10 | 0.44 | 0.14 | -0.24 | 161198 | *CLEC14A* | 176 | TRUE | 14:37793841-37795561 |
| cg12207371 | 2.29E-05 | 0.47 | 0.07 | 0.72 | 0.04 | -0.24 | 9332 | *CD163* | 569 | FALSE |  |
| cg02939139 | 0.0001371 | 0.22 | 0.09 | 0.46 | 0.08 | -0.24 | 8437 | *RASAL1* | 183 | TRUE | 12:112058279-112058595 |
| cg05125838 | 2.61E-06 | 0.36 | 0.06 | 0.60 | 0.04 | -0.24 | 90226 | *UCN2* | 384 | FALSE |  |
| cg22672790 | 1.01E-05 | 0.23 | 0.03 | 0.48 | 0.05 | -0.24 | 2559 | *GABRA6* | 183 | FALSE |  |
| cg01138020 | 0.0004996 | 0.40 | 0.13 | 0.65 | 0.04 | -0.24 | 201305 | *MGC29671* | 125 | FALSE |  |
| cg09172980 | 4.25E-07 | 0.14 | 0.03 | 0.38 | 0.03 | -0.24 | 9027 | *NAT8* | 647 | FALSE |  |
| cg20804821 | 0.0029059 | 0.42 | 0.15 | 0.66 | 0.09 | -0.24 | 118442 | *GPR62* | 943 | FALSE |  |
| cg05607401 | 0.0001499 | 0.28 | 0.10 | 0.53 | 0.02 | -0.24 | 9535 | *GMFG* | 73 | FALSE |  |
| cg20090497 | 1.92E-06 | 0.51 | 0.08 | 0.75 | 0.03 | -0.24 | 50835 | *TAS2R9* | 330 | FALSE |  |
| cg03294557 | 1.49E-06 | 0.26 | 0.05 | 0.50 | 0.03 | -0.24 | 6318 | *SERPINB4* | 987 | FALSE |  |
| cg18750960 | 1.63E-06 | 0.10 | 0.02 | 0.34 | 0.08 | -0.24 | 3233 | *HOXD4* | 304 | TRUE | 2:176724528-176725909 |
| cg03301801 | 0.0008939 | 0.57 | 0.16 | 0.81 | 0.03 | -0.24 | 163479 | *FNDC7* | 750 | FALSE |  |
| cg23865698 | 0.0001778 | 0.29 | 0.09 | 0.53 | 0.04 | -0.24 | 58189 | *WFDC1* | 593 | FALSE |  |
| cg05439368 | 0.001151 | 0.35 | 0.14 | 0.59 | 0.06 | -0.24 | 140691 | *RNF36* | 624 | FALSE |  |
| cg19862344 | 0.0374367 | 0.32 | 0.23 | 0.55 | 0.09 | -0.24 | 6005 | *RHAG* | 629 | FALSE |  |
| cg03634982 | 0.0204023 | 0.58 | 0.24 | 0.82 | 0.03 | -0.24 | 51338 | *MS4A4A* | 160 | FALSE |  |
| cg09937039 | 0.0009145 | 0.22 | 0.11 | 0.45 | 0.02 | -0.24 | 10538 | *BATF* | 419 | FALSE |  |
| cg21932814 | 1.67E-07 | 0.28 | 0.03 | 0.51 | 0.03 | -0.24 | 1475 | *CSTA* | 161 | FALSE |  |
| cg20385229 | 1.69E-06 | 0.54 | 0.06 | 0.77 | 0.03 | -0.23 | 8846 | *ALKBH* | 1126 | FALSE |  |
| cg10161121 | 1.63E-09 | 0.56 | 0.03 | 0.79 | 0.03 | -0.23 | 356 | *FASLG* | 165 | FALSE |  |
| cg11854007 | 1.86E-06 | 0.10 | 0.03 | 0.33 | 0.08 | -0.23 | 317716 | *RP11-49G10.8* | 286 | FALSE |  |
| cg02131853 | 0.0003243 | 0.54 | 0.14 | 0.77 | 0.02 | -0.23 | 80008 | *FLJ23235* | 596 | FALSE |  |
| cg20283107 | 1.22E-08 | 0.20 | 0.02 | 0.43 | 0.04 | -0.23 | 157769 | *FAM91A1* | 1358 | FALSE |  |
| cg02947253 | 3.40E-07 | 0.65 | 0.04 | 0.88 | 0.02 | -0.23 | 140 | *ADORA3* | 43 | FALSE |  |
| cg02224164 | 6.43E-05 | 0.46 | 0.09 | 0.69 | 0.04 | -0.22 | 64333 | *ARHGAP9* | 718 | FALSE |  |
| cg03548857 | 0.0001383 | 0.49 | 0.10 | 0.72 | 0.02 | -0.22 | 2867 | *FFAR2* | 443 | FALSE |  |
| cg26789453 | 0.0221394 | 0.58 | 0.24 | 0.80 | 0.04 | -0.22 | 89894 | *TMEM116* | 1489 | FALSE |  |
| cg11481351 | 0.0011442 | 0.55 | 0.14 | 0.77 | 0.04 | -0.22 | 163479 | *FNDC7* | 354 | FALSE |  |
| cg16692277 | 0.0001618 | 0.26 | 0.09 | 0.48 | 0.06 | -0.22 | 2974 | *GUCY1B2* | 806 | TRUE | 13:50538899-50539111 |
| cg26832879 | 3.16E-08 | 0.67 | 0.06 | 0.89 | 0.01 | -0.22 | 29844 | *TFPT* | 1037 | FALSE |  |
| cg15361750 | 2.77E-06 | 0.32 | 0.06 | 0.54 | 0.02 | -0.22 | 27202 | *GPR77* | 1239 | FALSE |  |
| cg04111761 | 8.24E-07 | 0.44 | 0.05 | 0.67 | 0.04 | -0.22 | 1232 | *CCR3* | 922 | FALSE |  |
| cg13618372 | 4.19E-05 | 0.57 | 0.10 | 0.79 | 0.02 | -0.22 | 6711 | *SPTBN1* | 116 | FALSE |  |
| cg10451565 | 0.0008626 | 0.55 | 0.13 | 0.77 | 0.02 | -0.22 | 27202 | *GPR77* | 240 | FALSE |  |
| cg21109025 | 2.51E-05 | 0.59 | 0.09 | 0.81 | 0.03 | -0.22 | 6347 | *CCL2* | 1427 | FALSE |  |
| cg17199483 | 0.0296001 | 0.37 | 0.22 | 0.58 | 0.02 | -0.22 | 4363 | *ABCC1* | 667 | TRUE | 16:15950264-15951523 |
| cg23216015 | 0.0034948 | 0.31 | 0.11 | 0.52 | 0.09 | -0.22 | 10842 | *C7orf16* | 193 | FALSE |  |
| cg06378617 | 6.98E-05 | 0.48 | 0.06 | 0.69 | 0.05 | -0.21 | 25984 | *KRT23* | 1305 | FALSE |  |
| cg03907363 | 4.81E-05 | 0.41 | 0.06 | 0.62 | 0.05 | -0.21 | 3816 | *KLK1* | 361 | FALSE |  |
| cg11804789 | 0.0114998 | 0.46 | 0.19 | 0.68 | 0.02 | -0.21 | 8530 | *CST7* | 150 | FALSE |  |
| cg16303562 | 0.002829 | 0.47 | 0.14 | 0.68 | 0.03 | -0.21 | 4481 | *MSR1* | 109 | FALSE |  |
| cg26233914 | 6.79E-06 | 0.10 | 0.04 | 0.31 | 0.05 | -0.21 | 3687 | *ITGAX* | 27 | FALSE |  |
| cg06771126 | 0.0023347 | 0.14 | 0.03 | 0.35 | 0.13 | -0.21 | 84525 | *HOP* | 134 | FALSE |  |
| cg07525077 | 0.0075904 | 0.53 | 0.14 | 0.74 | 0.04 | -0.21 | 6037 | *RNASE3* | 381 | FALSE |  |
| cg01888566 | 3.08E-05 | 0.27 | 0.07 | 0.48 | 0.04 | -0.21 | 4232 | *MEST* | NA | TRUE | 7:129912883-129914215 |
| cg14826683 | 1.12E-05 | 0.56 | 0.06 | 0.77 | 0.03 | -0.21 | 6703 | *SPRR2D* | 574 | FALSE |  |
| cg22960952 | 7.54E-05 | 0.35 | 0.07 | 0.56 | 0.05 | -0.21 | 152816 | *FLJ23657* | 89 | FALSE |  |
| cg21406461 | 0.0012779 | 0.31 | 0.12 | 0.52 | 0.07 | -0.21 | 3428 | *IFI16* | 751 | FALSE |  |
| cg13578652 | 1.88E-05 | 0.36 | 0.05 | 0.57 | 0.04 | -0.21 | 53347 | *UBASH3A* | 156 | FALSE |  |
| cg06130787 | 1.63E-09 | 0.08 | 0.02 | 0.29 | 0.02 | -0.21 | 5655 | *KLK10* | 268 | FALSE |  |
| cg03179866 | 3.46E-06 | 0.46 | 0.04 | 0.67 | 0.04 | -0.21 | 4321 | *MMP12* | 922 | FALSE |  |
| cg08404225 | 0.0068153 | 0.19 | 0.11 | 0.40 | 0.06 | -0.21 | 3568 | *IL5RA* | 132 | FALSE |  |
| cg22010317 | 0.0010634 | 0.55 | 0.11 | 0.76 | 0.06 | -0.21 | 79742 | *CXorf36* | 398 | FALSE |  |
| cg20022122 | 5.98E-05 | 0.55 | 0.08 | 0.76 | 0.05 | -0.21 | 2646 | *GCKR* | 416 | FALSE |  |
| cg14982472 | 0.0006718 | 0.19 | 0.07 | 0.40 | 0.05 | -0.21 | 9619 | *ABCG1* | 148 | FALSE |  |
| cg12966875 | 0.0093603 | 0.24 | 0.15 | 0.45 | 0.05 | -0.21 | 6590 | *SLPI* | 540 | FALSE |  |
| cg01997953 | 0.0024153 | 0.51 | 0.14 | 0.72 | 0.02 | -0.21 | 80231 | *CXorf21* | 224 | FALSE |  |
| cg17936488 | 0.0140591 | 0.49 | 0.19 | 0.70 | 0.04 | -0.21 | 286336 | *FAM78A* | 387 | TRUE | 9:133141048-133141374 |
| cg10045881 | 0.0016957 | 0.51 | 0.10 | 0.71 | 0.08 | -0.20 | 1117 | *CHI3L2* | 10 | FALSE |  |
| cg15046675 | 0.0335368 | 0.68 | 0.24 | 0.88 | 0.02 | -0.20 | 951 | *CD37* | 42 | FALSE |  |
| cg08077673 | 6.72E-05 | 0.21 | 0.05 | 0.41 | 0.07 | -0.20 | 4232 | *MEST* | 322 | TRUE | 7:129912883-129914215 |
| cg18216249 | 0.0163095 | 0.36 | 0.18 | 0.57 | 0.08 | -0.20 | 79657 | *FLJ21908* | 1031 | FALSE |  |
| cg23447996 | 1.07E-05 | 0.27 | 0.05 | 0.47 | 0.05 | -0.20 | 6850 | *SYK* | 533 | TRUE | 9:92603527-92604494 |
| cg22510822 | 0.0001119 | 0.66 | 0.11 | 0.86 | 0.01 | -0.20 | 8388 | *OR1E2* | 292 | FALSE |  |
| cg27631256 | 0.0455457 | 0.60 | 0.24 | 0.80 | 0.02 | -0.20 | 944 | *TNFSF8* | 25 | TRUE | 9:116732561-116732855 |
| cg19006008 | 2.24E-12 | 0.07 | 0.00 | 0.27 | 0.02 | -0.20 | 9002 | *F2RL3* | 58 | FALSE |  |
| cg27067621 | 2.14E-05 | 0.31 | 0.06 | 0.51 | 0.05 | -0.20 | 27035 | *NOX1* | 234 | FALSE |  |
| cg20017147 | 1.36E-06 | 0.39 | 0.05 | 0.59 | 0.03 | -0.20 | 83639 | *TEX101* | 161 | FALSE |  |
| cg26135325 | 0.0003692 | 0.53 | 0.11 | 0.73 | 0.04 | -0.20 | 353142 | *LCE3A* | 257 | FALSE |  |
| cg24030609 | 0.0024529 | 0.46 | 0.10 | 0.67 | 0.08 | -0.20 | 51032 | *ELA2B* | 15 | FALSE |  |
| cg19662708 | 3.13E-06 | 0.53 | 0.05 | 0.73 | 0.03 | -0.20 | 7003 | *TEAD1* | 152 | FALSE |  |
| cg26151675 | 0.0338726 | 0.41 | 0.21 | 0.61 | 0.07 | -0.20 | 1269 | *CNR2* | 252 | FALSE |  |
| cg17607231 | 0.0069119 | 0.58 | 0.17 | 0.78 | 0.04 | -0.20 | 11262 | *SP140* | 117 | FALSE |  |
| cg16478792 | 7.50E-07 | 0.83 | 0.03 | 0.63 | 0.05 | 0.20 | 353299 | *RGSL1* | 844 | FALSE |  |
| cg05835416 | 7.98E-08 | 0.65 | 0.04 | 0.45 | 0.01 | 0.20 | 1562 | *CYP2C18* | 704 | FALSE |  |
| cg23671708 | 0.0037999 | 0.46 | 0.15 | 0.26 | 0.05 | 0.20 | 92162 | *TMEM88* | 93 | TRUE | 17:7698629-7699147 |
| cg04096767 | 2.50E-05 | 0.50 | 0.05 | 0.30 | 0.03 | 0.20 | 7490 | *WT1* | NA | TRUE | 11:32404590-32406255 |
| cg23114594 | 6.13E-05 | 0.81 | 0.07 | 0.61 | 0.02 | 0.20 | 222389 | *C10orf30* | 328 | FALSE |  |
| cg22858288 | 0.0002939 | 0.58 | 0.09 | 0.38 | 0.06 | 0.20 | 5002 | *SLC22A18* | NA | TRUE | 11:2886834-2887801 |
| cg13594711 | 0.0002251 | 0.82 | 0.07 | 0.62 | 0.03 | 0.20 | 136853 | *SRCRB4D* | 227 | FALSE |  |
| cg15701111 | 0.0001305 | 0.64 | 0.06 | 0.44 | 0.04 | 0.20 | 3887 | *KRTHB1* | 35 | TRUE | 12:50971078-50971650 |
| cg12754854 | 0.0238009 | 0.41 | 0.21 | 0.20 | 0.04 | 0.20 | 254295 | *PHYHD1* | 828 | FALSE |  |
| cg07922606 | 0.0013084 | 0.55 | 0.11 | 0.35 | 0.05 | 0.20 | 8353 | *HIST1H3E* | 6 | TRUE | 6:26333255-26333868 |
| cg25608041 | 0.0064869 | 0.38 | 0.16 | 0.17 | 0.05 | 0.20 | 23216 | *TBC1D1* | 886 | TRUE | 4:37567883-37569855 |
| cg24781100 | 0.0003594 | 0.49 | 0.10 | 0.29 | 0.04 | 0.20 | 4604 | *MYBPC1* | 648 | FALSE |  |
| cg21409833 | 0.0004989 | 0.82 | 0.05 | 0.62 | 0.11 | 0.20 | 27329 | *ANGPTL3* | 463 | FALSE |  |
| cg02192520 | 0.0204682 | 0.28 | 0.23 | 0.07 | 0.01 | 0.20 | 5959 | *RDH5* | 66 | FALSE |  |
| cg06531741 | 0.0016447 | 0.49 | 0.12 | 0.29 | 0.07 | 0.20 | 9177 | *HTR3B* | 139 | FALSE |  |
| cg03662459 | 0.0001986 | 0.47 | 0.10 | 0.27 | 0.05 | 0.20 | 3590 | *IL11RA* | 358 | FALSE |  |
| cg19257550 | 0.0075489 | 0.47 | 0.17 | 0.26 | 0.07 | 0.20 | 768 | *CA9* | 6 | FALSE |  |
| cg25370441 | 0.000236 | 0.50 | 0.09 | 0.30 | 0.06 | 0.20 | 54848 | *FLJ20184* | 544 | FALSE |  |
| cg04755662 | 0.0155911 | 0.31 | 0.22 | 0.11 | 0.03 | 0.20 | 367 | *AR* | 515 | FALSE |  |
| cg03336167 | 0.0404283 | 0.42 | 0.22 | 0.22 | 0.08 | 0.20 | 5002 | *SLC22A18* | NA | TRUE | 11:2886834-2887801 |
| cg09712066 | 0.024798 | 0.55 | 0.17 | 0.35 | 0.12 | 0.20 | 25859 | *PART1* | 130 | FALSE |  |
| cg02298612 | 0.0028521 | 0.53 | 0.14 | 0.33 | 0.05 | 0.20 | 4166 | *CHST6* | 467 | TRUE | 16:74085934-74087021 |
| cg20802392 | 2.53E-05 | 0.30 | 0.11 | 0.10 | 0.02 | 0.20 | 1513 | *CTSK* | 128 | TRUE | 1:149047294-149047502 |
| cg22545356 | 0.0002328 | 0.44 | 0.11 | 0.23 | 0.04 | 0.21 | 79812 | *MMRN2* | 713 | FALSE |  |
| cg22670733 | 0.000125 | 0.79 | 0.06 | 0.58 | 0.05 | 0.21 | 1136 | *CHRNA3* | 962 | TRUE | 15:76701258-76701471 |
| cg09243900 | 5.90E-05 | 0.71 | 0.08 | 0.50 | 0.04 | 0.21 | 57111 | *RAB25* | 138 | FALSE |  |
| cg25488547 | 0.0077391 | 0.68 | 0.12 | 0.47 | 0.10 | 0.21 | 1121 | *CHM* | 743 | FALSE |  |
| cg20831708 | 0.0330308 | 0.37 | 0.17 | 0.16 | 0.11 | 0.21 | 25956 | *SEC31L2* | 222 | TRUE | 10:102268974-102269882 |
| cg24833277 | 4.53E-05 | 0.84 | 0.04 | 0.63 | 0.07 | 0.21 | 84985 | *FAM83A* | 391 | TRUE | 8:124263343-124263618 |
| cg03604278 | 2.36E-06 | 0.45 | 0.05 | 0.24 | 0.03 | 0.21 | 63924 | *CIDEC* | 1494 | FALSE |  |
| cg18752854 | 0.0001181 | 0.56 | 0.04 | 0.35 | 0.07 | 0.21 | 7145 | *TNS1* | 493 | FALSE |  |
| cg26970800 | 7.26E-05 | 0.73 | 0.07 | 0.52 | 0.05 | 0.21 | 2694 | *GIF* | 1238 | FALSE |  |
| cg15791248 | 1.22E-05 | 0.58 | 0.05 | 0.37 | 0.03 | 0.21 | 2260 | *FGFR1* | 594 | FALSE |  |
| cg20804101 | 0.0001545 | 0.38 | 0.11 | 0.17 | 0.03 | 0.21 | 6540 | *SLC6A13* | 86 | FALSE |  |
| cg21717724 | 0.022415 | 0.55 | 0.19 | 0.34 | 0.09 | 0.21 | 5711 | *PSMD5* | 692 | TRUE | 9:122644137-122644399 |
| cg17063201 | 1.22E-08 | 0.89 | 0.02 | 0.68 | 0.04 | 0.21 | 3062 | *HCRTR2* | 507 | FALSE |  |
| cg07654896 | 0.0146583 | 0.37 | 0.20 | 0.17 | 0.04 | 0.21 | 9104 | *RGN* | 54 | TRUE | X:46822380-46823326 |
| cg23173910 | 0.0003602 | 0.55 | 0.10 | 0.34 | 0.05 | 0.21 | 72 | *ACTG2* | 181 | FALSE |  |
| cg21870884 | 0.0007484 | 0.33 | 0.11 | 0.12 | 0.06 | 0.21 | 2848 | *GPR25* | 263 | TRUE | 1:199108740-199110158 |
| cg19601328 | 0.0114987 | 0.65 | 0.09 | 0.44 | 0.11 | 0.21 | 9020 | *MAP3K14* | 387 | FALSE |  |
| cg02382666 | 0.001353 | 0.67 | 0.06 | 0.46 | 0.12 | 0.21 | 800 | *CALD1* | 717 | FALSE |  |
| cg06241300 | 0.0006164 | 0.71 | 0.10 | 0.50 | 0.03 | 0.21 | 55937 | *APOM* | 402 | FALSE |  |
| cg03547924 | 0.0188259 | 0.30 | 0.22 | 0.09 | 0.02 | 0.21 | 8200 | *GDF5* | 100 | FALSE |  |
| cg13781408 | 0.0018341 | 0.80 | 0.08 | 0.59 | 0.09 | 0.21 | 9625 | *AATK* | 401 | TRUE | 17:76710126-76711293 |
| cg04559909 | 7.80E-06 | 0.77 | 0.05 | 0.56 | 0.05 | 0.21 | 6374 | *CXCL5* | 309 | FALSE |  |
| cg27244482 | 8.09E-05 | 0.90 | 0.02 | 0.69 | 0.11 | 0.21 | 815 | *CAMK2A* | 152 | TRUE | 5:149649421-149649720 |
| cg19172575 | 2.86E-07 | 0.39 | 0.04 | 0.18 | 0.01 | 0.21 | 2626 | *GATA4* | NA | TRUE | 8:11605055-11605961 |
| cg00532335 | 1.86E-06 | 0.53 | 0.04 | 0.32 | 0.02 | 0.21 | 9211 | *LGI1* | 817 | FALSE |  |
| cg06339706 | 0.0051328 | 0.46 | 0.16 | 0.25 | 0.05 | 0.21 | 57664 | *PLEKHA4* | 573 | FALSE |  |
| cg04616566 | 0.0086185 | 0.38 | 0.15 | 0.16 | 0.09 | 0.21 | 79875 | *THSD4* | 328 | FALSE |  |
| cg12113132 | 0.0020608 | 0.52 | 0.15 | 0.30 | 0.02 | 0.21 | 23582 | *CCNDBP1* | 617 | FALSE |  |
| cg27600794 | 1.30E-05 | 0.88 | 0.04 | 0.67 | 0.08 | 0.21 | 5407 | *PNLIPRP1* | 1060 | FALSE |  |
| cg02228185 | 9.84E-07 | 0.39 | 0.05 | 0.18 | 0.03 | 0.21 | 443 | *ASPA* | 271 | FALSE |  |
| cg21836062 | 0.0071328 | 0.33 | 0.19 | 0.12 | 0.02 | 0.21 | 1756 | *DMD* | 65 | FALSE |  |
| cg05654164 | 0.0148413 | 0.32 | 0.21 | 0.10 | 0.03 | 0.22 | 148423 | *C1orf52* | 567 | FALSE |  |
| cg13439730 | 0.0012263 | 0.61 | 0.13 | 0.40 | 0.03 | 0.22 | 5652 | *PRSS8* | 152 | TRUE | 16:31054357-31054558 |
| cg10537079 | 4.31E-08 | 0.83 | 0.03 | 0.61 | 0.05 | 0.22 | 5009 | *OTC* | 297 | FALSE |  |
| cg04123507 | 0.0014485 | 0.67 | 0.11 | 0.45 | 0.05 | 0.22 | 3892 | *KRTHB6* | 379 | TRUE | 12:50981438-50982407 |
| cg11170796 | 1.83E-05 | 0.62 | 0.07 | 0.41 | 0.04 | 0.22 | 6929 | *TCF3* | 53 | FALSE |  |
| cg27409364 | 6.56E-05 | 0.34 | 0.08 | 0.12 | 0.05 | 0.22 | 3746 | *KCNC1* | 203 | TRUE | 11:17712319-17714962 |
| cg06417962 | 0.003623 | 0.59 | 0.16 | 0.37 | 0.05 | 0.22 | 6050 | *RNH1* | 697 | TRUE | 11:495635-498135 |
| cg25902889 | 0.0115811 | 0.35 | 0.22 | 0.13 | 0.02 | 0.22 | 79187 | *FSD1* | 399 | FALSE |  |
| cg22449114 | 1.75E-06 | 0.34 | 0.03 | 0.12 | 0.03 | 0.22 | 6939 | *TCF15* | 667 | TRUE | 20:538102-539417 |
| cg00295325 | 1.44E-05 | 0.81 | 0.04 | 0.59 | 0.06 | 0.22 | 8647 | *ABCB11* | 99 | FALSE |  |
| cg10851775 | 2.64E-06 | 0.77 | 0.03 | 0.55 | 0.06 | 0.22 | 115111 | *SLC26A7* | 1044 | FALSE |  |
| cg26218269 | 0.003158 | 0.61 | 0.13 | 0.39 | 0.05 | 0.22 | 10586 | *MAB21L2* | 76 | TRUE | 4:151722468-151723167 |
| cg13760253 | 3.29E-07 | 0.76 | 0.04 | 0.54 | 0.03 | 0.22 | 85479 | *DNAJC5B* | 1095 | FALSE |  |
| cg06714705 | 0.0140415 | 0.38 | 0.21 | 0.15 | 0.03 | 0.22 | 283298 | *OLFML1* | 185 | FALSE |  |
| cg13903548 | 1.11E-05 | 0.65 | 0.07 | 0.42 | 0.03 | 0.22 | 2570 | *GABRR2* | 406 | FALSE |  |
| cg20035459 | 0.0022971 | 0.58 | 0.10 | 0.36 | 0.05 | 0.22 | 9074 | *CLDN6* | 545 | TRUE | 16:3007867-3009478 |
| cg26394380 | 1.22E-08 | 0.76 | 0.03 | 0.53 | 0.01 | 0.22 | 6439 | *SFTPB* | 689 | FALSE |  |
| cg18881723 | 0.0002241 | 0.38 | 0.13 | 0.16 | 0.02 | 0.22 | 6504 | *SLAMF1* | 1 | FALSE |  |
| cg00594952 | 0.0028982 | 0.38 | 0.18 | 0.16 | 0.04 | 0.22 | 9783 | *RIMS3* | 687 | FALSE |  |
| cg15475323 | 0.0135433 | 0.33 | 0.23 | 0.11 | 0.03 | 0.22 | 284358 | *FLJ36070* | 112 | FALSE |  |
| cg02519218 | 0.0011273 | 0.56 | 0.13 | 0.34 | 0.05 | 0.22 | 55743 | *CHFR* | NA | TRUE | 12:131934293-131934917 |
| cg20191453 | 3.90E-05 | 0.48 | 0.04 | 0.25 | 0.07 | 0.22 | 275 | *AMT* | 157 | TRUE | 3:49434750-49435011 |
| cg04737046 | 0.0013327 | 0.63 | 0.12 | 0.40 | 0.04 | 0.23 | 5244 | *ABCB4* | 378 | FALSE |  |
| cg19404979 | 0.0002912 | 0.47 | 0.05 | 0.24 | 0.05 | 0.23 | 64100 | *ELSPBP1* | 100 | FALSE |  |
| cg02274362 | 0.0004651 | 0.68 | 0.10 | 0.45 | 0.05 | 0.23 | 84968 | *PNMA6A* | 632 | TRUE | X:151990675-151993000 |
| cg19109431 | 2.39E-05 | 0.77 | 0.03 | 0.55 | 0.10 | 0.23 | 4794 | *NFKBIE* | 986 | FALSE |  |
| cg10238171 | 4.87E-07 | 0.48 | 0.03 | 0.26 | 0.03 | 0.23 | 623 | *BDKRB1* | 393 | FALSE |  |
| cg00430287 | 0.0361264 | 0.50 | 0.24 | 0.27 | 0.11 | 0.23 | 2444 | *FRK* | 955 | FALSE |  |
| cg09625066 | 0.0137953 | 0.45 | 0.21 | 0.22 | 0.06 | 0.23 | 9104 | *RGN* | 204 | TRUE | X:46822380-46823326 |
| cg17347253 | 1.28E-06 | 0.57 | 0.06 | 0.34 | 0.04 | 0.23 | 4232 | *MEST* | NA | TRUE | 7:129917351-129920395 |
| cg26847490 | 1.15E-05 | 0.53 | 0.07 | 0.29 | 0.03 | 0.23 | 51666 | *ASB4* | 5 | FALSE |  |
| cg07926025 | 2.11E-06 | 0.81 | 0.03 | 0.58 | 0.07 | 0.23 | 9350 | *CER1* | 149 | FALSE |  |
| cg24194775 | 0.0232419 | 0.35 | 0.24 | 0.12 | 0.08 | 0.23 | 4882 | *NPR2* | 931 | TRUE | 9:35781291-35782084 |
| cg06536578 | 0.0001568 | 0.68 | 0.10 | 0.44 | 0.03 | 0.24 | 84502 | *JPH4* | 561 | FALSE |  |
| cg09626984 | 6.38E-07 | 0.31 | 0.07 | 0.07 | 0.03 | 0.24 | 2626 | *GATA4* | NA | TRUE | 8:11602473-11604934 |
| cg00846036 | 9.83E-07 | 0.41 | 0.06 | 0.17 | 0.03 | 0.24 | 1740 | *DLG2* | 312 | FALSE |  |
| cg09440340 | 1.49E-06 | 0.42 | 0.06 | 0.18 | 0.02 | 0.24 | 126868 | *C1orf161* | 39 | FALSE |  |
| cg11554605 | 4.14E-08 | 0.79 | 0.03 | 0.55 | 0.03 | 0.24 | 51666 | *ASB4* | 70 | FALSE |  |
| cg13099330 | 0.0028982 | 0.63 | 0.10 | 0.38 | 0.11 | 0.24 | 5947 | *RBP1* | 691 | TRUE | 3:140740275-140741996 |
| cg13109289 | 3.08E-09 | 0.86 | 0.02 | 0.62 | 0.04 | 0.24 | 56913 | *C1GALT1* | 893 | FALSE |  |
| cg23131950 | 2.58E-05 | 0.82 | 0.04 | 0.57 | 0.08 | 0.24 | 1175 | *AP2S1* | 427 | TRUE | 19:52046359-52046702 |
| cg17253459 | 1.30E-05 | 0.69 | 0.08 | 0.44 | 0.02 | 0.24 | 56944 | *OLFML3* | 640 | FALSE |  |
| cg09558502 | 1.46E-06 | 0.33 | 0.10 | 0.09 | 0.01 | 0.24 | 5016 | *OVGP1* | 132 | FALSE |  |
| cg08946332 | 0.0003344 | 0.79 | 0.08 | 0.54 | 0.07 | 0.24 | 239 | *ALOX12* | 484 | TRUE | 17:6839463-6841283 |
| cg26391080 | 0.0064871 | 0.63 | 0.17 | 0.38 | 0.07 | 0.24 | 387694 | *SH2D4B* | 261 | FALSE |  |
| cg19594666 | 0.0001339 | 0.40 | 0.14 | 0.16 | 0.04 | 0.24 | 3952 | *LEP* | 51 | TRUE | 7:127667928-127668724 |
| cg23910243 | 0.0006019 | 0.46 | 0.15 | 0.21 | 0.05 | 0.24 | 7041 | *TGFB1I1* | 92 | FALSE |  |
| cg04457051 | 1.40E-05 | 0.84 | 0.05 | 0.60 | 0.07 | 0.25 | 60592 | *SCOC* | 29 | FALSE |  |
| cg09548084 | 4.33E-05 | 0.62 | 0.07 | 0.38 | 0.05 | 0.25 | 51000 | *SLC35B3* | 593 | TRUE | 6:8380156-8381258 |
| cg05209917 | 0.000314 | 0.56 | 0.11 | 0.32 | 0.04 | 0.25 | 27255 | *CNTN6* | 101 | FALSE |  |
| cg14283939 | 3.42E-06 | 0.91 | 0.02 | 0.67 | 0.12 | 0.25 | 79875 | *THSD4* | 20 | TRUE | 15:69807869-69808151 |
| cg23400451 | 3.70E-05 | 0.81 | 0.05 | 0.56 | 0.06 | 0.25 | 4622 | *MYH4* | 852 | FALSE |  |
| cg05982504 | 2.59E-07 | 0.77 | 0.05 | 0.53 | 0.03 | 0.25 | 3483 | *IGFALS* | 1253 | TRUE | 16:1784796-1785119 |
| cg11946165 | 2.91E-07 | 0.66 | 0.04 | 0.41 | 0.03 | 0.25 | 1513 | *CTSK* | 622 | FALSE |  |
| cg09414535 | 0.0077391 | 0.48 | 0.21 | 0.23 | 0.04 | 0.25 | 23426 | *GRIP1* | 103 | FALSE |  |
| cg25119415 | 5.57E-06 | 0.84 | 0.04 | 0.59 | 0.09 | 0.25 | 4332 | *MNDA* | 1233 | FALSE |  |
| cg17205788 | 1.85E-06 | 0.46 | 0.07 | 0.21 | 0.02 | 0.25 | 50945 | *TBX22* | 675 | FALSE |  |
| cg19111262 | 1.34E-05 | 0.46 | 0.08 | 0.21 | 0.05 | 0.25 | 57549 | *IGSF9* | 740 | FALSE |  |
| cg02075593 | 0.0010119 | 0.62 | 0.13 | 0.37 | 0.07 | 0.25 | 2940 | *GSTA3* | 785 | FALSE |  |
| cg02523400 | 5.90E-09 | 0.91 | 0.01 | 0.66 | 0.07 | 0.25 | 3053 | *SERPIND1* | 192 | FALSE |  |
| cg08981777 | 5.83E-07 | 0.36 | 0.06 | 0.11 | 0.02 | 0.25 | 64100 | *ELSPBP1* | 194 | FALSE |  |
| cg01305625 | 9.60E-06 | 0.53 | 0.09 | 0.28 | 0.04 | 0.25 | 8572 | *PDLIM4* | 425 | TRUE | 5:131620858-131621718 |
| cg27566805 | 2.92E-06 | 0.69 | 0.04 | 0.44 | 0.08 | 0.25 | 7399 | *USH2A* | 70 | FALSE |  |
| cg05606799 | 0.0001142 | 0.66 | 0.11 | 0.41 | 0.01 | 0.26 | 3814 | *KISS1* | 307 | FALSE |  |
| cg22143352 | 0.0002325 | 0.66 | 0.09 | 0.40 | 0.10 | 0.26 | 122953 | *JDP2* | 996 | FALSE |  |
| cg15967525 | 0.0003792 | 0.58 | 0.11 | 0.32 | 0.04 | 0.26 | 54502 | *FLJ20273* | 30 | FALSE |  |
| cg01185080 | 2.68E-06 | 0.49 | 0.08 | 0.24 | 0.03 | 0.26 | 374655 | *ZNF710* | 939 | TRUE | 15:88344274-88345300 |
| cg12120741 | 0.0004247 | 0.46 | 0.15 | 0.20 | 0.03 | 0.26 | 1910 | *EDNRB* | NA | TRUE | 13:77390197-77391989 |
| cg19408398 | 1.81E-07 | 0.55 | 0.05 | 0.29 | 0.04 | 0.26 | 81608 | *FIP1L1* | 535 | FALSE |  |
| cg05256043 | 1.81E-06 | 0.68 | 0.06 | 0.42 | 0.03 | 0.26 | 1638 | *DCT* | 28 | FALSE |  |
| cg21274025 | 8.20E-05 | 0.68 | 0.09 | 0.42 | 0.04 | 0.26 | 50487 | *PLA2G3* | 488 | FALSE |  |
| cg06317209 | 5.57E-06 | 0.54 | 0.08 | 0.28 | 0.04 | 0.26 | 10677 | *AVIL* | 1026 | FALSE |  |
| cg01058368 | 5.57E-06 | 0.48 | 0.09 | 0.22 | 0.03 | 0.26 | 1008 | *CDH10* | 576 | FALSE |  |
| cg26128441 | 4.53E-07 | 0.83 | 0.04 | 0.57 | 0.07 | 0.26 | 5024 | *P2RX3* | 610 | FALSE |  |
| cg22918700 | 0.000169 | 0.72 | 0.09 | 0.45 | 0.09 | 0.27 | 161247 | *LOC161247* | 701 | FALSE |  |
| cg00002426 | 5.98E-05 | 0.72 | 0.08 | 0.45 | 0.04 | 0.27 | 7871 | *SLMAP* | 369 | TRUE | 3:57716811-57718675 |
| cg25598083 | 0.0002327 | 0.60 | 0.11 | 0.33 | 0.09 | 0.27 | 10965 | *ACOT2* | 886 | FALSE |  |
| cg02512860 | 1.44E-05 | 0.71 | 0.07 | 0.44 | 0.05 | 0.27 | 24146 | *CLDN15* | 365 | FALSE |  |
| cg22805308 | 4.58E-08 | 0.47 | 0.05 | 0.21 | 0.03 | 0.27 | 57449 | *PLEKHG5* | 154 | FALSE |  |
| cg07044282 | 2.51E-07 | 0.52 | 0.06 | 0.25 | 0.03 | 0.27 | 9068 | *ANGPTL1* | 78 | FALSE |  |
| cg11251877 | 1.61E-05 | 0.83 | 0.04 | 0.56 | 0.12 | 0.27 | 26236 | *C6orf54* | 1224 | FALSE |  |
| cg26185508 | 0.0001553 | 0.51 | 0.11 | 0.24 | 0.08 | 0.27 | 200008 | *CDCP2* | 2 | TRUE | 1:54391846-54392065 |
| cg22337624 | 0.0021745 | 0.51 | 0.16 | 0.23 | 0.06 | 0.27 | 9785 | *DHX38* | 1194 | FALSE |  |
| cg02800334 | 7.51E-07 | 0.61 | 0.05 | 0.34 | 0.03 | 0.27 | 312 | *ANXA13* | 1208 | FALSE |  |
| cg27390220 | 0.0001115 | 0.48 | 0.14 | 0.21 | 0.03 | 0.27 | 25907 | *RIS1* | 769 | TRUE | 3:45241257-45243590 |
| cg10705251 | 1.61E-11 | 0.91 | 0.01 | 0.63 | 0.03 | 0.27 | 257101 | *ZNF683* | 339 | FALSE |  |
| cg01055695 | 3.07E-09 | 0.85 | 0.01 | 0.57 | 0.06 | 0.28 | 5047 | *PAEP* | 654 | FALSE |  |
| cg01530101 | 2.88E-06 | 0.37 | 0.07 | 0.09 | 0.04 | 0.28 | 55539 | *KCNQ1DN* | NA | TRUE | 11:2846828-2848015 |
| cg10222534 | 0.0001077 | 0.68 | 0.11 | 0.41 | 0.07 | 0.28 | 3770 | *KCNJ14* | 128 | FALSE |  |
| cg26757793 | 1.32E-05 | 0.76 | 0.03 | 0.48 | 0.09 | 0.28 | 9313 | *MMP20* | 96 | FALSE |  |
| cg01185754 | 6.13E-05 | 0.40 | 0.10 | 0.12 | 0.08 | 0.28 | 2152 | *F3* | 586 | TRUE | 1:94778962-94780728 |
| cg17349199 | 1.48E-07 | 0.73 | 0.05 | 0.45 | 0.04 | 0.28 | 143379 | *C10orf82* | 29 | FALSE |  |
| cg20625138 | 1.86E-06 | 0.70 | 0.05 | 0.42 | 0.07 | 0.28 | 10911 | *UTS2* | 95 | FALSE |  |
| cg26104469 | 1.05E-07 | 0.56 | 0.04 | 0.28 | 0.03 | 0.28 | 137868 | *SGCZ* | 750 | FALSE |  |
| cg11880010 | 6.83E-06 | 0.60 | 0.07 | 0.31 | 0.05 | 0.29 | 23273 | *KIAA0367* | 6 | FALSE |  |
| cg09595479 | 1.98E-05 | 0.61 | 0.11 | 0.32 | 0.04 | 0.29 | 5630 | *PRPH* | 303 | TRUE | 12:47975082-47977816 |
| cg17819635 | 0.0023528 | 0.42 | 0.19 | 0.13 | 0.09 | 0.29 | 200132 | *TCTEX1D1* | 469 | TRUE | 1:66990232-66990994 |
| cg08775230 | 1.34E-06 | 0.67 | 0.07 | 0.38 | 0.04 | 0.29 | 91894 | *C11orf52* | 144 | FALSE |  |
| cg03544320 | 0.0094805 | 0.40 | 0.09 | 0.11 | 0.10 | 0.29 | 1400 | *CRMP1* | 94 | TRUE | 4:5944246-5946191 |
| cg06470471 | 4.93E-06 | 0.84 | 0.05 | 0.55 | 0.08 | 0.29 | 57408 | *LRTM1* | 161 | FALSE |  |
| cg04893119 | 2.30E-05 | 0.47 | 0.12 | 0.18 | 0.04 | 0.29 | 51050 | *PI15* | 471 | FALSE |  |
| cg10414946 | 1.04E-07 | 0.58 | 0.05 | 0.29 | 0.04 | 0.29 | 2206 | *MS4A2* | 221 | FALSE |  |
| cg00862290 | 4.22E-06 | 0.83 | 0.04 | 0.54 | 0.09 | 0.30 | 27094 | *KCNMB3* | 135 | TRUE | 3:180467337-180467703 |
| cg23504246 | 5.98E-08 | 0.49 | 0.07 | 0.19 | 0.02 | 0.30 | 2252 | *FGF7* | 117 | FALSE |  |
| cg12339029 | 2.35E-08 | 0.88 | 0.02 | 0.58 | 0.06 | 0.30 | 4632 | *MYL1* | 5 | FALSE |  |
| cg03934354 | 8.71E-07 | 0.76 | 0.06 | 0.46 | 0.04 | 0.30 | 114899 | *C1QTNF3* | 177 | FALSE |  |
| cg13763232 | 0.0001228 | 0.43 | 0.17 | 0.13 | 0.03 | 0.30 | 6533 | *SLC6A6* | 678 | TRUE | 3:14418260-14418496 |
| cg27371741 | 3.75E-06 | 0.57 | 0.08 | 0.27 | 0.05 | 0.30 | 6997 | *TDGF1* | 55 | TRUE | 3:46594072-46594386 |
| cg09607232 | 8.88E-08 | 0.86 | 0.02 | 0.56 | 0.05 | 0.30 | 4108 | *MAGEA9* | 447 | FALSE |  |
| cg26514942 | 7.89E-06 | 0.66 | 0.08 | 0.36 | 0.07 | 0.31 | 130827 | *FLJ30294* | 29 | FALSE |  |
| cg13434842 | 8.10E-08 | 0.38 | 0.05 | 0.08 | 0.02 | 0.31 | 2626 | *GATA4* | NA | TRUE | 8:11605055-11605961 |
| cg06323290 | 1.39E-07 | 0.84 | 0.04 | 0.53 | 0.03 | 0.31 | 3098 | *HK1* | 123 | FALSE |  |
| cg06493930 | 8.24E-07 | 0.74 | 0.05 | 0.43 | 0.08 | 0.31 | 2620 | *GAS2* | 160 | FALSE |  |
| cg05232889 | 3.72E-09 | 0.42 | 0.04 | 0.11 | 0.03 | 0.31 | 93986 | *FOXP2* | 143 | FALSE |  |
| cg02498063 | 6.25E-07 | 0.46 | 0.10 | 0.14 | 0.04 | 0.32 | 160777 | *CCDC60* | 60 | TRUE | 12:118256646-118257076 |
| cg15966757 | 1.37E-05 | 0.68 | 0.11 | 0.36 | 0.06 | 0.32 | 6540 | *SLC6A13* | 257 | FALSE |  |
| cg07484827 | 1.05E-08 | 0.72 | 0.02 | 0.39 | 0.05 | 0.33 | 57053 | *CHRNA10* | 543 | FALSE |  |
| cg09522147 | 2.61E-06 | 0.59 | 0.04 | 0.27 | 0.07 | 0.33 | 3855 | *KRT7* | 388 | TRUE | 12:50912973-50913926 |
| cg17667972 | 2.02E-09 | 0.87 | 0.02 | 0.54 | 0.08 | 0.33 | 3851 | *KRT4* | 42 | FALSE |  |
| cg05859842 | 4.61E-08 | 0.47 | 0.08 | 0.14 | 0.03 | 0.33 | 143162 | *FRMPD2* | 443 | FALSE |  |
| cg26215428 | 2.77E-06 | 0.76 | 0.05 | 0.42 | 0.11 | 0.34 | 5311 | *PKD2* | 569 | FALSE |  |
| cg04765277 | 1.95E-07 | 0.58 | 0.08 | 0.24 | 0.04 | 0.34 | 399717 | *FLJ45983* | 768 | TRUE | 10:8130632-8134925 |
| cg15149938 | 0.0002241 | 0.60 | 0.17 | 0.26 | 0.06 | 0.34 | 348645 | *LOC348645* | 176 | FALSE |  |
| cg05397738 | 3.06E-06 | 0.78 | 0.06 | 0.44 | 0.08 | 0.34 | 10857 | *PGRMC1* | 692 | TRUE | X:118253585-118255522 |
| cg04256470 | 6.65E-08 | 0.86 | 0.05 | 0.51 | 0.03 | 0.35 | 1325 | *CORT* | 494 | TRUE | 1:10432949-10433150 |
| cg00779924 | 4.73E-06 | 0.41 | 0.18 | 0.06 | 0.01 | 0.35 | 399717 | *FLJ45983* | 806 | TRUE | 10:8130632-8134925 |
| cg02254461 | 0.0004678 | 0.54 | 0.20 | 0.19 | 0.05 | 0.35 | 64651 | *AXUD1* | 803 | TRUE | 3:39170826-39171090 |
| cg25189085 | 1.12E-06 | 0.73 | 0.07 | 0.37 | 0.06 | 0.35 | 246329 | *STAC3* | 311 | FALSE |  |
| cg02046017 | 9.67E-06 | 0.73 | 0.05 | 0.37 | 0.09 | 0.36 | 220070 | *LOC220070* | 1489 | FALSE |  |
| cg24829483 | 2.81E-08 | 0.72 | 0.03 | 0.35 | 0.06 | 0.37 | 114905 | *C1QTNF7* | 29 | FALSE |  |
| cg13980719 | 4.77E-06 | 0.67 | 0.11 | 0.29 | 0.08 | 0.38 | 7141 | *TNP1* | 778 | FALSE |  |
| cg07220939 | 9.54E-08 | 0.57 | 0.07 | 0.18 | 0.06 | 0.39 | 116085 | *SLC22A12* | 335 | TRUE | 11:64115128-64115334 |
| cg14345676 | 4.56E-06 | 0.66 | 0.10 | 0.27 | 0.05 | 0.39 | 3274 | *HRH2* | 614 | TRUE | 5:175041676-175041898 |
| cg20373326 | 1.61E-11 | 0.64 | 0.03 | 0.24 | 0.02 | 0.40 | 3294 | *HSD17B2* | 60 | FALSE |  |
| cg19485804 | 2.17E-06 | 0.76 | 0.02 | 0.36 | 0.12 | 0.40 | 25791 | *NGEF* | 776 | FALSE |  |
| cg16558203 | 2.96E-08 | 0.78 | 0.06 | 0.37 | 0.02 | 0.42 | 9389 | *SLC22A14* | 35 | FALSE |  |
| cg22286764 | 1.09E-08 | 0.83 | 0.04 | 0.41 | 0.07 | 0.42 | 339883 | *C3orf35* | 879 | FALSE |  |
| cg06590610 | 1.79E-10 | 0.83 | 0.05 | 0.40 | 0.02 | 0.43 | 10320 | *ZNFN1A1* | 86 | TRUE | 7:50411750-50412000 |
| cg23863670 | 3.89E-10 | 0.78 | 0.04 | 0.35 | 0.02 | 0.43 | 56477 | *CCL28* | 543 | FALSE |  |
| cg19910382 | 3.69E-10 | 0.83 | 0.03 | 0.27 | 0.02 | 0.56 | 2168 | *FABP1* | 17 | FALSE |  |
