# Supplementary material for: A Comparison of Genome-Wide DNA Methylation Patterns between Different Vascular Tissues from Patients with Coronary Heart Disease
Source: PLoS One. 2015 Apr 9;10(4):e0122601. doi: 10.1371/journal.pone.0122601 (PMC4391864; doi:10.1371/journal.pone.0122601)
Supplement: S2 Table — The results for each enriched GO category (biological process) are listed in the table. C—number of reference genes in the category; O—number of genes in the gene set and also in the category, E—expected number in the category, R—Ratio of enrichment, rawP—p value from hypergeometric test, and adjP—p value adjusted by the multiple test adjustment. (DOCX) [file pone.0122601.s002.docx]

**WebGestalt-generated significant ontologies for hypomethylated CpG-sites in CAP**

| **biological process inflammatory response**  **GO:0006954**  **C=442;O=17;E=4.38;R=3.88;rawP=1.58e-06;adjP=0.0016** | | | |
| --- | --- | --- | --- |
| Entrez ID | Gene symbol | | Description |
| 1269 | *CNR2* | | cannabinoid receptor 2 (macrophage) |
| 6347 | *CCL2* | | chemokine (C-C motif) ligand 2 |
| 3568 | *IL5RA* | | interleukin 5 receptor, alpha |
| 6279 | *S100A8* | | S100 calcium binding protein A8 |
| 1672 | *DEFB1* | | defensin, beta 1 |
| 2532 | *DARC* | | Duffy blood group, chemokine receptor |
| 5199 | *CFP* | | complement factor properdin |
| 6696 | *SPP1* | | secreted phosphoprotein 1 |
| 9447 | *AIM2* | | absent in melanoma 2 |
| 1232 | *CCR3* | | chemokine (C-C motif) receptor 3 |
| 9332 | *CD163* | | CD163 molecule |
| 7099 | *TLR4* | | toll-like receptor 4 |
| 27035 | *NOX1* | | NADPH oxidase 1 |
| 6850 | *SYK* | | spleen tyrosine kinase |
| 940 | *CD28* | | CD28 molecule |
| 3428 | *IFI16* | | interferon, gamma-inducible protein 16 |
| 140 | *ADORA3* | | adenosine A3 receptor |
| **biological process defense response to bacterium**  **GO:0042742**  **C=108;O=8;E=1.07;R=7.47;rawP=1.14e-05;adjP=0.0030** | | | |
| Entrez ID | Gene symbol | | Description |
| 6037 | *RNASE3* | | ribonuclease, RNase A family, 3 |
| 124912 | *SPACA3* | | sperm acrosome associated 3 |
| 6279 | *S100A8* | | S100 calcium binding protein A8 |
| 944 | *TNFSF8* | | tumor necrosis factor (ligand) superfamily, member 8 |
| 1672 | *DEFB1* | | defensin, beta 1 |
| 7099 | *TLR4* | | toll-like receptor 4 |
| 5199 | *CFP* | | complement factor properdin |
| 6850 | *SYK* | | spleen tyrosine kinase |
| **biological process embryonic skeletal system morphogenesis**  **GO:0048704**  **C=77;O=7;E=0.76;R=9.16;rawP=1.07e-05;adjP=0.0030** | | | |
| Entrez ID | | Gene symbol | Description |
| 3216 | | *HOXB6* | homeobox B6 |
| 3204 | | *HOXA7* | homeobox A7 |
| 3232 | | *HOXD3* | homeobox D3 |
| 3233 | | *HOXD4* | homeobox D4 |
| 60529 | | *ALX4* | ALX homeobox 4 |
| 3215 | | *HOXB5* | homeobox B5 |
| 3200 | | *HOXA3* | homeobox A3 |
| **biological process embryonic skeletal system development**  **GO:0048706 C=101;O=8;E=1.00;R=7.99;rawP=6.91e-06;adjP=0.0030** | | | |
| Entrez ID | | Gene symbol | Description |
| 3204 | | *HOXA7* | homeobox A7 |
| 3232 | | *HOXD3* | homeobox D3 |
| 3233 | | *HOXD4* | homeobox D4 |
| 3222 | | *HOXC5* | homeobox C5 |
| 60529 | | *ALX4* | ALX homeobox 4 |
| 3200 | | *HOXA3* | homeobox A3 |
| 3216 | | *HOXB6* | homeobox B6 |
| 3215 | | *HOXB5* | homeobox B5 |
| **biological process defense response**  **GO:0006952**  **C=975;O=24;E=9.67;R=2.48;rawP=2.49e-05;adjP=0.0052** | | | |
| Entrez ID | | Gene symbol | Description |
| 1269 | | *CNR2* | cannabinoid receptor 2 (macrophage) |
| 6037 | | *RNASE3* | ribonuclease, RNase A family, 3 |
| 6347 | | *CCL2* | chemokine (C-C motif) ligand 2 |
| 3568 | | *IL5RA* | interleukin 5 receptor, alpha |
| 3430 | | *IFI35* | interferon-induced protein 35 |
| 6279 | | *S100A8* | S100 calcium binding protein A8 |
| 1672 | | *DEFB1* | defensin, beta 1 |
| 944 | | *TNFSF8* | tumor necrosis factor (ligand) superfamily, member 8 |
| 5199 | | *CFP* | complement factor properdin |
| 2532 | | *DARC* | Duffy blood group, chemokine receptor |
| 6696 | | *SPP1* | secreted phosphoprotein 1 |
| 5696 | | *PSMB8* | proteasome (prosome, macropain) subunit, beta type, 8 (large multifunctional peptidase 7) |
| 9447 | | *AIM2* | absent in melanoma 2 |
| 1232 | | *CCR3* | chemokine (C-C motif) receptor 3 |
| 124912 | | *SPACA3* | sperm acrosome associated 3 |
| 11262 | | *SP140* | SP140 nuclear body protein |
| 9332 | | *CD163* | CD163 molecule |
| 3687 | | *ITGAX* | integrin, alpha X (complement component 3 receptor 4 subunit) |
| 7099 | | *TLR4* | toll-like receptor 4 |
| 940 | | *CD28* | CD28 molecule |
| 6850 | | *SYK* | spleen tyrosine kinase |
| 27035 | | *NOX1* | NADPH oxidase 1 |
| 3428 | | *IFI16* | interferon, gamma-inducible protein 16 |
| 140 | | *ADORA3* | adenosine A3 receptor |
| **biological process response to other organism**  **GO:0051707**  **C=509;O=16;E=5.05;R=3.17;rawP=4.07e-05;adjP=0.0070** | | | |
| Entrez ID | | Gene symbol | Description |
| 1269 | | *CNR2* | cannabinoid receptor 2 (macrophage) |
| 6037 | | *RNASE3* | ribonuclease, RNase A family, 3 |
| 9447 | | *AIM2* | absent in melanoma 2 |
| 6347 | | *CCL2* | chemokine (C-C motif) ligand 2 |
| 124912 | | *SPACA3* | sperm acrosome associated 3 |
| 356 | | *FASLG* | Fas ligand (TNF superfamily, member 6) |
| 6279 | | *S100A8* | S100 calcium binding protein A8 |
| 3430 | | *IFI35* | interferon-induced protein 35 |
| 1672 | | *DEFB1* | defensin, beta 1 |
| 944 | | *TNFSF8* | tumor necrosis factor (ligand) superfamily, member 8 |
| 3687 | | *ITGAX* | integrin, alpha X (complement component 3 receptor 4 subunit) |
| 7099 | | *TLR4* | toll-like receptor 4 |
| 940 | | *CD28* | CD28 molecule |
| 6850 | | *SYK* | spleen tyrosine kinase |
| 5199 | | *CFP* | complement factor properdin |
| 3428 | | *IFI16* | interferon, gamma-inducible protein 16 |
| **biological process response to biotic stimulus**  **GO:0009607**  **C=531;O=16;E=5.27;R=3.04;rawP=6.75e-05;adjP=0.0082** | | | |
| Entrez ID | | Gene symbol | Description |
| 1269 | | *CNR2* | cannabinoid receptor 2 (macrophage) |
| 6037 | | *RNASE3* | ribonuclease, RNase A family, 3 |
| 9447 | | *AIM2* | absent in melanoma 2 |
| 6347 | | *CCL2* | chemokine (C-C motif) ligand 2 |
| 124912 | | *SPACA3* | sperm acrosome associated 3 |
| 356 | | *FASLG* | Fas ligand (TNF superfamily, member 6) |
| 6279 | | *S100A8* | S100 calcium binding protein A8 |
| 3430 | | *IFI35* | interferon-induced protein 35 |
| 1672 | | *DEFB1* | defensin, beta 1 |
| 944 | | *TNFSF8* | tumor necrosis factor (ligand) superfamily, member 8 |
| 3687 | | *ITGAX* | integrin, alpha X (complement component 3 receptor 4 subunit) |
| 7099 | | *TLR4* | toll-like receptor 4 |
| 940 | | *CD28* | CD28 molecule |
| 6850 | | *SYK* | spleen tyrosine kinase |
| 5199 | | *CFP* | complement factor properdin |
| 3428 | | *IFI16* | interferon, gamma-inducible protein 16 |
| **biological process immune system process**  **GO:0002376**  **C=1617;O=32;E=16.04;R=2.00;rawP=7.11e-05;adjP=0.0082** | | | |
| Entrez ID | | Gene symbol | Description |
| 3204 | | *HOXA7* | homeobox A7 |
| 6347 | | *CCL2* | chemokine (C-C motif) ligand 2 |
| 3568 | | *IL5RA* | interleukin 5 receptor, alpha |
| 3430 | | *IFI35* | interferon-induced protein 35 |
| 6279 | | *S100A8* | S100 calcium binding protein A8 |
| 6943 | | *TCF21* | transcription factor 21 |
| 944 | | *TNFSF8* | tumor necrosis factor (ligand) superfamily, member 8 |
| 5133 | | *PDCD1* | programmed cell death 1 |
| 6696 | | *SPP1* | secreted phosphoprotein 1 |
| 5696 | | *PSMB8* | proteasome (prosome, macropain) subunit, beta type, 8 (large multifunctional peptidase 7) |
| 9447 | | *AIM2* | absent in melanoma 2 |
| 79679 | | *VTCN1* | V-set domain containing T cell activation inhibitor 1 |
| 915 | | *CD3D* | CD3d molecule, delta (CD3-TCR complex) |
| 4237 | | *MFAP2* | microfibrillar-associated protein 2 |
| 3687 | | *ITGAX* | integrin, alpha X (complement component 3 receptor 4 subunit) |
| 7099 | | *TLR4* | toll-like receptor 4 |
| 6850 | | *SYK* | spleen tyrosine kinase |
| 140 | | *ADORA3* | adenosine A3 receptor |
| 1269 | | *CNR2* | cannabinoid receptor 2 (macrophage) |
| 8530 | | *CST7* | cystatin F (leukocystatin) |
| 397 | | *ARHGDIB* | Rho GDP dissociation inhibitor (GDI) beta |
| 53347 | | *UBASH3A* | ubiquitin associated and SH3 domain containing A |
| 6318 | | *SERPINB4* | serpin peptidase inhibitor, clade B (ovalbumin), member 4 |
| 1672 | | *DEFB1* | defensin, beta 1 |
| 5199 | | *CFP* | complement factor properdin |
| 951 | | *CD37* | CD37 molecule |
| 124912 | | *SPACA3* | sperm acrosome associated 3 |
| 3200 | | *HOXA3* | homeobox A3 |
| 356 | | *FASLG* | Fas ligand (TNF superfamily, member 6) |
| 861 | | *RUNX1* | runt-related transcription factor 1 |
| 940 | | *CD28* | CD28 molecule |
| 3428 | | *IFI16* | interferon, gamma-inducible protein 16 |
| **biological process immune response**  **GO:0006955**  **C=958;O=23;E=9.50;R=2.42;rawP=5.61e-05;adjP=0.0082** | | | |
| Entrez ID | | Gene symbol | Description |
| 1269 | | *CNR2* | cannabinoid receptor 2 (macrophage) |
| 6347 | | *CCL2* | chemokine (C-C motif) ligand 2 |
| 8530 | | *CST7* | cystatin F (leukocystatin) |
| 3568 | | *IL5RA* | interleukin 5 receptor, alpha |
| 6279 | | *S100A8* | S100 calcium binding protein A8 |
| 3430 | | *IFI35* | interferon-induced protein 35 |
| 397 | | *ARHGDIB* | Rho GDP dissociation inhibitor (GDI) beta |
| 53347 | | *UBASH3A* | ubiquitin associated and SH3 domain containing A |
| 1672 | | *DEFB1* | defensin, beta 1 |
| 944 | | *TNFSF8* | tumor necrosis factor (ligand) superfamily, member 8 |
| 6318 | | *SERPINB4* | serpin peptidase inhibitor, clade B (ovalbumin), member 4 |
| 5199 | | *CFP* | complement factor properdin |
| 951 | | *CD37* | CD37 molecule |
| 5133 | | *PDCD1* | programmed cell death 1 |
| 5696 | | *PSMB8* | proteasome (prosome, macropain) subunit, beta type, 8 (large multifunctional peptidase 7) |
| 9447 | | *AIM2* | absent in melanoma 2 |
| 356 | | *FASLG* | Fas ligand (TNF superfamily, member 6) |
| 915 | | *CD3D* | CD3d molecule, delta (CD3-TCR complex) |
| 7099 | | *TLR4* | toll-like receptor 4 |
| 940 | | *CD28* | CD28 molecule |
| 6850 | | *SYK* | spleen tyrosine kinase |
| 3428 | | *IFI16* | interferon, gamma-inducible protein 16 |
| 140 | | *ADORA3* | adenosine A3 receptor |
| **biological process anterior/posterior pattern specification**  **GO:0009952**  **C=191;O=9;E=1.89;R=4.75;rawP=0.0001;adjP=0.0104** | | | |
| Entrez ID | | Gene symbol | Description |
| 3204 | | *HOXA7* | homeobox A7 |
| 3232 | | *HOXD3* | homeobox D3 |
| 7273 | | *TTN* | titin |
| 3233 | | *HOXD4* | homeobox D4 |
| 3222 | | *HOXC5* | homeobox C5 |
| 60529 | | *ALX4* | ALX homeobox 4 |
| 3200 | | *HOXA3* | homeobox A3 |
| 3216 | | *HOXB6* | homeobox B6 |
| 3215 | | *HOXB5* | homeobox B5 |
