# Supplementary material for: A Comparison of Genome-Wide DNA Methylation Patterns between Different Vascular Tissues from Patients with Coronary Heart Disease
Source: PLoS One. 2015 Apr 9;10(4):e0122601. doi: 10.1371/journal.pone.0122601 (PMC4391864; doi:10.1371/journal.pone.0122601)
Supplement: S3 Table — FDR-adjusted p<0.05 and delta beta ≥0.2 or delta beta ≤-0.2. (DOCX) [file pone.0122601.s003.docx]

**Differentially methylated CpG-sites between GSV and IMA groups. FDR-adjusted p<0.05 and delta beta ≥0.2 or delta beta ≤-0.2**

| **Probe ID** | **adj.P.Val** | **IMA_**  **beta** | **IMA_**  **beta**  **_SD** | **GSV_**  **beta** | **GSV_**  **beta**  **_SD** | **Average**  **delta beta**  **GSV-IMA** | **GENE_ID** | **SYMBOL** | **DISTANCE_**  **TO_TSS** | **CPG_ISLAND** | **CPG_ISLAND**  **_LOCATIONS** |
| --- | --- | --- | --- | --- | --- | --- | --- | --- | --- | --- | --- |
| cg02248486 | 3.42E-07 | 0.13 | 0.04 | 0.81 | 0.05 | 0.68 | 3202 | *HOXA5* | 91 | TRUE | 7:27147983-27152159 |
| cg26069745 | 2.93E-08 | 0.23 | 0.06 | 0.76 | 0.06 | 0.53 | 3199 | *HOXA2* | 194 | TRUE | 7:27108167-27108756 |
| cg01982597 | 4.12E-06 | 0.31 | 0.13 | 0.83 | 0.03 | 0.52 | 267004 | *PGBD3* | 1094 | FALSE |  |
| cg10044101 | 4.47E-07 | 0.22 | 0.10 | 0.72 | 0.07 | 0.51 | 8875 | *VNN2* | 309 | FALSE |  |
| cg04616566 | 9.09E-06 | 0.16 | 0.09 | 0.66 | 0.09 | 0.49 | 79875 | *THSD4* | 328 | FALSE |  |
| cg01963696 | 6.38E-06 | 0.17 | 0.08 | 0.64 | 0.07 | 0.47 | 1991 | *ELA2* | 641 | TRUE | 19:802526-803422 |
| cg18184219 | 2.58E-07 | 0.32 | 0.10 | 0.77 | 0.04 | 0.45 | 9859 | *CEP170* | 99 | FALSE |  |
| cg09871315 | 1.18E-07 | 0.36 | 0.08 | 0.81 | 0.05 | 0.45 | 3199 | *HOXA2* | 288 | TRUE | 7:27108930-27109394 |
| cg19530885 | 1.63E-08 | 0.27 | 0.06 | 0.72 | 0.03 | 0.44 | 117156 | *SCGB3A2* | 142 | FALSE |  |
| cg27210390 | 8.32E-06 | 0.23 | 0.10 | 0.67 | 0.08 | 0.44 | 10040 | *TOM1L1* | 379 | TRUE | 17:50332762-50333678 |
| cg04845579 | 1.62E-07 | 0.18 | 0.07 | 0.61 | 0.06 | 0.43 | 23213 | *SULF1* | 200 | FALSE |  |
| cg19224278 | 5.58E-06 | 0.29 | 0.08 | 0.72 | 0.06 | 0.42 | 220 | *ALDH1A3* | 1044 | TRUE | 15:99236455-99238833 |
| cg16098726 | 3.12E-05 | 0.39 | 0.09 | 0.81 | 0.08 | 0.42 | 2815 | *GP9* | 907 | TRUE | 3:130261348-130261588 |
| cg06317209 | 3.12E-08 | 0.28 | 0.04 | 0.69 | 0.05 | 0.41 | 10677 | *AVIL* | 1026 | FALSE |  |
| cg22264436 | 1.07E-07 | 0.44 | 0.10 | 0.85 | 0.01 | 0.40 | 50964 | *SOST* | 267 | TRUE | 17:39191915-39192117 |
| cg08124030 | 1.63E-08 | 0.16 | 0.03 | 0.56 | 0.07 | 0.40 | 4071 | *TM4SF1* | 285 | TRUE | 3:150577836-150578057 |
| cg13099330 | 1.06E-05 | 0.38 | 0.11 | 0.78 | 0.06 | 0.40 | 5947 | *RBP1* | 691 | TRUE | 3:140740275-140741996 |
| cg11500797 | 5.89E-06 | 0.18 | 0.11 | 0.57 | 0.11 | 0.39 | 1749 | *DLX5* | NA | TRUE | 7:96489769-96490524 |
| cg02075593 | 6.95E-06 | 0.37 | 0.07 | 0.76 | 0.06 | 0.39 | 2940 | *GSTA3* | 785 | FALSE |  |
| cg06444781 | 6.95E-08 | 0.49 | 0.08 | 0.88 | 0.02 | 0.39 | 6927 | *TCF1* | 39 | TRUE | 12:119900805-119901025 |
| cg10604646 | 1.03E-05 | 0.22 | 0.06 | 0.60 | 0.09 | 0.39 | 8490 | *RGS5* | 223 | FALSE |  |
| cg12128839 | 1.28E-06 | 0.04 | 0.01 | 0.43 | 0.08 | 0.39 | 3202 | *HOXA5* | 149 | TRUE | 7:27147983-27152159 |
| cg27214774 | 0.000644 | 0.28 | 0.10 | 0.66 | 0.03 | 0.38 | 140876 | *C20orf175* | 284 | FALSE |  |
| cg08319404 | 2.97E-06 | 0.32 | 0.11 | 0.70 | 0.05 | 0.38 | 7068 | *THRB* | 1122 | TRUE | 3:24510366-24512597 |
| cg23427666 | 1.11E-07 | 0.44 | 0.07 | 0.82 | 0.02 | 0.38 | 7038 | *TG* | 900 | TRUE | 8:133949132-133949367 |
| cg23350274 | 2.61E-05 | 0.40 | 0.09 | 0.78 | 0.03 | 0.37 | 145226 | *RDH12* | 527 | FALSE |  |
| cg10145926 | 1.07E-05 | 0.24 | 0.07 | 0.60 | 0.06 | 0.37 | 79686 | *C14orf139* | 635 | FALSE |  |
| cg19836199 | 2.06E-06 | 0.18 | 0.08 | 0.54 | 0.07 | 0.36 | 861 | *RUNX1* | 346 | FALSE |  |
| cg01185754 | 9.50E-06 | 0.12 | 0.08 | 0.48 | 0.09 | 0.36 | 2152 | *F3* | 586 | TRUE | 1:94778962-94780728 |
| cg22972055 | 8.65E-07 | 0.42 | 0.10 | 0.77 | 0.04 | 0.36 | 23353 | *UNC84A* | 155 | TRUE | 7:838536-838925 |
| cg27169020 | 2.18E-08 | 0.34 | 0.06 | 0.69 | 0.04 | 0.36 | 646 | *BNC1* | 761 | TRUE | 15:81742719-81745331 |
| cg14959707 | 3.44E-08 | 0.46 | 0.07 | 0.81 | 0.03 | 0.35 | 29066 | *ZC3H7A* | 17 | TRUE | 16:11783829-11784227 |
| cg16854606 | 2.35E-06 | 0.31 | 0.07 | 0.65 | 0.02 | 0.34 | 199699 | *DAND5* | 311 | FALSE |  |
| cg25189085 | 2.45E-06 | 0.37 | 0.06 | 0.71 | 0.06 | 0.34 | 246329 | *STAC3* | 311 | FALSE |  |
| cg26509022 | 8.80E-06 | 0.19 | 0.07 | 0.53 | 0.04 | 0.34 | 220 | *ALDH1A3* | NA | TRUE | 15:99236455-99238833 |
| cg27652350 | 2.23E-05 | 0.22 | 0.09 | 0.56 | 0.04 | 0.34 | 220 | *ALDH1A3* | NA | TRUE | 15:99236455-99238833 |
| cg02554564 | 4.02E-06 | 0.41 | 0.03 | 0.74 | 0.04 | 0.34 | 4908 | *NTF3* | 135 | TRUE | 12:5473265-5473465 |
| cg11170796 | 6.95E-08 | 0.41 | 0.04 | 0.74 | 0.04 | 0.33 | 6929 | *TCF3* | 53 | FALSE |  |
| cg21944455 | 1.11E-05 | 0.41 | 0.09 | 0.74 | 0.02 | 0.33 | 2670 | *GFAP* | 1401 | FALSE |  |
| cg11375102 | 1.72E-08 | 0.09 | 0.03 | 0.42 | 0.04 | 0.33 | 79652 | *C16orf30* | 152 | TRUE | 16:1523788-1524691 |
| cg09851465 | 6.65E-05 | 0.32 | 0.03 | 0.65 | 0.06 | 0.33 | 127795 | *C1orf87* | 245 | TRUE | 1:60311832-60312293 |
| cg06776256 | 1.03E-06 | 0.18 | 0.07 | 0.51 | 0.05 | 0.33 | 9052 | *GPRC5A* | 228 | TRUE | 12:12934727-12935045 |
| cg05859842 | 6.99E-08 | 0.14 | 0.03 | 0.46 | 0.06 | 0.32 | 143162 | *FRMPD2* | 443 | FALSE |  |
| cg18680834 | 5.27E-05 | 0.35 | 0.06 | 0.67 | 0.10 | 0.32 | 9745 | *ZNF536* | 21 | FALSE |  |
| cg16855929 | 1.25E-05 | 0.34 | 0.07 | 0.66 | 0.06 | 0.32 | 54583 | *EGLN1* | 624 | FALSE |  |
| cg26661623 | 0.000488 | 0.31 | 0.15 | 0.63 | 0.04 | 0.32 | 433 | *ASGR2* | 1183 | FALSE |  |
| cg14256699 | 4.24E-07 | 0.62 | 0.13 | 0.94 | 0.00 | 0.32 | 50964 | *SOST* | 130 | TRUE | 17:39191384-39191613 |
| cg08128768 | 1.31E-05 | 0.11 | 0.08 | 0.43 | 0.04 | 0.32 | 153090 | *DAB2IP* | 533 | TRUE | 9:123500662-123502056 |
| cg22407458 | 9.18E-05 | 0.37 | 0.09 | 0.68 | 0.05 | 0.32 | 6954 | *TCP11* | 5 | TRUE | 6:35216605-35217470 |
| cg19485804 | 4.84E-05 | 0.36 | 0.12 | 0.68 | 0.08 | 0.31 | 25791 | *NGEF* | 776 | FALSE |  |
| cg12041387 | 1.63E-05 | 0.26 | 0.09 | 0.57 | 0.10 | 0.31 | 1749 | *DLX5* | NA | TRUE | 7:96487969-96489640 |
| cg10226546 | 2.69E-05 | 0.27 | 0.06 | 0.58 | 0.05 | 0.31 | 4773 | *NFATC2* | 262 | TRUE | 20:49592243-49593041 |
| cg13462129 | 5.32E-05 | 0.28 | 0.09 | 0.59 | 0.11 | 0.31 | 1749 | *DLX5* | NA | TRUE | 7:96487969-96489640 |
| cg17998964 | 3.00E-06 | 0.29 | 0.06 | 0.59 | 0.03 | 0.31 | 2011 | *MARK2* | 172 | FALSE |  |
| cg23732182 | 7.20E-07 | 0.44 | 0.08 | 0.75 | 0.03 | 0.30 | 114038 | *C21orf84* | 13 | FALSE |  |
| cg24794433 | 8.78E-05 | 0.11 | 0.08 | 0.42 | 0.03 | 0.30 | 153090 | *DAB2IP* | 523 | TRUE | 9:123500662-123502056 |
| cg07220939 | 1.60E-06 | 0.18 | 0.06 | 0.49 | 0.06 | 0.30 | 116085 | *SLC22A12* | 335 | TRUE | 11:64115128-64115334 |
| cg13673094 | 7.30E-05 | 0.42 | 0.08 | 0.72 | 0.04 | 0.30 | 5788 | *PTPRC* | 136 | FALSE |  |
| cg21902327 | 1.69E-08 | 0.52 | 0.05 | 0.82 | 0.02 | 0.30 | 2251 | *FGF6* | 6 | TRUE | 12:4425015-4425387 |
| cg17118262 | 0.002305 | 0.37 | 0.08 | 0.67 | 0.07 | 0.30 | 6346 | *CCL1* | 317 | FALSE |  |
| cg26128441 | 6.95E-08 | 0.57 | 0.07 | 0.87 | 0.02 | 0.30 | 5024 | *P2RX3* | 610 | FALSE |  |
| cg18096388 | 3.88E-07 | 0.32 | 0.03 | 0.61 | 0.03 | 0.29 | 5133 | *PDCD1* | 85 | FALSE |  |
| cg06597095 | 4.63E-06 | 0.38 | 0.07 | 0.67 | 0.03 | 0.29 | 64417 | *FLJ21657* | 271 | FALSE |  |
| cg20080624 | 5.13E-05 | 0.31 | 0.06 | 0.60 | 0.11 | 0.29 | 1749 | *DLX5* | NA | TRUE | 7:96487969-96489640 |
| cg18675600 | 1.25E-05 | 0.42 | 0.06 | 0.71 | 0.05 | 0.29 | 11156 | *PTP4A3* | 234 | TRUE | 8:142501394-142501655 |
| cg09607232 | 1.91E-07 | 0.56 | 0.05 | 0.85 | 0.05 | 0.29 | 4108 | *MAGEA9* | 447 | FALSE |  |
| cg24861272 | 8.64E-05 | 0.31 | 0.05 | 0.60 | 0.05 | 0.29 | 6935 | *TCF8* | 989 | TRUE | 10:31647064-31650188 |
| cg19229991 | 1.52E-07 | 0.54 | 0.02 | 0.83 | 0.02 | 0.29 | 79785 | *FLJ22655* | 565 | FALSE |  |
| cg20610181 | 8.37E-06 | 0.37 | 0.07 | 0.66 | 0.04 | 0.29 | 768 | *CA9* | 85 | FALSE |  |
| cg02099418 | 0.000291 | 0.50 | 0.06 | 0.79 | 0.05 | 0.29 | 940 | *CD28* | 52 | FALSE |  |
| cg08403419 | 2.39E-05 | 0.18 | 0.04 | 0.47 | 0.04 | 0.29 | 339403 | *RLN3R2* | 263 | FALSE |  |
| cg12339029 | 6.29E-08 | 0.58 | 0.06 | 0.87 | 0.03 | 0.29 | 4632 | *MYL1* | 5 | FALSE |  |
| cg16051685 | 0.000613 | 0.29 | 0.04 | 0.58 | 0.07 | 0.28 | 84676 | *TRIM63* | 38 | FALSE |  |
| cg09201719 | 6.95E-08 | 0.64 | 0.06 | 0.93 | 0.02 | 0.28 | 1586 | *CYP17A1* | 288 | FALSE |  |
| cg15966757 | 8.85E-05 | 0.36 | 0.06 | 0.64 | 0.05 | 0.28 | 6540 | *SLC6A13* | 257 | FALSE |  |
| cg24901474 | 1.71E-05 | 0.16 | 0.07 | 0.44 | 0.04 | 0.28 | 8490 | *RGS5* | 168 | FALSE |  |
| cg10414946 | 2.08E-07 | 0.29 | 0.04 | 0.57 | 0.03 | 0.28 | 2206 | *MS4A2* | 221 | FALSE |  |
| cg20725021 | 0.000299 | 0.28 | 0.10 | 0.56 | 0.03 | 0.28 | 5648 | *MASP1* | 177 | FALSE |  |
| cg12910797 | 0.002308 | 0.37 | 0.07 | 0.65 | 0.14 | 0.28 | 3213 | *HOXB3* | 88 | TRUE | 17:44006560-44006958 |
| cg17095797 | 0.002915 | 0.20 | 0.08 | 0.48 | 0.08 | 0.28 | 2300 | *FOXL1* | 317 | FALSE |  |
| cg27182761 | 0.000174 | 0.33 | 0.11 | 0.61 | 0.07 | 0.28 | 22822 | *PHLDA1* | 1076 | TRUE | 12:74710025-74712942 |
| cg15749858 | 0.001462 | 0.20 | 0.03 | 0.47 | 0.09 | 0.28 | 346389 | *7A5* | 17 | FALSE |  |
| cg04151683 | 8.64E-05 | 0.26 | 0.05 | 0.53 | 0.09 | 0.28 | 158038 | *LRRN6C* | 844 | FALSE |  |
| cg05200628 | 0.00071 | 0.37 | 0.04 | 0.64 | 0.04 | 0.27 | 962 | *CD48* | 175 | FALSE |  |
| cg15760840 | 7.13E-05 | 0.17 | 0.09 | 0.44 | 0.09 | 0.27 | 3207 | *HOXA11* | 392 | TRUE | 7:27189699-27192279 |
| cg03886898 | 1.53E-06 | 0.53 | 0.07 | 0.80 | 0.05 | 0.27 | 1733 | *DIO1* | 729 | FALSE |  |
| cg14283939 | 5.28E-07 | 0.67 | 0.12 | 0.93 | 0.01 | 0.27 | 79875 | *THSD4* | 20 | TRUE | 15:69807869-69808151 |
| cg06204948 | 0.000295 | 0.47 | 0.05 | 0.73 | 0.06 | 0.26 | 2011 | *MARK2* | 15 | FALSE |  |
| cg06470471 | 2.70E-05 | 0.55 | 0.08 | 0.81 | 0.04 | 0.26 | 57408 | *LRTM1* | 161 | FALSE |  |
| cg04551925 | 6.52E-06 | 0.53 | 0.09 | 0.80 | 0.02 | 0.26 | 358 | *AQP1* | 272 | TRUE | 7:30918039-30918453 |
| cg01281904 | 0.00048 | 0.27 | 0.05 | 0.53 | 0.05 | 0.26 | 4986 | *OPRK1* | 563 | TRUE | 8:54327302-54327509 |
| cg17667972 | 2.29E-07 | 0.54 | 0.08 | 0.80 | 0.02 | 0.26 | 3851 | *KRT4* | 42 | FALSE |  |
| cg25711779 | 4.96E-06 | 0.12 | 0.05 | 0.38 | 0.08 | 0.26 | 2202 | *EFEMP1* | 353 | TRUE | 2:56003158-56003360 |
| cg22392708 | 1.48E-06 | 0.11 | 0.04 | 0.37 | 0.03 | 0.26 | 25984 | *KRT23* | 66 | FALSE |  |
| cg09584711 | 0.000191 | 0.31 | 0.10 | 0.57 | 0.04 | 0.26 | 3250 | *HPR* | 181 | FALSE |  |
| cg09522147 | 3.95E-05 | 0.27 | 0.07 | 0.52 | 0.07 | 0.26 | 3855 | *KRT7* | 388 | TRUE | 12:50912973-50913926 |
| cg20289911 | 2.67E-06 | 0.13 | 0.05 | 0.38 | 0.04 | 0.26 | 83541 | *C20orf55* | 349 | FALSE |  |
| cg02148642 | 6.60E-05 | 0.39 | 0.10 | 0.64 | 0.03 | 0.25 | 84220 | *RGPD5* | 749 | FALSE |  |
| cg16713727 | 2.08E-07 | 0.55 | 0.05 | 0.81 | 0.02 | 0.25 | 284723 | *SLC25A34* | 89 | FALSE |  |
| cg20804101 | 2.13E-05 | 0.17 | 0.03 | 0.43 | 0.06 | 0.25 | 6540 | *SLC6A13* | 86 | FALSE |  |
| cg27208307 | 0.000519 | 0.23 | 0.04 | 0.48 | 0.09 | 0.25 | 5552 | *PRG1* | 429 | FALSE |  |
| cg00503840 | 2.34E-05 | 0.46 | 0.06 | 0.71 | 0.07 | 0.25 | 1749 | *DLX5* | NA | TRUE | 7:96487969-96489640 |
| cg02037013 | 0.000448 | 0.31 | 0.04 | 0.56 | 0.04 | 0.25 | 4160 | *MC4R* | 52 | FALSE |  |
| cg03317245 | 0.000751 | 0.24 | 0.05 | 0.50 | 0.03 | 0.25 | 5026 | *P2RX5* | 931 | FALSE |  |
| cg09906458 | 0.000215 | 0.41 | 0.04 | 0.66 | 0.06 | 0.25 | 23627 | *PRND* | 25 | FALSE |  |
| cg00298951 | 0.00022 | 0.26 | 0.08 | 0.51 | 0.01 | 0.25 | 1240 | *CMKLR1* | 709 | TRUE | 12:107257760-107257967 |
| cg02084087 | 2.05E-05 | 0.25 | 0.05 | 0.49 | 0.04 | 0.25 | 8718 | *TNFRSF25* | 206 | TRUE | 1:6448532-6449318 |
| cg21350115 | 1.89E-06 | 0.10 | 0.04 | 0.35 | 0.06 | 0.25 | 10203 | *CALCRL* | 145 | FALSE |  |
| cg20951444 | 0.000646 | 0.34 | 0.06 | 0.58 | 0.09 | 0.24 | 252995 | *FNDC5* | 765 | FALSE |  |
| cg16301617 | 7.83E-05 | 0.31 | 0.09 | 0.55 | 0.03 | 0.24 | 11322 | *TMC6* | 418 | FALSE |  |
| cg16213655 | 0.000964 | 0.15 | 0.07 | 0.39 | 0.04 | 0.24 | 5493 | *PPL* | 396 | TRUE | 16:4926063-4927963 |
| cg20625138 | 1.52E-05 | 0.42 | 0.07 | 0.66 | 0.04 | 0.24 | 10911 | *UTS2* | 95 | FALSE |  |
| cg04502814 | 5.94E-05 | 0.16 | 0.05 | 0.40 | 0.07 | 0.24 | 6414 | *SEPP1* | 72 | FALSE |  |
| cg15350036 | 0.000282 | 0.47 | 0.09 | 0.71 | 0.04 | 0.24 | 54677 | *CROT* | 1334 | FALSE |  |
| cg01446692 | 0.00049 | 0.46 | 0.09 | 0.71 | 0.07 | 0.24 | 9350 | *CER1* | 781 | FALSE |  |
| cg12927617 | 5.27E-05 | 0.37 | 0.07 | 0.61 | 0.06 | 0.24 | 5004 | *ORM1* | 734 | FALSE |  |
| cg18879041 | 0.002089 | 0.16 | 0.07 | 0.40 | 0.07 | 0.24 | 96626 | *LIMS3* | 528 | TRUE | 2:110013888-110014175 |
| cg24115040 | 7.50E-06 | 0.10 | 0.05 | 0.34 | 0.09 | 0.24 | 1749 | *DLX5* | NA | TRUE | 7:96489769-96490524 |
| cg21453309 | 0.001042 | 0.31 | 0.07 | 0.55 | 0.04 | 0.24 | 144347 | *FAM101A* | 345 | FALSE |  |
| cg05740244 | 0.000429 | 0.33 | 0.06 | 0.57 | 0.03 | 0.24 | 3948 | *LDHC* | 162 | TRUE | 11:18389965-18390649 |
| cg02805028 | 0.000103 | 0.25 | 0.08 | 0.49 | 0.04 | 0.24 | 146395 | *MGC18079* | 239 | FALSE |  |
| cg23191950 | 8.58E-05 | 0.56 | 0.06 | 0.80 | 0.03 | 0.24 | 220 | *ALDH1A3* | 987 | TRUE | 15:99236455-99238833 |
| cg10851775 | 8.21E-07 | 0.55 | 0.06 | 0.78 | 0.03 | 0.24 | 115111 | *SLC26A7* | 1044 | FALSE |  |
| cg19731122 | 1.15E-08 | 0.34 | 0.03 | 0.57 | 0.03 | 0.24 | 4641 | *MYO1C* | 400 | FALSE |  |
| cg00585790 | 0.004042 | 0.36 | 0.13 | 0.59 | 0.09 | 0.23 | 3987 | *LIMS1* | 78 | FALSE |  |
| cg20582779 | 0.000133 | 0.30 | 0.03 | 0.54 | 0.05 | 0.23 | 79802 | *KIAA1822L* | 69 | FALSE |  |
| cg22143352 | 0.00074 | 0.40 | 0.10 | 0.63 | 0.02 | 0.23 | 122953 | *JDP2* | 996 | FALSE |  |
| cg25119415 | 1.57E-05 | 0.59 | 0.09 | 0.83 | 0.03 | 0.23 | 4332 | *MNDA* | 1233 | FALSE |  |
| cg05253327 | 1.64E-05 | 0.54 | 0.07 | 0.77 | 0.02 | 0.23 | 10678 | *B3GNT1* | 161 | FALSE |  |
| cg06436504 | 0.001522 | 0.44 | 0.06 | 0.67 | 0.06 | 0.23 | 11259 | *DOC1* | 378 | FALSE |  |
| cg26521448 | 1.69E-08 | 0.72 | 0.06 | 0.95 | 0.01 | 0.23 | 29066 | *ZC3H7A* | 18 | TRUE | 16:11783829-11784227 |
| cg10287137 | 5.89E-06 | 0.54 | 0.06 | 0.77 | 0.05 | 0.23 | 5029 | *P2RY2* | 290 | TRUE | 11:72606620-72607619 |
| cg06531741 | 0.000629 | 0.29 | 0.07 | 0.52 | 0.04 | 0.23 | 9177 | *HTR3B* | 139 | FALSE |  |
| cg13760253 | 1.87E-07 | 0.54 | 0.03 | 0.77 | 0.02 | 0.23 | 85479 | *DNAJC5B* | 1095 | FALSE |  |
| cg16176600 | 0.004652 | 0.20 | 0.10 | 0.43 | 0.11 | 0.23 | 2444 | *FRK* | 312 | FALSE |  |
| cg02523400 | 5.55E-08 | 0.66 | 0.07 | 0.89 | 0.02 | 0.23 | 3053 | *SERPIND1* | 192 | FALSE |  |
| cg20588069 | 8.49E-07 | 0.30 | 0.03 | 0.53 | 0.04 | 0.23 | 4487 | *MSX1* | 967 | TRUE | 4:4908144-4911629 |
| cg13277939 | 5.29E-05 | 0.16 | 0.06 | 0.39 | 0.08 | 0.23 | 4253 | *CTAGE5* | 715 | FALSE |  |
| cg21409833 | 0.000102 | 0.62 | 0.11 | 0.85 | 0.04 | 0.23 | 27329 | *ANGPTL3* | 463 | FALSE |  |
| cg13109289 | 1.63E-08 | 0.62 | 0.04 | 0.84 | 0.02 | 0.23 | 56913 | *C1GALT1* | 893 | FALSE |  |
| cg11822964 | 0.002754 | 0.20 | 0.03 | 0.43 | 0.05 | 0.23 | 5739 | *PTGIR* | 983 | TRUE | 19:51821132-51821336 |
| cg10705251 | 4.87E-09 | 0.63 | 0.03 | 0.86 | 0.02 | 0.23 | 257101 | *ZNF683* | 339 | FALSE |  |
| cg25322008 | 0.000847 | 0.27 | 0.05 | 0.50 | 0.11 | 0.23 | 81285 | *OR51E2* | 66 | FALSE |  |
| cg24694549 | 0.000629 | 0.34 | 0.06 | 0.56 | 0.05 | 0.23 | 23426 | *GRIP1* | 150 | FALSE |  |
| cg17819635 | 0.012052 | 0.13 | 0.09 | 0.35 | 0.13 | 0.22 | 200132 | *TCTEX1D1* | 469 | TRUE | 1:66990232-66990994 |
| cg02101486 | 1.65E-05 | 0.12 | 0.04 | 0.35 | 0.07 | 0.22 | 1749 | *DLX5* | NA | TRUE | 7:96489769-96490524 |
| cg00648883 | 0.012329 | 0.41 | 0.08 | 0.64 | 0.10 | 0.22 | 196549 | *LOC196549* | 465 | FALSE |  |
| cg14360917 | 0.005011 | 0.25 | 0.06 | 0.47 | 0.02 | 0.22 | 6668 | *SP2* | 556 | FALSE |  |
| cg12188560 | 6.99E-08 | 0.04 | 0.02 | 0.26 | 0.07 | 0.22 | 1271 | *CNTFR* | 1470 | TRUE | 9:34581146-34582071 |
| cg08077345 | 0.000174 | 0.28 | 0.04 | 0.51 | 0.04 | 0.22 | 57664 | *PLEKHA4* | 415 | FALSE |  |
| cg24840099 | 0.001206 | 0.59 | 0.07 | 0.82 | 0.05 | 0.22 | 4487 | *MSX1* | NA | TRUE | 4:4915141-4915899 |
| cg13842648 | 2.96E-07 | 0.50 | 0.03 | 0.72 | 0.02 | 0.22 | 3578 | *IL9* | 10 | FALSE |  |
| cg07072643 | 0.001988 | 0.55 | 0.06 | 0.78 | 0.06 | 0.22 | 84658 | *EMR3* | 217 | FALSE |  |
| cg01337047 | 4.11E-05 | 0.17 | 0.05 | 0.39 | 0.05 | 0.22 | 1828 | *DSG1* | 939 | FALSE |  |
| cg07404485 | 3.09E-06 | 0.45 | 0.03 | 0.68 | 0.03 | 0.22 | 5444 | *PON1* | 191 | FALSE |  |
| cg19111262 | 4.54E-05 | 0.21 | 0.05 | 0.43 | 0.04 | 0.22 | 57549 | *IGSF9* | 740 | FALSE |  |
| cg25410053 | 0.00149 | 0.37 | 0.03 | 0.59 | 0.14 | 0.22 | 7547 | *ZIC3* | 929 | TRUE | X:136475072-136477977 |
| cg26170660 | 0.000202 | 0.16 | 0.05 | 0.38 | 0.10 | 0.22 | 2880 | *GPX5* | 188 | FALSE |  |
| cg19890739 | 0.003569 | 0.39 | 0.06 | 0.61 | 0.06 | 0.22 | 51659 | *Pfs2* | 959 | TRUE | 16:84280985-84281211 |
| cg12374721 | 0.002273 | 0.20 | 0.03 | 0.41 | 0.15 | 0.22 | 84366 | *PRAC* | 242 | TRUE | 17:44154292-44154687 |
| cg27016494 | 8.71E-05 | 0.39 | 0.03 | 0.61 | 0.07 | 0.22 | 1749 | *DLX5* | NA | TRUE | 7:96487969-96489640 |
| cg17356733 | 8.85E-05 | 0.57 | 0.04 | 0.79 | 0.05 | 0.22 | 3460 | *IFNGR2* | 575 | TRUE | 21:33696159-33696518 |
| cg21480743 | 0.000178 | 0.10 | 0.05 | 0.32 | 0.04 | 0.22 | 5728 | *PTEN* | NA | TRUE | 10:89611198-89614163 |
| cg26873164 | 1.08E-06 | 0.17 | 0.04 | 0.39 | 0.02 | 0.22 | 6615 | *SNAI1* | 552 | TRUE | 20:48032014-48033196 |
| cg14607642 | 6.76E-05 | 0.41 | 0.05 | 0.63 | 0.04 | 0.22 | 3930 | *LBR* | 511 | FALSE |  |
| cg21197871 | 1.09E-05 | 0.22 | 0.06 | 0.44 | 0.05 | 0.22 | 137682 | *C8orf38* | 419 | TRUE | 8:96105896-96106919 |
| cg10919204 | 0.004049 | 0.20 | 0.09 | 0.42 | 0.11 | 0.21 | 1004 | *CDH6* | 456 | FALSE |  |
| cg09214551 | 6.92E-07 | 0.34 | 0.04 | 0.55 | 0.04 | 0.21 | 79877 | *DCAKD* | 1331 | FALSE |  |
| cg02729303 | 0.00139 | 0.14 | 0.05 | 0.36 | 0.08 | 0.21 | 54463 | *FLJ20152* | 17 | FALSE |  |
| cg04549333 | 0.002713 | 0.26 | 0.08 | 0.48 | 0.11 | 0.21 | 60529 | *ALX4* | NA | TRUE | 11:44282161-44283221 |
| cg09022808 | 5.27E-05 | 0.25 | 0.05 | 0.46 | 0.07 | 0.21 | 9514 | *GAL3ST1* | 19 | FALSE |  |
| cg27600794 | 1.61E-05 | 0.67 | 0.08 | 0.88 | 0.02 | 0.21 | 5407 | *PNLIPRP1* | 1060 | FALSE |  |
| cg02706881 | 6.24E-05 | 0.60 | 0.07 | 0.82 | 0.04 | 0.21 | 378832 | *C21orf123* | 790 | FALSE |  |
| cg18343292 | 1.16E-05 | 0.20 | 0.01 | 0.41 | 0.05 | 0.21 | 58475 | *MS4A7* | 120 | FALSE |  |
| cg16752583 | 0.011887 | 0.34 | 0.11 | 0.55 | 0.04 | 0.21 | 55503 | *TRPV6* | 254 | FALSE |  |
| cg19282250 | 0.000127 | 0.56 | 0.03 | 0.77 | 0.02 | 0.21 | 23273 | *KIAA0367* | 116 | FALSE |  |
| cg04806409 | 1.15E-06 | 0.60 | 0.02 | 0.81 | 0.02 | 0.21 | 7033 | *TFF3* | 201 | FALSE |  |
| cg20066677 | 3.06E-06 | 0.56 | 0.04 | 0.77 | 0.02 | 0.21 | 55743 | *CHFR* | NA | TRUE | 12:131934293-131934917 |
| cg18085435 | 0.010181 | 0.40 | 0.05 | 0.60 | 0.11 | 0.21 | 5205 | *ATP8B1* | 1128 | TRUE | 18:53550846-53551198 |
| cg25462303 | 0.002948 | 0.23 | 0.03 | 0.44 | 0.08 | 0.21 | 257144 | *GCET2* | 39 | FALSE |  |
| cg13060154 | 0.008826 | 0.19 | 0.05 | 0.40 | 0.03 | 0.21 | 153090 | *DAB2IP* | NA | TRUE | 9:123500662-123502056 |
| cg07810156 | 2.05E-05 | 0.53 | 0.06 | 0.74 | 0.02 | 0.21 | 10158 | *PDZK1IP1* | 89 | FALSE |  |
| cg05569220 | 1.39E-05 | 0.35 | 0.05 | 0.56 | 0.02 | 0.21 | 388428 | *FLJ44861* | 434 | FALSE |  |
| cg18873386 | 3.49E-06 | 0.37 | 0.03 | 0.58 | 0.06 | 0.21 | 1749 | *DLX5* | NA | TRUE | 7:96489769-96490524 |
| cg25447894 | 0.006297 | 0.15 | 0.07 | 0.36 | 0.08 | 0.21 | 27254 | *CSDC2* | 376 | FALSE |  |
| cg01566404 | 0.000542 | 0.39 | 0.08 | 0.60 | 0.04 | 0.21 | 55224 | *ETNK2* | 903 | FALSE |  |
| cg20988616 | 0.000427 | 0.43 | 0.09 | 0.64 | 0.04 | 0.20 | 5169 | *ENPP3* | 331 | FALSE |  |
| cg24335149 | 0.00635 | 0.54 | 0.09 | 0.75 | 0.06 | 0.20 | 5322 | *PLA2G5* | 236 | FALSE |  |
| cg15149938 | 0.021615 | 0.26 | 0.06 | 0.47 | 0.03 | 0.20 | 348645 | *LOC348645* | 176 | FALSE |  |
| cg01055695 | 6.82E-07 | 0.57 | 0.06 | 0.78 | 0.02 | 0.20 | 5047 | *PAEP* | 654 | FALSE |  |
| cg27063986 | 0.020474 | 0.38 | 0.06 | 0.58 | 0.04 | 0.20 | 64579 | *NDST4* | 200 | FALSE |  |
| cg04907257 | 7.30E-05 | 0.27 | 0.04 | 0.48 | 0.02 | 0.20 | 108 | *ADCY2* | 1027 | TRUE | 5:7447893-7448574 |
| cg05140736 | 6.45E-05 | 0.56 | 0.06 | 0.77 | 0.04 | 0.20 | 6448 | *SGSH* | 1394 | FALSE |  |
| cg02105856 | 1.46E-05 | 0.49 | 0.05 | 0.70 | 0.03 | 0.20 | 5284 | *PIGR* | 23 | FALSE |  |
| cg22518733 | 0.000166 | 0.12 | 0.03 | 0.33 | 0.03 | 0.20 | 6348 | *CCL3* | 88 | FALSE |  |
| cg15824056 | 4.62E-06 | 0.25 | 0.04 | 0.46 | 0.01 | 0.20 | 8871 | *SYNJ2* | 658 | TRUE | 6:158321912-158323549 |
| cg02218324 | 1.62E-05 | 0.39 | 0.04 | 0.59 | 0.05 | 0.20 | 81492 | *RSHL1* | 138 | TRUE | 19:51010186-51011161 |
| cg19297688 | 0.000301 | 0.66 | 0.10 | 0.86 | 0.02 | 0.20 | 3641 | *INSL4* | 59 | FALSE |  |
| cg25787984 | 0.000427 | 0.35 | 0.07 | 0.55 | 0.03 | 0.20 | 27122 | *DKK3* | 591 | FALSE |  |
| cg17589341 | 0.00279 | 0.28 | 0.10 | 0.48 | 0.06 | 0.20 | 6563 | *SLC14A1* | 78 | FALSE |  |
| cg25021182 | 1.16E-05 | 0.77 | 0.05 | 0.57 | 0.04 | -0.20 | 51032 | *ELA2B* | 2 | FALSE |  |
| cg20073553 | 0.000441 | 0.33 | 0.05 | 0.13 | 0.02 | -0.20 | 579 | *BAPX1* | 747 | TRUE | 4:13154295-13155884 |
| cg01656216 | 0.000183 | 0.41 | 0.03 | 0.21 | 0.05 | -0.20 | 220929 | *ZNF438* | 355 | FALSE |  |
| cg26861460 | 0.000386 | 0.34 | 0.05 | 0.14 | 0.04 | -0.20 | 64098 | *PARVG* | 1402 | FALSE |  |
| cg12072001 | 1.50E-05 | 0.40 | 0.06 | 0.20 | 0.03 | -0.20 | 10417 | *SPON2* | 170 | FALSE |  |
| cg22572779 | 4.62E-06 | 0.64 | 0.04 | 0.43 | 0.02 | -0.21 | 221718 | *MGC40222* | 13 | FALSE |  |
| cg05626013 | 0.013863 | 0.36 | 0.11 | 0.15 | 0.02 | -0.21 | 347731 | *LRRTM3* | 561 | FALSE |  |
| cg04237003 | 0.000949 | 0.65 | 0.09 | 0.45 | 0.06 | -0.21 | 745 | *C11orf9* | 859 | FALSE |  |
| cg00795812 | 0.000502 | 0.41 | 0.04 | 0.21 | 0.09 | -0.21 | 5133 | *PDCD1* | 951 | TRUE | 2:242450515-242450972 |
| cg17504145 | 1.81E-06 | 0.77 | 0.02 | 0.56 | 0.04 | -0.21 | 1415 | *CRYBB2* | 156 | FALSE |  |
| cg03872376 | 6.39E-06 | 0.83 | 0.01 | 0.63 | 0.07 | -0.21 | 57829 | *ZP4* | 107 | FALSE |  |
| cg11492040 | 1.02E-06 | 0.66 | 0.03 | 0.45 | 0.02 | -0.21 | 283120 | *H19* | NA | TRUE | 11:1973035-1973716 |
| cg24877842 | 0.000347 | 0.29 | 0.06 | 0.08 | 0.05 | -0.21 | 22906 | *TRAK1* | 233 | FALSE |  |
| cg02512860 | 0.000104 | 0.44 | 0.05 | 0.23 | 0.05 | -0.21 | 24146 | *CLDN15* | 365 | FALSE |  |
| cg21519900 | 0.003765 | 0.45 | 0.06 | 0.24 | 0.06 | -0.21 | 149954 | *C20orf186* | 143 | FALSE |  |
| cg08914623 | 6.68E-06 | 0.29 | 0.04 | 0.08 | 0.03 | -0.21 | 60529 | *ALX4* | NA | TRUE | 11:44283505-44284554 |
| cg10691387 | 2.80E-05 | 0.60 | 0.05 | 0.39 | 0.05 | -0.21 | 389123 | *IQCF2* | 27 | FALSE |  |
| cg04143809 | 0.00094 | 0.54 | 0.07 | 0.32 | 0.07 | -0.21 | 151258 | *FLJ39822* | 287 | TRUE | 2:165520387-165520601 |
| cg24169915 | 0.002195 | 0.48 | 0.06 | 0.26 | 0.05 | -0.21 | 283598 | *FLJ25773* | 37 | FALSE |  |
| cg01724150 | 0.004663 | 0.51 | 0.07 | 0.29 | 0.08 | -0.21 | 349565 | *NMNAT3* | 523 | FALSE |  |
| cg18613421 | 1.79E-06 | 0.43 | 0.05 | 0.21 | 0.02 | -0.21 | 256764 | *WDR72* | 316 | TRUE | 15:51838801-51839445 |
| cg06896207 | 0.001323 | 0.63 | 0.03 | 0.42 | 0.06 | -0.21 | 126308 | *MOBKL2A* | 905 | TRUE | 19:2046287-2048579 |
| cg17833578 | 8.81E-06 | 0.58 | 0.04 | 0.36 | 0.06 | -0.22 | 26103 | *LRRC21* | 343 | FALSE |  |
| cg22709192 | 5.28E-07 | 0.27 | 0.02 | 0.05 | 0.02 | -0.22 | 3227 | *HOXC11* | 20 | TRUE | 12:52652893-52656105 |
| cg16495265 | 1.15E-08 | 0.34 | 0.03 | 0.12 | 0.01 | -0.22 | 3215 | *HOXB5* | 108 | TRUE | 17:44025432-44027432 |
| cg12274479 | 0.002376 | 0.64 | 0.10 | 0.42 | 0.10 | -0.22 | 3339 | *HSPG2* | 765 | TRUE | 1:22135901-22137310 |
| cg16682903 | 1.23E-06 | 0.39 | 0.03 | 0.17 | 0.04 | -0.22 | 90 | *ACVR1* | 120 | FALSE |  |
| cg14558138 | 1.91E-07 | 0.87 | 0.01 | 0.65 | 0.05 | -0.22 | 27063 | *ANKRD1* | 522 | FALSE |  |
| cg15783800 | 8.02E-06 | 0.71 | 0.02 | 0.49 | 0.02 | -0.22 | 115701 | *HAK* | 404 | TRUE | 18:54397786-54397992 |
| cg18727700 | 0.002005 | 0.44 | 0.10 | 0.21 | 0.07 | -0.23 | 27286 | *SRPX2* | 295 | FALSE |  |
| cg18292711 | 7.76E-07 | 0.56 | 0.03 | 0.33 | 0.04 | -0.23 | 3799 | *KIF5B* | 933 | TRUE | 10:32386051-32386257 |
| cg02989257 | 0.000233 | 0.56 | 0.04 | 0.33 | 0.06 | -0.23 | 1307 | *COL16A1* | 375 | FALSE |  |
| cg26215727 | 9.02E-07 | 0.37 | 0.04 | 0.14 | 0.02 | -0.23 | 6337 | *SCNN1A* | 822 | FALSE |  |
| cg03329572 | 5.50E-07 | 0.86 | 0.02 | 0.63 | 0.08 | -0.23 | 83416 | *FCRL5* | 943 | FALSE |  |
| cg11251877 | 0.000823 | 0.56 | 0.12 | 0.33 | 0.08 | -0.23 | 26236 | *C6orf54* | 1224 | FALSE |  |
| cg07546360 | 0.000451 | 0.59 | 0.07 | 0.36 | 0.05 | -0.23 | 400931 | *FLJ27365* | 140 | TRUE | 22:44860477-44860760 |
| cg04329382 | 0.000608 | 0.41 | 0.07 | 0.18 | 0.04 | -0.23 | 400931 | *FLJ27365* | 992 | TRUE | 22:44859540-44859922 |
| cg19560971 | 2.55E-05 | 0.56 | 0.06 | 0.33 | 0.06 | -0.23 | 83552 | *MFRP* | 868 | FALSE |  |
| cg13131015 | 0.000145 | 0.59 | 0.05 | 0.35 | 0.09 | -0.23 | 2984 | *GUCY2C* | 113 | FALSE |  |
| cg11481351 | 0.000885 | 0.77 | 0.04 | 0.54 | 0.10 | -0.23 | 163479 | *FNDC7* | 354 | FALSE |  |
| cg22901146 | 2.69E-05 | 0.76 | 0.05 | 0.53 | 0.09 | -0.23 | 2986 | *GUCY2F* | 178 | FALSE |  |
| cg05119218 | 0.000864 | 0.63 | 0.04 | 0.40 | 0.09 | -0.23 | 3101 | *HK3* | 1242 | FALSE |  |
| cg00718513 | 0.000629 | 0.60 | 0.07 | 0.37 | 0.05 | -0.23 | 28905 |  | 78 | FALSE |  |
| cg10584819 | 9.56E-06 | 0.59 | 0.04 | 0.35 | 0.01 | -0.23 | 3251 | *HPRT1* | 587 | TRUE | X:133421038-133422628 |
| cg15700739 | 0.008902 | 0.43 | 0.13 | 0.20 | 0.11 | -0.23 | 3222 | *HOXC5* | 868 | TRUE | 12:52712802-52715131 |
| cg15441973 | 6.95E-08 | 0.76 | 0.02 | 0.53 | 0.05 | -0.23 | 3176 | *HNMT* | 491 | FALSE |  |
| cg05335315 | 0.000423 | 0.54 | 0.06 | 0.31 | 0.03 | -0.23 | 3714 | *JAG2* | 547 | TRUE | 14:104704480-104706946 |
| cg13703437 | 0.000341 | 0.72 | 0.05 | 0.48 | 0.06 | -0.23 | 2533 | *FYB* | 31 | FALSE |  |
| cg18403361 | 0.01833 | 0.44 | 0.14 | 0.20 | 0.13 | -0.24 | 161198 | *CLEC14A* | 176 | TRUE | 14:37793841-37795561 |
| cg09272256 | 2.53E-05 | 0.55 | 0.07 | 0.31 | 0.07 | -0.24 | 1109 | *AKR1C4* | 83 | FALSE |  |
| cg03440267 | 1.96E-05 | 0.45 | 0.04 | 0.21 | 0.04 | -0.24 | 2162 | *F13A1* | 159 | FALSE |  |
| cg22181664 | 3.42E-08 | 0.61 | 0.04 | 0.36 | 0.03 | -0.25 | 1179 | *CLCA1* | 274 | FALSE |  |
| cg25025243 | 2.46E-05 | 0.49 | 0.04 | 0.24 | 0.06 | -0.25 | 2950 | *GSTP1* | 1052 | FALSE |  |
| cg12006284 | 2.08E-07 | 0.35 | 0.02 | 0.10 | 0.03 | -0.25 | 7490 | *WT1* | NA | TRUE | 11:32404590-32406255 |
| cg06516124 | 6.95E-08 | 0.29 | 0.04 | 0.04 | 0.01 | -0.25 | 7490 | *WT1* | NA | TRUE | 11:32406516-32407359 |
| cg08678755 | 3.45E-06 | 0.85 | 0.02 | 0.59 | 0.07 | -0.25 | 5169 | *ENPP3* | 1464 | FALSE |  |
| cg08872493 | 2.93E-05 | 0.43 | 0.08 | 0.18 | 0.05 | -0.25 | 3352 | *HTR1D* | 195 | FALSE |  |
| cg14646244 | 5.79E-06 | 0.57 | 0.03 | 0.31 | 0.02 | -0.25 | 5172 | *SLC26A4* | 793 | FALSE |  |
| cg04716261 | 1.96E-06 | 0.55 | 0.04 | 0.29 | 0.04 | -0.26 | 140625 | *ARPM2* | 67 | FALSE |  |
| cg07153965 | 1.38E-05 | 0.60 | 0.05 | 0.34 | 0.04 | -0.26 | 56 | *ACRV1* | 1145 | FALSE |  |
| cg06224510 | 3.21E-05 | 0.74 | 0.03 | 0.48 | 0.09 | -0.26 | 3698 | *ITIH2* | 218 | FALSE |  |
| cg14754581 | 1.33E-05 | 0.30 | 0.12 | 0.04 | 0.01 | -0.26 | 9034 | *CCRL2* | 32 | FALSE |  |
| cg09499849 | 2.05E-08 | 0.29 | 0.05 | 0.03 | 0.01 | -0.26 | 90 | *ACVR1* | 368 | FALSE |  |
| cg26245202 | 0.000653 | 0.49 | 0.06 | 0.22 | 0.05 | -0.26 | 2005 | *ELK4* | 864 | FALSE |  |
| cg13897449 | 0.016868 | 0.73 | 0.05 | 0.47 | 0.13 | -0.26 | 5207 | *PFKFB1* | 1331 | FALSE |  |
| cg07962315 | 0.000201 | 0.49 | 0.04 | 0.22 | 0.07 | -0.26 | 54981 | *C9orf95* | 1375 | FALSE |  |
| cg06550629 | 0.0011 | 0.63 | 0.09 | 0.36 | 0.08 | -0.26 | 283383 | *GPR133* | 335 | FALSE |  |
| cg05654163 | 8.49E-07 | 0.47 | 0.02 | 0.20 | 0.06 | -0.27 | 29986 | *SLC39A2* | 32 | FALSE |  |
| cg13633026 | 1.99E-06 | 0.60 | 0.06 | 0.33 | 0.04 | -0.27 | 83661 | *MS4A8B* | 474 | FALSE |  |
| cg25383242 | 6.41E-07 | 0.81 | 0.01 | 0.54 | 0.07 | -0.27 | 85445 | *CNTNAP4* | 86 | TRUE | 16:74901143-74901361 |
| cg15446391 | 1.63E-08 | 0.44 | 0.02 | 0.17 | 0.03 | -0.27 | 7490 | *WT1* | NA | TRUE | 11:32408563-32409903 |
| cg02124291 | 2.08E-07 | 0.77 | 0.02 | 0.50 | 0.08 | -0.27 | 26659 | *OR7A5* | 16 | FALSE |  |
| cg18321354 | 2.55E-05 | 0.78 | 0.06 | 0.51 | 0.08 | -0.27 | 84433 | *CARD11* | 1038 | FALSE |  |
| cg14898892 | 8.08E-05 | 0.47 | 0.08 | 0.20 | 0.05 | -0.27 | 57619 | *SHRM* | 73 | FALSE |  |
| cg02906939 | 1.09E-08 | 0.87 | 0.02 | 0.60 | 0.04 | -0.27 | 3176 | *HNMT* | 493 | FALSE |  |
| cg27177839 | 0.000354 | 0.43 | 0.11 | 0.16 | 0.02 | -0.27 | 3714 | *JAG2* | 608 | TRUE | 14:104704480-104706946 |
| cg10466917 | 7.60E-07 | 0.74 | 0.02 | 0.46 | 0.06 | -0.27 | 136541 | *TRY1* | 579 | FALSE |  |
| cg01318557 | 3.72E-05 | 0.55 | 0.05 | 0.28 | 0.01 | -0.27 | 7462 | *LAT2* | 44 | TRUE | 7:73261846-73262293 |
| cg05222924 | 5.68E-09 | 0.30 | 0.03 | 0.03 | 0.01 | -0.27 | 7490 | *WT1* | NA | TRUE | 11:32406516-32407359 |
| cg02022375 | 8.71E-05 | 0.65 | 0.06 | 0.38 | 0.07 | -0.27 | 81851 | *KRTAP1-1* | 8 | FALSE |  |
| cg15815843 | 3.38E-06 | 0.78 | 0.03 | 0.50 | 0.04 | -0.28 | 8076 | *MFAP5* | 125 | FALSE |  |
| cg18433694 | 3.76E-06 | 0.48 | 0.06 | 0.20 | 0.02 | -0.28 | 286410 | *ATP11C* | 124 | FALSE |  |
| cg20789824 | 1.36E-06 | 0.70 | 0.01 | 0.41 | 0.04 | -0.28 | 169611 | *OLFML2A* | 292 | FALSE |  |
| cg03005261 | 7.80E-07 | 0.40 | 0.04 | 0.12 | 0.03 | -0.29 | 55013 | *FLJ20647* | 589 | TRUE | 4:110700137-110701779 |
| cg19481052 | 2.80E-06 | 0.54 | 0.06 | 0.25 | 0.02 | -0.29 | 5354 | *PLP1* | 383 | FALSE |  |
| cg06259570 | 7.78E-06 | 0.66 | 0.02 | 0.37 | 0.04 | -0.29 | 64066 | *MMP27* | 374 | FALSE |  |
| cg23163573 | 6.35E-07 | 0.53 | 0.05 | 0.24 | 0.04 | -0.29 | 6819 | *SULT1C1* | 373 | FALSE |  |
| cg14440664 | 4.84E-05 | 0.57 | 0.06 | 0.28 | 0.06 | -0.30 | 80380 | *PDCD1LG2* | 928 | FALSE |  |
| cg12781568 | 0.000857 | 0.76 | 0.07 | 0.46 | 0.14 | -0.30 | 7490 | *WT1* | NA | TRUE | 11:32408563-32409903 |
| cg24754277 | 3.74E-07 | 0.51 | 0.07 | 0.20 | 0.02 | -0.31 | 1612 | *DAPK1* | NA | TRUE | 9:89301899-89304060 |
| cg10925082 | 0.000223 | 0.76 | 0.04 | 0.45 | 0.09 | -0.31 | 397 | *ARHGDIB* | 141 | FALSE |  |
| cg19170321 | 7.73E-09 | 0.85 | 0.01 | 0.54 | 0.04 | -0.31 | 825 | *CAPN3* | 15 | FALSE |  |
| cg02097420 | 1.14E-08 | 0.90 | 0.02 | 0.59 | 0.08 | -0.31 | 3273 | *HRG* | 76 | FALSE |  |
| cg23752985 | 1.44E-06 | 0.70 | 0.04 | 0.39 | 0.04 | -0.31 | 8673 | *VAMP8* | 1146 | FALSE |  |
| cg26866014 | 1.14E-08 | 0.80 | 0.02 | 0.49 | 0.06 | -0.32 | 202865 | *C7orf33* | 55 | FALSE |  |
| cg22627427 | 2.39E-05 | 0.86 | 0.08 | 0.55 | 0.07 | -0.32 | 745 | *C11orf9* | 363 | FALSE |  |
| cg21660392 | 0.005762 | 0.66 | 0.09 | 0.34 | 0.15 | -0.32 | 10351 | *ABCA8* | 988 | FALSE |  |
| cg24495017 | 0.000126 | 0.54 | 0.12 | 0.22 | 0.06 | -0.32 | 5307 | *PITX1* | 542 | TRUE | 5:134396353-134398530 |
| cg00509670 | 8.03E-08 | 0.54 | 0.05 | 0.22 | 0.05 | -0.32 | 5083 | *PAX9* | 592 | TRUE | 14:36199815-36200327 |
| cg19881895 | 0.000104 | 0.76 | 0.04 | 0.43 | 0.07 | -0.32 | 29015 | *SLC43A3* | 487 | FALSE |  |
| cg18003231 | 3.88E-06 | 0.66 | 0.06 | 0.32 | 0.05 | -0.33 | 83733 | *SLC25A18* | 562 | TRUE | 22:16423630-16423844 |
| cg16739580 | 2.71E-07 | 0.81 | 0.03 | 0.48 | 0.06 | -0.33 | 152138 | *POP2* | 122 | FALSE |  |
| cg22416721 | 5.62E-06 | 0.77 | 0.08 | 0.43 | 0.05 | -0.34 | 1758 | *DMP1* | 885 | FALSE |  |
| cg05600174 | 7.80E-05 | 0.48 | 0.08 | 0.14 | 0.04 | -0.34 | 54981 | *C9orf95* | 1439 | FALSE |  |
| cg16501028 | 5.31E-06 | 0.61 | 0.02 | 0.27 | 0.05 | -0.34 | 7490 | *WT1* | NA | TRUE | 11:32406516-32407359 |
| cg18216249 | 0.000267 | 0.57 | 0.08 | 0.22 | 0.04 | -0.35 | 79657 | *FLJ21908* | 1031 | FALSE |  |
| cg26620157 | 3.03E-06 | 0.56 | 0.05 | 0.22 | 0.09 | -0.35 | 5083 | *PAX9* | 608 | TRUE | 14:36199815-36200327 |
| cg24030609 | 1.03E-05 | 0.67 | 0.08 | 0.32 | 0.05 | -0.35 | 51032 | *ELA2B* | 15 | FALSE |  |
| cg04456238 | 1.63E-08 | 0.42 | 0.02 | 0.07 | 0.03 | -0.35 | 7490 | *WT1* | NA | TRUE | 11:32406516-32407359 |
| cg08090640 | 0.000801 | 0.59 | 0.11 | 0.24 | 0.05 | -0.35 | 3430 | *IFI35* | 464 | FALSE |  |
| cg04457051 | 2.62E-06 | 0.60 | 0.07 | 0.25 | 0.06 | -0.35 | 60592 | *SCOC* | 29 | FALSE |  |
| cg06338119 | 1.11E-07 | 0.61 | 0.05 | 0.25 | 0.04 | -0.35 | 57529 | *RGAG1* | 423 | FALSE |  |
| cg01671575 | 3.21E-08 | 0.88 | 0.01 | 0.52 | 0.06 | -0.36 | 79605 | *PGBD5* | 199 | FALSE |  |
| cg22199118 | 7.76E-07 | 0.62 | 0.08 | 0.27 | 0.04 | -0.36 | 116328 | *C8orf34* | 779 | FALSE |  |
| cg06690548 | 1.64E-06 | 0.84 | 0.03 | 0.48 | 0.04 | -0.36 | 23657 | *SLC7A11* | 415 | TRUE | 4:139382255-139382463 |
| cg09432376 | 1.36E-06 | 0.59 | 0.08 | 0.23 | 0.03 | -0.36 | 80830 | *APOL6* | 198 | FALSE |  |
| cg01693350 | 1.14E-08 | 0.55 | 0.03 | 0.19 | 0.05 | -0.36 | 7490 | *WT1* | NA | TRUE | 11:32408563-32409903 |
| cg09390792 | 4.98E-08 | 0.81 | 0.05 | 0.44 | 0.07 | -0.37 | 90139 | *TSPAN18* | 392 | FALSE |  |
| cg18878432 | 2.64E-06 | 0.57 | 0.02 | 0.18 | 0.06 | -0.39 | 3216 | *HOXB6* | 1075 | TRUE | 17:44038335-44038975 |
| cg16077929 | 8.64E-07 | 0.69 | 0.05 | 0.30 | 0.06 | -0.39 | 8814 | *CDKL1* | 108 | FALSE |  |
| cg13641903 | 1.69E-08 | 0.64 | 0.03 | 0.25 | 0.06 | -0.39 | 7490 | *WT1* | NA | TRUE | 11:32408563-32409903 |
| cg06256735 | 3.39E-06 | 0.70 | 0.06 | 0.30 | 0.08 | -0.39 | 8076 | *MFAP5* | 461 | FALSE |  |
| cg15976539 | 2.45E-08 | 0.78 | 0.03 | 0.38 | 0.04 | -0.40 | 147429 | *C18orf16* | 46 | FALSE |  |
| cg13492227 | 4.27E-07 | 0.54 | 0.11 | 0.14 | 0.03 | -0.40 | 2256 | *FGF11* | 1253 | TRUE | 17:7281849-7282696 |
| cg17939444 | 1.13E-07 | 0.70 | 0.05 | 0.30 | 0.07 | -0.40 | 56892 | *C8orf4* | 546 | FALSE |  |
| cg00030047 | 4.79E-08 | 0.60 | 0.05 | 0.18 | 0.04 | -0.42 | 148646 | *C1orf188* | 130 | TRUE | 1:6190892-6192286 |
| cg23254045 | 6.29E-08 | 0.68 | 0.08 | 0.24 | 0.05 | -0.44 | 145942 | *TMCO5* | 1003 | FALSE |  |
| cg16463460 | 2.08E-07 | 0.71 | 0.02 | 0.26 | 0.05 | -0.44 | 7490 | *WT1* | NA | TRUE | 11:32411271-32413831 |
| cg11391732 | 3.43E-07 | 0.86 | 0.01 | 0.39 | 0.07 | -0.46 | 8988 | *HSPB3* | 474 | TRUE | 5:53787529-53787747 |
| cg05885720 | 1.00E-07 | 0.69 | 0.05 | 0.21 | 0.07 | -0.48 | 2012 | *EMP1* | 1228 | FALSE |  |
| cg00729275 | 1.61E-08 | 0.74 | 0.06 | 0.25 | 0.06 | -0.49 | 147429 | *C18orf16* | 138 | FALSE |  |
| cg15731815 | 1.45E-08 | 0.59 | 0.05 | 0.07 | 0.04 | -0.51 | 148646 | *C1orf188* | 340 | TRUE | 1:6190892-6192286 |
| cg03852144 | 3.92E-09 | 0.57 | 0.11 | 0.06 | 0.01 | -0.51 | 2745 | *GLRX* | 1258 | FALSE |  |
| cg06378617 | 1.64E-09 | 0.69 | 0.05 | 0.14 | 0.04 | -0.55 | 25984 | *KRT23* | 1305 | FALSE |  |
| cg25782229 | 2.93E-08 | 0.72 | 0.02 | 0.09 | 0.01 | -0.63 | 7490 | *WT1* | NA | TRUE | 11:32406516-32407359 |
