# Supplementary material for: A Comparison of Genome-Wide DNA Methylation Patterns between Different Vascular Tissues from Patients with Coronary Heart Disease
Source: PLoS One. 2015 Apr 9;10(4):e0122601. doi: 10.1371/journal.pone.0122601 (PMC4391864; doi:10.1371/journal.pone.0122601)
Supplement: S4 Table — The results for each enriched GO category (biological process) are listed in the table. C—number of reference genes in the category; O—number of genes in the gene set and also in the category, E—expected number in the category, R—Ratio of enrichment, rawP—p value from hypergeometric test, and adjP—p value adjusted by the multiple test adjustment. (DOCX) [file pone.0122601.s004.docx]

**APPENDIX TABLE 6**

**WebGestalt-generated significant ontologies for hypermethylated CpG-sites in GSV**

| **biological process regulation of multicellular organismal development**  **GO:2000026**  **C=1005;O=33;E=12.35;R=2.67;rawP=1.26e-07;adjP=0.0002** | | |
| --- | --- | --- |
| Entrez ID | Gene symbol | Description |
| 50964 | *SOST* | sclerostin |
| 27329 | *ANGPTL3* | angiopoietin-like 3 |
| 5552 | *SRGN* | serglycin |
| 6929 | *TCF3* | transcription factor 3 (E2A immunoglobulin enhancer binding factors E12/E47) |
| 9745 | *ZNF536* | zinc finger protein 536 |
| 358 | *AQP1* | aquaporin 1 (Colton blood group) |
| 23213 | *SULF1* | sulfatase 1 |
| 3207 | *HOXA11* | homeobox A11 |
| 4908 | *NTF3* | neurotrophin 3 |
| 2152 | *F3* | coagulation factor III (thromboplastin, tissue factor) |
| 2202 | *EFEMP1* | EGF containing fibulin-like extracellular matrix protein 1 |
| 5728 | *PTEN* | phosphatase and tensin homolog |
| 6935 | *ZEB1* | zinc finger E-box binding homeobox 1 |
| 153090 | *DAB2IP* | DAB2 interacting protein |
| 6348 | *CCL3* | chemokine (C-C motif) ligand 3 |
| 10911 | *UTS2* | urotensin 2 |
| 5029 | *P2RY2* | purinergic receptor P2Y, G-protein coupled, 2 |
| 4487 | *MSX1* | msh homeobox 1 |
| 6615 | *SNAI1* | snail homolog 1 (Drosophila) |
| 2011 | *MARK2* | MAP/microtubule affinity-regulating kinase 2 |
| 3213 | *HOXB3* | homeobox B3 |
| 5788 | *PTPRC* | protein tyrosine phosphatase, receptor type, C |
| 10677 | *AVIL* | advillin |
| 2670 | *GFAP* | glial fibrillary acidic protein |
| 3199 | *HOXA2* | homeobox A2 |
| 3202 | *HOXA5* | homeobox A5 |
| 199699 | *DAND5* | DAN domain family, member 5 |
| 7038 | *TG* | thyroglobulin |
| 861 | *RUNX1* | runt-related transcription factor 1 |
| 940 | *CD28* | CD28 molecule |
| 5947 | *RBP1* | retinol binding protein 1, cellular |
| 54583 | *EGLN1* | egl nine homolog 1 (C. elegans) |
| 25791 | *NGEF* | neuronal guanine nucleotide exchange factor |
| **biological process regulation of developmental process**  **GO:0050793**  **C=1283;O=37;E=15.77;R=2.35;rawP=4.91e-07;adjP=0.0003** | | |
| Entrez ID | Gene symbol | Description |
| 50964 | *SOST* | sclerostin |
| 27329 | *ANGPTL3* | angiopoietin-like 3 |
| 122953 | *JDP2* | Jun dimerization protein 2 |
| 5552 | *SRGN* | serglycin |
| 6929 | *TCF3* | transcription factor 3 (E2A immunoglobulin enhancer binding factors E12/E47) |
| 9745 | *ZNF536* | zinc finger protein 536 |
| 358 | *AQP1* | aquaporin 1 (Colton blood group) |
| 23213 | *SULF1* | sulfatase 1 |
| 3207 | *HOXA11* | homeobox A11 |
| 4908 | *NTF3* | neurotrophin 3 |
| 2152 | *F3* | coagulation factor III (thromboplastin, tissue factor) |
| 2202 | *EFEMP1* | EGF containing fibulin-like extracellular matrix protein 1 |
| 5728 | *PTEN* | phosphatase and tensin homolog |
| 9350 | *CER1* | cerberus 1, cysteine knot superfamily, homolog (Xenopus laevis) |
| 6935 | *ZEB1* | zinc finger E-box binding homeobox 1 |
| 153090 | *DAB2IP* | DAB2 interacting protein |
| 6348 | *CCL3* | chemokine (C-C motif) ligand 3 |
| 10911 | *UTS2* | urotensin 2 |
| 5029 | *P2RY2* | purinergic receptor P2Y, G-protein coupled, 2 |
| 4487 | *MSX1* | msh homeobox 1 |
| 6615 | *SNAI1* | snail homolog 1 (Drosophila) |
| 252995 | *FNDC5* | fibronectin type III domain containing 5 |
| 2011 | *MARK2* | MAP/microtubule affinity-regulating kinase 2 |
| 3213 | *HOXB3* | homeobox B3 |
| 5788 | *PTPRC* | protein tyrosine phosphatase, receptor type, C |
| 10677 | *AVIL* | advillin |
| 2670 | *GFAP* | glial fibrillary acidic protein |
| 3199 | *HOXA2* | homeobox A2 |
| 3202 | *HOXA5* | homeobox A5 |
| 199699 | *DAND5* | DAN domain family, member 5 |
| 7038 | *TG* | thyroglobulin |
| 1749 | *DLX5* | distal-less homeobox 5 |
| 861 | *RUNX1* | runt-related transcription factor 1 |
| 940 | *CD28* | CD28 molecule |
| 5947 | *RBP1* | retinol binding protein 1, cellular |
| 25791 | *NGEF* | neuronal guanine nucleotide exchange factor |
| 54583 | *EGLN1* | egl nine homolog 1 (C. elegans) |
| **biological process regulation of multicellular organismal process**  **GO:0051239**  **C=1614;O=41;E=19.84;R=2.07;rawP=2.80e-06;adjP=0.0013** | | |
| Entrez ID | Gene symbol | Description |
| 50964 | *SOST* | sclerostin |
| 27329 | *ANGPTL3* | angiopoietin-like 3 |
| 5024 | *P2RX3* | purinergic receptor P2X, ligand-gated ion channel, 3 |
| 5552 | *SRGN* | serglycin |
| 6929 | *TCF3* | transcription factor 3 (E2A immunoglobulin enhancer binding factors E12/E47) |
| 9745 | *ZNF536* | zinc finger protein 536 |
| 358 | *AQP1* | aquaporin 1 (Colton blood group) |
| 23213 | *SULF1* | sulfatase 1 |
| 3207 | *HOXA11* | homeobox A11 |
| 4908 | *NTF3* | neurotrophin 3 |
| 2152 | *F3* | coagulation factor III (thromboplastin, tissue factor) |
| 2202 | *EFEMP1* | EGF containing fibulin-like extracellular matrix protein 1 |
| 5728 | *PTEN* | phosphatase and tensin homolog |
| 6935 | *ZEB1* | zinc finger E-box binding homeobox 1 |
| 153090 | *DAB2IP* | DAB2 interacting protein |
| 6348 | *CCL3* | chemokine (C-C motif) ligand 3 |
| 10911 | *UTS2* | urotensin 2 |
| 5029 | *P2RY2* | purinergic receptor P2Y, G-protein coupled, 2 |
| 7068 | *THRB* | thyroid hormone receptor, beta |
| 4487 | *MSX1* | msh homeobox 1 |
| 6615 | *SNAI1* | snail homolog 1 (Drosophila) |
| 1240 | *CMKLR1* | chemokine-like receptor 1 |
| 10203 | *CALCRL* | calcitonin receptor-like |
| 3578 | *IL9* | interleukin 9 |
| 1991 | *ELANE* | elastase, neutrophil expressed |
| 4160 | *MC4R* | melanocortin 4 receptor |
| 2011 | *MARK2* | MAP/microtubule affinity-regulating kinase 2 |
| 3213 | *HOXB3* | homeobox B3 |
| 5788 | *PTPRC* | protein tyrosine phosphatase, receptor type, C |
| 2670 | *GFAP* | glial fibrillary acidic protein |
| 10677 | *AVIL* | advillin |
| 3199 | *HOXA2* | homeobox A2 |
| 3202 | *HOXA5* | homeobox A5 |
| 199699 | *DAND5* | DAN domain family, member 5 |
| 7038 | *TG* | thyroglobulin |
| 1749 | *DLX5* | distal-less homeobox 5 |
| 861 | *RUNX1* | runt-related transcription factor 1 |
| 940 | *CD28* | CD28 molecule |
| 5947 | *RBP1* | retinol binding protein 1, cellular |
| 25791 | *NGEF* | neuronal guanine nucleotide exchange factor |
| 54583 | *EGLN1* | egl nine homolog 1 (C. elegans) |
| **biological process skeletal system development**  **GO:0001501**  **C=356;O=16;E=4.38;R=3.66;rawP=7.59e-06;adjP=0.0026** | | |
| Entrez ID | Gene symbol | Description |
| 5788 | *PTPRC* | protein tyrosine phosphatase, receptor type, C |
| 2202 | *EFEMP1* | EGF containing fibulin-like extracellular matrix protein 1 |
| 4487 | *MSX1* | msh homeobox 1 |
| 60529 | *ALX4* | ALX homeobox 4 |
| 6615 | *SNAI1* | snail homolog 1 (Drosophila) |
| 2251 | *FGF6* | fibroblast growth factor 6 |
| 3199 | *HOXA2* | homeobox A2 |
| 3202 | *HOXA5* | homeobox A5 |
| 1240 | *CMKLR1* | chemokine-like receptor 1 |
| 23213 | *SULF1* | sulfatase 1 |
| 9350 | *CER1* | cerberus 1, cysteine knot superfamily, homolog (Xenopus laevis) |
| 1749 | *DLX5* | distal-less homeobox 5 |
| 861 | *RUNX1* | runt-related transcription factor 1 |
| 3207 | *HOXA11* | homeobox A11 |
| 2300 | *FOXL1* | forkhead box L1 |
| 3213 | *HOXB3* | homeobox B3 |
| **biological process cartilage development**  **GO:0051216**  **C=150;O=10;E=1.84;R=5.42;rawP=1.56e-05;adjP=0.0042** | | |
| Entrez ID | Gene symbol | Description |
| 2202 | *EFEMP1* | EGF containing fibulin-like extracellular matrix protein 1 |
| 4487 | *MSX1* | msh homeobox 1 |
| 6615 | *SNAI1* | snail homolog 1 (Drosophila) |
| 2251 | *FGF6* | fibroblast growth factor 6 |
| 3202 | *HOXA5* | homeobox A5 |
| 23213 | *SULF1* | sulfatase 1 |
| 9350 | *CER1* | cerberus 1, cysteine knot superfamily, homolog (Xenopus laevis) |
| 3207 | *HOXA11* | homeobox A11 |
| 2300 | *FOXL1* | forkhead box L1 |
| 3213 | *HOXB3* | homeobox B3 |
| **biological process single-multicellular organism process**  **GO:0044707**  **C=4918;O=85;E=60.46;R=1.41;rawP=2.68e-05;adjP=0.0061** | | |
| Entrez ID | Gene symbol | Description |
| 3053 | *SERPIND1* | serpin peptidase inhibitor, clade D (heparin cofactor), member 1 |
| 2206 | *MS4A2* | membrane-spanning 4-domains, subfamily A, member 2 |
| 358 | *AQP1* | aquaporin 1 (Colton blood group) |
| 5407 | *PNLIPRP1* | pancreatic lipase-related protein 1 |
| 4908 | *NTF3* | neurotrophin 3 |
| 433 | *ASGR2* | asialoglycoprotein receptor 2 |
| 2152 | *F3* | coagulation factor III (thromboplastin, tissue factor) |
| 108 | *ADCY2* | adenylate cyclase 2 (brain) |
| 81285 | *OR51E2* | olfactory receptor, family 51, subfamily E, member 2 |
| 220 | *ALDH1A3* | aldehyde dehydrogenase 1 family, member A3 |
| 9350 | *CER1* | cerberus 1, cysteine knot superfamily, homolog (Xenopus laevis) |
| 6935 | *ZEB1* | zinc finger E-box binding homeobox 1 |
| 153090 | *DAB2IP* | DAB2 interacting protein |
| 6348 | *CCL3* | chemokine (C-C motif) ligand 3 |
| 7068 | *THRB* | thyroid hormone receptor, beta |
| 6954 | *TCP11* | t-complex 11, testis-specific |
| 2251 | *FGF6* | fibroblast growth factor 6 |
| 1240 | *CMKLR1* | chemokine-like receptor 1 |
| 2815 | *GP9* | glycoprotein IX (platelet) |
| 4773 | *NFATC2* | nuclear factor of activated T-cells, cytoplasmic, calcineurin-dependent 2 |
| 3578 | *IL9* | interleukin 9 |
| 4160 | *MC4R* | melanocortin 4 receptor |
| 79652 | *TMEM204* | transmembrane protein 204 |
| 6540 | *SLC6A13* | solute carrier family 6 (neurotransmitter transporter, GABA), member 13 |
| 3641 | *INSL4* | insulin-like 4 (placenta) |
| 9177 | *HTR3B* | 5-hydroxytryptamine (serotonin) receptor 3B, ionotropic |
| 6927 | *HNF1A* | HNF1 homeobox A |
| 2670 | *GFAP* | glial fibrillary acidic protein |
| 115111 | *SLC26A7* | solute carrier family 26, member 7 |
| 60529 | *ALX4* | ALX homeobox 4 |
| 3199 | *HOXA2* | homeobox A2 |
| 199699 | *DAND5* | DAN domain family, member 5 |
| 1749 | *DLX5* | distal-less homeobox 5 |
| 861 | *RUNX1* | runt-related transcription factor 1 |
| 5947 | *RBP1* | retinol binding protein 1, cellular |
| 54463 | *FAM134B* | family with sequence similarity 134, member B |
| 5047 | *PAEP* | progestagen-associated endometrial protein |
| 7033 | *TFF3* | trefoil factor 3 (intestinal) |
| 50964 | *SOST* | sclerostin |
| 5024 | *P2RX3* | purinergic receptor P2X, ligand-gated ion channel, 3 |
| 27329 | *ANGPTL3* | angiopoietin-like 3 |
| 4632 | *MYL1* | myosin, light chain 1, alkali; skeletal, fast |
| 6929 | *TCF3* | transcription factor 3 (E2A immunoglobulin enhancer binding factors E12/E47) |
| 5552 | *SRGN* | serglycin |
| 9745 | *ZNF536* | zinc finger protein 536 |
| 23213 | *SULF1* | sulfatase 1 |
| 5493 | *PPL* | periplakin |
| 55224 | *ETNK2* | ethanolamine kinase 2 |
| 3207 | *HOXA11* | homeobox A11 |
| 9514 | *GAL3ST1* | galactose-3-O-sulfotransferase 1 |
| 6414 | *SEPP1* | selenoprotein P, plasma, 1 |
| 2202 | *EFEMP1* | EGF containing fibulin-like extracellular matrix protein 1 |
| 56913 | *C1GALT1* | core 1 synthase, glycoprotein-N-acetylgalactosamine 3-beta-galactosyltransferase, 1 |
| 5133 | *PDCD1* | programmed cell death 1 |
| 57549 | *IGSF9* | immunoglobulin superfamily, member 9 |
| 5728 | *PTEN* | phosphatase and tensin homolog |
| 23426 | *GRIP1* | glutamate receptor interacting protein 1 |
| 8871 | *SYNJ2* | synaptojanin 2 |
| 10911 | *UTS2* | urotensin 2 |
| 1586 | *CYP17A1* | cytochrome P450, family 17, subfamily A, polypeptide 1 |
| 5029 | *P2RY2* | purinergic receptor P2Y, G-protein coupled, 2 |
| 646 | *BNC1* | basonuclin 1 |
| 4487 | *MSX1* | msh homeobox 1 |
| 10678 | *B3GNT2* | UDP-GlcNAc:betaGal beta-1,3-N-acetylglucosaminyltransferase 2 |
| 962 | *CD48* | CD48 molecule |
| 6615 | *SNAI1* | snail homolog 1 (Drosophila) |
| 5739 | *PTGIR* | prostaglandin I2 (prostacyclin) receptor (IP) |
| 1271 | *CNTFR* | ciliary neurotrophic factor receptor |
| 10203 | *CALCRL* | calcitonin receptor-like |
| 1991 | *ELANE* | elastase, neutrophil expressed |
| 2011 | *MARK2* | MAP/microtubule affinity-regulating kinase 2 |
| 2300 | *FOXL1* | forkhead box L1 |
| 3213 | *HOXB3* | homeobox B3 |
| 5788 | *PTPRC* | protein tyrosine phosphatase, receptor type, C |
| 10677 | *AVIL* | advillin |
| 7547 | *ZIC3* | Zic family member 3 |
| 4986 | *OPRK1* | opioid receptor, kappa 1 |
| 27122 | *DKK3* | dickkopf 3 homolog (Xenopus laevis) |
| 3202 | *HOXA5* | homeobox A5 |
| 7038 | *TG* | thyroglobulin |
| 145226 | *RDH12* | retinol dehydrogenase 12 (all-trans/9-cis/11-cis) |
| 5026 | *P2RX5* | purinergic receptor P2X, ligand-gated ion channel, 5 |
| 940 | *CD28* | CD28 molecule |
| 25791 | *NGEF* | neuronal guanine nucleotide exchange factor |
| 54583 | *EGLN1* | egl nine homolog 1 (C. elegans) |
| **biological process multicellular organismal process**  **GO:0032501**  **C=4945;O=85;E=60.79;R=1.40;rawP=3.41e-05;adjP=0.0062** | | |
| Entrez ID | Gene symbol | Description |
| 3053 | *SERPIND1* | serpin peptidase inhibitor, clade D (heparin cofactor), member 1 |
| 2206 | *MS4A2* | membrane-spanning 4-domains, subfamily A, member 2 |
| 358 | *AQP1* | aquaporin 1 (Colton blood group) |
| 5407 | *PNLIPRP1* | pancreatic lipase-related protein 1 |
| 4908 | *NTF3* | neurotrophin 3 |
| 433 | *ASGR2* | asialoglycoprotein receptor 2 |
| 2152 | *F3* | coagulation factor III (thromboplastin, tissue factor) |
| 108 | *ADCY2* | adenylate cyclase 2 (brain) |
| 81285 | *OR51E2* | olfactory receptor, family 51, subfamily E, member 2 |
| 220 | *ALDH1A3* | aldehyde dehydrogenase 1 family, member A3 |
| 9350 | *CER1* | cerberus 1, cysteine knot superfamily, homolog (Xenopus laevis) |
| 6935 | *ZEB1* | zinc finger E-box binding homeobox 1 |
| 153090 | *DAB2IP* | DAB2 interacting protein |
| 6348 | *CCL3* | chemokine (C-C motif) ligand 3 |
| 7068 | *THRB* | thyroid hormone receptor, beta |
| 6954 | *TCP11* | t-complex 11, testis-specific |
| 2251 | *FGF6* | fibroblast growth factor 6 |
| 1240 | *CMKLR1* | chemokine-like receptor 1 |
| 2815 | *GP9* | glycoprotein IX (platelet) |
| 4773 | *NFATC2* | nuclear factor of activated T-cells, cytoplasmic, calcineurin-dependent 2 |
| 3578 | *IL9* | interleukin 9 |
| 4160 | *MC4R* | melanocortin 4 receptor |
| 79652 | *TMEM204* | transmembrane protein 204 |
| 6540 | *SLC6A13* | solute carrier family 6 (neurotransmitter transporter, GABA), member 13 |
| 3641 | *INSL4* | insulin-like 4 (placenta) |
| 9177 | *HTR3B* | 5-hydroxytryptamine (serotonin) receptor 3B, ionotropic |
| 6927 | *HNF1A* | HNF1 homeobox A |
| 2670 | *GFAP* | glial fibrillary acidic protein |
| 115111 | *SLC26A7* | solute carrier family 26, member 7 |
| 60529 | *ALX4* | ALX homeobox 4 |
| 3199 | *HOXA2* | homeobox A2 |
| 199699 | *DAND5* | DAN domain family, member 5 |
| 1749 | *DLX5* | distal-less homeobox 5 |
| 861 | *RUNX1* | runt-related transcription factor 1 |
| 5947 | *RBP1* | retinol binding protein 1, cellular |
| 54463 | *FAM134B* | family with sequence similarity 134, member B |
| 5047 | *PAEP* | progestagen-associated endometrial protein |
| 7033 | *TFF3* | trefoil factor 3 (intestinal) |
| 50964 | *SOST* | sclerostin |
| 5024 | *P2RX3* | purinergic receptor P2X, ligand-gated ion channel, 3 |
| 27329 | *ANGPTL3* | angiopoietin-like 3 |
| 4632 | *MYL1* | myosin, light chain 1, alkali; skeletal, fast |
| 6929 | *TCF3* | transcription factor 3 (E2A immunoglobulin enhancer binding factors E12/E47) |
| 5552 | *SRGN* | serglycin |
| 9745 | *ZNF536* | zinc finger protein 536 |
| 23213 | *SULF1* | sulfatase 1 |
| 5493 | *PPL* | periplakin |
| 55224 | *ETNK2* | ethanolamine kinase 2 |
| 3207 | *HOXA11* | homeobox A11 |
| 9514 | *GAL3ST1* | galactose-3-O-sulfotransferase 1 |
| 6414 | *SEPP1* | selenoprotein P, plasma, 1 |
| 2202 | *EFEMP1* | EGF containing fibulin-like extracellular matrix protein 1 |
| 56913 | *C1GALT1* | core 1 synthase, glycoprotein-N-acetylgalactosamine 3-beta-galactosyltransferase, 1 |
| 5133 | *PDCD1* | programmed cell death 1 |
| 57549 | *IGSF9* | immunoglobulin superfamily, member 9 |
| 5728 | *PTEN* | phosphatase and tensin homolog |
| 23426 | *GRIP1* | glutamate receptor interacting protein 1 |
| 8871 | *SYNJ2* | synaptojanin 2 |
| 10911 | *UTS2* | urotensin 2 |
| 1586 | *CYP17A1* | cytochrome P450, family 17, subfamily A, polypeptide 1 |
| 5029 | *P2RY2* | purinergic receptor P2Y, G-protein coupled, 2 |
| 646 | *BNC1* | basonuclin 1 |
| 4487 | *MSX1* | msh homeobox 1 |
| 10678 | *B3GNT2* | UDP-GlcNAc:betaGal beta-1,3-N-acetylglucosaminyltransferase 2 |
| 962 | *CD48* | CD48 molecule |
| 6615 | *SNAI1* | snail homolog 1 (Drosophila) |
| 5739 | *PTGIR* | prostaglandin I2 (prostacyclin) receptor (IP) |
| 1271 | *CNTFR* | ciliary neurotrophic factor receptor |
| 10203 | *CALCRL* | calcitonin receptor-like |
| 1991 | *ELANE* | elastase, neutrophil expressed |
| 2011 | *MARK2* | MAP/microtubule affinity-regulating kinase 2 |
| 2300 | *FOXL1* | forkhead box L1 |
| 3213 | *HOXB3* | homeobox B3 |
| 5788 | *PTPRC* | protein tyrosine phosphatase, receptor type, C |
| 10677 | *AVIL* | advillin |
| 7547 | *ZIC3* | Zic family member 3 |
| 4986 | *OPRK1* | opioid receptor, kappa 1 |
| 27122 | *DKK3* | dickkopf 3 homolog (Xenopus laevis) |
| 3202 | *HOXA5* | homeobox A5 |
| 7038 | *TG* | thyroglobulin |
| 145226 | *RDH12* | retinol dehydrogenase 12 (all-trans/9-cis/11-cis) |
| 5026 | *P2RX5* | purinergic receptor P2X, ligand-gated ion channel, 5 |
| 940 | *CD28* | CD28 molecule |
| 25791 | *NGEF* | neuronal guanine nucleotide exchange factor |
| 54583 | *EGLN1* | egl nine homolog 1 (C. elegans) |
| **biological process regulation of cell differentiation**  **GO:0045595**  **C=904;O=26;E=11.11;R=2.34;rawP=3.67e-05;adjP=0.0062** | | |
| Entrez ID | Gene symbol | Description |
| 4487 | *MSX1* | msh homeobox 1 |
| 6615 | *SNAI1* | snail homolog 1 (Drosophila) |
| 122953 | *JDP2* | Jun dimerization protein 2 |
| 6929 | *TCF3* | transcription factor 3 (E2A immunoglobulin enhancer binding factors E12/E47) |
| 9745 | *ZNF536* | zinc finger protein 536 |
| 3207 | *HOXA11* | homeobox A11 |
| 4908 | *NTF3* | neurotrophin 3 |
| 252995 | *FNDC5* | fibronectin type III domain containing 5 |
| 2011 | *MARK2* | MAP/microtubule affinity-regulating kinase 2 |
| 3213 | *HOXB3* | homeobox B3 |
| 5788 | *PTPRC* | protein tyrosine phosphatase, receptor type, C |
| 2202 | *EFEMP1* | EGF containing fibulin-like extracellular matrix protein 1 |
| 2670 | *GFAP* | glial fibrillary acidic protein |
| 10677 | *AVIL* | advillin |
| 5728 | *PTEN* | phosphatase and tensin homolog |
| 3199 | *HOXA2* | homeobox A2 |
| 3202 | *HOXA5* | homeobox A5 |
| 6935 | *ZEB1* | zinc finger E-box binding homeobox 1 |
| 153090 | *DAB2IP* | DAB2 interacting protein |
| 1749 | *DLX5* | distal-less homeobox 5 |
| 861 | *RUNX1* | runt-related transcription factor 1 |
| 6348 | *CCL3* | chemokine (C-C motif) ligand 3 |
| 10911 | *UTS2* | urotensin 2 |
| 5947 | *RBP1* | retinol binding protein 1, cellular |
| 25791 | *NGEF* | neuronal guanine nucleotide exchange factor |
| 5029 | *P2RY2* | purinergic receptor P2Y, G-protein coupled, 2 |
| **biological process connective tissue development**  **GO:0061448**  **C=177;O=10;E=2.18;R=4.60;rawP=6.46e-05;adjP=0.0098** | | |
| Entrez ID | Gene symbol | Description |
| 2202 | *EFEMP1* | EGF containing fibulin-like extracellular matrix protein 1 |
| 4487 | *MSX1* | msh homeobox 1 |
| 6615 | *SNAI1* | snail homolog 1 (Drosophila) |
| 2251 | *FGF6* | fibroblast growth factor 6 |
| 3202 | *HOXA5* | homeobox A5 |
| 23213 | *SULF1* | sulfatase 1 |
| 9350 | *CER1* | cerberus 1, cysteine knot superfamily, homolog (Xenopus laevis) |
| 3207 | *HOXA11* | homeobox A11 |
| 2300 | *FOXL1* | forkhead box L1 |
| 3213 | *HOXB3* | homeobox B3 |
| **biological process developmental process**  **GO:0032502**  **C=3993;O=71;E=49.09;R=1.45;rawP=0.0001;adjP=0.0114** | | |
| Entrez ID | Gene symbol | Description |
| 3851 | *KRT4* | keratin 4 |
| 358 | *AQP1* | aquaporin 1 (Colton blood group) |
| 5407 | *PNLIPRP1* | pancreatic lipase-related protein 1 |
| 4908 | *NTF3* | neurotrophin 3 |
| 433 | *ASGR2* | asialoglycoprotein receptor 2 |
| 2152 | *F3* | coagulation factor III (thromboplastin, tissue factor) |
| 220 | *ALDH1A3* | aldehyde dehydrogenase 1 family, member A3 |
| 9350 | *CER1* | cerberus 1, cysteine knot superfamily, homolog (Xenopus laevis) |
| 6935 | *ZEB1* | zinc finger E-box binding homeobox 1 |
| 153090 | *DAB2IP* | DAB2 interacting protein |
| 6348 | *CCL3* | chemokine (C-C motif) ligand 3 |
| 7068 | *THRB* | thyroid hormone receptor, beta |
| 6954 | *TCP11* | t-complex 11, testis-specific |
| 3987 | *LIMS1* | LIM and senescent cell antigen-like domains 1 |
| 2251 | *FGF6* | fibroblast growth factor 6 |
| 1240 | *CMKLR1* | chemokine-like receptor 1 |
| 79652 | *TMEM204* | transmembrane protein 204 |
| 2444 | *FRK* | fyn-related kinase |
| 3641 | *INSL4* | insulin-like 4 (placenta) |
| 6927 | *HNF1A* | HNF1 homeobox A |
| 2670 | *GFAP* | glial fibrillary acidic protein |
| 60529 | *ALX4* | ALX homeobox 4 |
| 3199 | *HOXA2* | homeobox A2 |
| 199699 | *DAND5* | DAN domain family, member 5 |
| 1749 | *DLX5* | distal-less homeobox 5 |
| 861 | *RUNX1* | runt-related transcription factor 1 |
| 5947 | *RBP1* | retinol binding protein 1, cellular |
| 768 | *CA9* | carbonic anhydrase IX |
| 5047 | *PAEP* | progestagen-associated endometrial protein |
| 50964 | *SOST* | sclerostin |
| 122953 | *JDP2* | Jun dimerization protein 2 |
| 27329 | *ANGPTL3* | angiopoietin-like 3 |
| 4632 | *MYL1* | myosin, light chain 1, alkali; skeletal, fast |
| 6929 | *TCF3* | transcription factor 3 (E2A immunoglobulin enhancer binding factors E12/E47) |
| 5552 | *SRGN* | serglycin |
| 9745 | *ZNF536* | zinc finger protein 536 |
| 23213 | *SULF1* | sulfatase 1 |
| 55224 | *ETNK2* | ethanolamine kinase 2 |
| 5493 | *PPL* | periplakin |
| 3207 | *HOXA11* | homeobox A11 |
| 6414 | *SEPP1* | selenoprotein P, plasma, 1 |
| 9514 | *GAL3ST1* | galactose-3-O-sulfotransferase 1 |
| 2202 | *EFEMP1* | EGF containing fibulin-like extracellular matrix protein 1 |
| 56913 | *C1GALT1* | core 1 synthase, glycoprotein-N-acetylgalactosamine 3-beta-galactosyltransferase, 1 |
| 5133 | *PDCD1* | programmed cell death 1 |
| 57549 | *IGSF9* | immunoglobulin superfamily, member 9 |
| 5728 | *PTEN* | phosphatase and tensin homolog |
| 8871 | *SYNJ2* | synaptojanin 2 |
| 10911 | *UTS2* | urotensin 2 |
| 1586 | *CYP17A1* | cytochrome P450, family 17, subfamily A, polypeptide 1 |
| 5029 | *P2RY2* | purinergic receptor P2Y, G-protein coupled, 2 |
| 646 | *BNC1* | basonuclin 1 |
| 10678 | *B3GNT2* | UDP-GlcNAc:betaGal beta-1,3-N-acetylglucosaminyltransferase 2 |
| 4487 | *MSX1* | msh homeobox 1 |
| 6615 | *SNAI1* | snail homolog 1 (Drosophila) |
| 10203 | *CALCRL* | calcitonin receptor-like |
| 1271 | *CNTFR* | ciliary neurotrophic factor receptor |
| 252995 | *FNDC5* | fibronectin type III domain containing 5 |
| 2011 | *MARK2* | MAP/microtubule affinity-regulating kinase 2 |
| 2300 | *FOXL1* | forkhead box L1 |
| 3213 | *HOXB3* | homeobox B3 |
| 5788 | *PTPRC* | protein tyrosine phosphatase, receptor type, C |
| 10677 | *AVIL* | advillin |
| 7547 | *ZIC3* | Zic family member 3 |
| 27122 | *DKK3* | dickkopf 3 homolog (Xenopus laevis) |
| 3202 | *HOXA5* | homeobox A5 |
| 7038 | *TG* | thyroglobulin |
| 940 | *CD28* | CD28 molecule |
| 5026 | *P2RX5* | purinergic receptor P2X, ligand-gated ion channel, 5 |
| 25791 | *NGEF* | neuronal guanine nucleotide exchange factor |
| 54583 | *EGLN1* | egl nine homolog 1 (C. elegans) |
